# The ferroptotic effect of NRF2-GCLM signaling axis derived by radiotherapy of esophageal squamous cell cancer: the vivo study

**DOI:** 10.1038/s41598-025-10414-2

**Published:** 2025-07-24

**Authors:** Yiliyaer Nuerrula, Zhaoyuan Xue, Aidiye Tiliwalidi, Xueling Xiao, Zihao Dong, Jingkun Liu, Mayinur Eli

**Affiliations:** 1 Department of Oncology, The First Affiliated Hospital of Xinjiang Medical University, No. 137, Liyushan Road, Urumqi, 830011 China; 2 Department of Oncology, The Third Affiliated Hospital of Xinjiang Medical University, Urumqi, 830011 China

**Keywords:** NRF2, GCLM, ESCC, Radiotherapy, Prognosis, Cell line-derived xenograft

## Abstract

Nuclearfactor erythroidderived 2-like 2(NRF2) is a major regulator of the body’s antioxidant defense system and a key protein in the process of ferroptosis, which is upregulated in a variety of malignancies. This study aimed to investigate the role of NRF2 in the pathogenesis and progression of Esophageal squamous cell carcinoma (ESCC) from the in vivo and clinical levels. The expression levels of NRF2, Glutamate-Cysteine Ligase Modifier Subunit (GCLM) and Glutathione Peroxidase 4 (GPX4) in ESCC and adjacent normal tissues were detected by immunohistochemistry in 61 tissue biopsies collected from patients diagnosed with ESCC. The xenograft model was used to detect the growth of nude mouse tumors and the changes of ferroptosis-related indexes in different experimental groups. Co-immunoprecipitation was used to demonstrate the downstream interacting proteins of NRF2. The results showed that the expression of NRF2, GCLM and GPX4 was increased in ESCC compared with adjacent non-tumor tissues, and the high expression of NRF2, GCLM and GPX4 was significantly associated with poor prognosis. NRF2 overexpression promotes changes in tumor growth and ferroptosis-related markers in xenograft models. In addition, NRF2 overexpression was associated with upregulation of GCLM and GPX4. CO-IP demonstrated that GCLM is a downstream protein of NRF2. Bio-informatics analysis showed that GCLM was differentially expressed in a variety of tumors, and was significantly correlated with the prognosis of patients, as well as the infiltration of a variety of immune cells. Finally, GCLM promotes tumor growth and radiotherapy resistance to ESCC in vivo, and can therefore be used as a molecular target for tumor therapy.

**Supplementary Information:**

The online version contains supplementary material available at 10.1038/s41598-025-10414-2.

## Introduction

Cancer is a major social, public health and economic problem in the twenty-first century, with nearly one in six deaths (16.8%) and one in four deaths (22.8%) worldwide due to noncommunicable diseases (NCDs)^1^. The global burden of cancer in 2022 is explored based on the latest GLOBOCAN estimates produced by the International Agency for Research on Cancer (IARC) and served as the Global Cancer Observatory as today’s cancer spread^2^^,^^3^. In 2022, there were an estimated 20 million new cases and 9.7 million cancer deaths worldwide. Esophageal cancer, a common malignancy worldwide^4^, is the 11th most commonly diagnosed cancer in the world, especially in China^2^, and is not only a major public health challenge, but also has a profound impact on socio-economic development. There were an estimated 511,000 new cases and 445,000 deaths in 2022^3^. East Asia and East Africa have the highest prevalence, and Malawi has the highest incidence in both men and women worldwide^5^. Smoking and alcohol consumption are major risk factors for squamous cell carcinoma. In recent years, with the advancement of medical technology and increased health awareness, the diagnosis and treatment of esophageal cancer have improved significantly, but its morbidity and mortality remain high^5^.

Ferroptosis is an iron-dependent and lipid peroxidation-driven cell death cascade with mitochondria in ferroptosis cells shrinking significantly as membrane density increases as its unique morphological alteration^6^^,^^7^. NRF2, as the star molecule in ferroptosis, is a central regulator of intracellular redox homeostasis. It encodes a transcription factor that is a member of a small family of basic leucine zipper proteins that regulates genes for other antioxidant response elements, thereby attenuating cellular damage caused by reactive oxygen species and electrophiles, keeping cells in a steady state^8^^,^^9^. Numerous studies have shown that aberrant inhibition of ferroptosis has been found in cancer cases, promoting its progression and metastasis^10^. Therefore, targeting NRF2-related signaling pathways to induce or inhibit ferroptosis has emerged as a great potential therapy for the fight against cancer and radiotherapy resistance^11–13^.

As one of the main treatment modalities for cancer, radiotherapy has greatly improved the prognosis and survival of patients. However, clinically, some cancer patients will gradually become resistant to radiotherapy, resulting in tumor recurrence and metastasis^14–18^. With the deepening of research, tumor cells may mediate the generation of radiotherapy resistance in a variety of ways, including interfering with the tumor cell cycle, activating DNA repair pathways, and cancer stem cells altering the tumor microenvironment and the presence of cell differentiation and immunosuppressive microenvironment^19–22^. In our previous experiments, in vitro experiments verified the important role of the NRF2-mediated ferroptosis signaling pathway in esophageal squamous cell carcinoma radiotherapy resistance, discovered a new downstream molecular target, GCLM, and investigated its potential feasibility in improving esophageal squamous cell carcinoma radiotherapy resistance^23^.

Although we have validated in vitro experiments to improve the sensitivity of esophageal squamous cell carcinoma to radiation therapy by targeting NRF2, we lack further research in a complex in vivo environment, so it is increasingly urgent and important to explore whether GCLM can be used as a new and effective molecular target to intervene and the crosstalk between GCLM and the immune system.

## Materials and methods

### Human tissue specimens

This study was approved (approval number: 20220309-106) and monitored by the Ethics Committee of the First Affiliated Hospital of Xinjiang Medical University (Urumqi, China). Informed consent has been obtained from all donors, and data are analyzed anonymously throughout the study. According to the criteria of the World Health Organization and the Chinese Medical Association, a total of 61 patients diagnosed with ESCC were included in this study. None of the patients received chemoradiotherapy or radiotherapy prior to surgery. Patients are followed up through outpatient, inpatient, and telephone monitoring. The mean follow-up period was one month, ending in May 2024.

### Western blotting

Protein extracts were prepared by cell lysis using radioimmunoprecipitation assay buffer (RIPA, Beijing Solarbio Science & Technology Co., Ltd.). Protein concentration was determined using the BCA protein assay kit (Thermo Fisher Scientific, Inc.), and 20 mg protein extract was separated by 10% sodium dodecyl sulfate–polyacrylamide gel electrophoresis (SDS-PAGE) followed by transfer onto a polyvinylidene fluoride (PVDF) microporous membrane (MilliporeSigma). Block with 5% skimmed milk powder for 2 h and then use antibodies for blocking. The proteins were identified using antibodies against NRF2 (1:1000 dilution, 80593-1-RR, Proteintech), GCLM (1:1000 dilution, R382192, ZEN-BIOSCIENCE), glutathione peroxidase 4 (GPX4) (1:1000 dilution, 381958, ZEN-BIOSCIENCE), and β-actin (1:5000 dilution, bs-0061R, Bioss).

### Animal model

Animal experiments were approved (approval number: IACUC-JT-20231121-33) and supervised by the Animal Ethics Committee of the First Affiliated Hospital of Xinjiang Medical University. Thirty-six female 3- to 4-week-old BALB/c nude mice weighing 13–15 g were purchased from Charles River Laboratories and housed in a specific pathogen-free environment in a xenograft model. Nude mice are housed in individually ventilated cages with room temperature 24 °C and 70% relative humidity. Nude mice can drink filtered tap water and feed ad libitum on a strict 12-h light/dark cycle. The animal laboratory was cleaned twice a day and disinfected with UV light for 1 h per week. Three days before nude mice received radiotherapy, on the day of radiotherapy, and three days after radiotherapy, each group of nude mice was intraperitoneally injected with 100 ul of normal saline, Fer-1 or TBHQ suspension according to the group. Provide humane care for laboratory animals in accordance with institutional animal care guidelines. The humane endpoint is in place, and if an animal loses 20% of its body weight or 10% of its body weight, and at the same time develops hirsutism, anorexia, or decreased vitality, they are killed. All mice were sacrificed by cervical dislocation, which met the implementation standards of euthanasia in the laboratory animal center of the First Affiliated Hospital of Xinjiang Medical University.

### Immunohistochemistry (IHC)

Cut the embedded tissue sample into 4 μm slices and bake the slices at 60 °C for two hours. The sections were immersed in xylene and different concentrations of ethanol, and sequentially deparaffinized and rehydrated. Retrieval antigens were refreshed by boiling with 10 mM pH 6.0 sodium citrate buffer and cooled to room temperature. Use the primary antibody with Western blotting and incubate for 2 h at room temperature. All tissue sections were stained with DAB solution and hematoxylin.

### Indicators of ferroptosis detection

Nude mice were sacrificed, 0.1 g of tissue samples were taken for grinding after tumor tissue isolation, and GSH and MDA content were determined according to the kit instructions(all from Solarbio, Beijing, China).

### Cell culture and drugs

The human ESCC cell lines KYSE150 and KYSE450, all obtained from the First Affiliated Hospital of Xinjiang Medical University, underwent cellular short tandem repeat (STR) identification before utilization. The intervention is performed in an in vivo nude mouse model with the ferroptosis inhibitor Ferrostatin-1(Fer-1, HY-100579, MedChemExpress, USA) and the NRF2 agonist Tert-butylhydroquinone(TBHQ, HY-100489, MedChemExpress, USA).

### Bioinformatics

We downloaded the STAR-counts data and corresponding clinical information of all tumors from the TCGA database (https://portal.gdc.cancer.gov), then extracted the data in TPM format, normalized log2 (TPM + 1), and finally retained the samples with both RNAseq data and clinical information, and a total of 10,228 samples were retained for subsequent analysis. The GTEx data we use is from the V8 version, and the details can be found on the official GTEx website (https://gtexportal.org/home/datasets). To validate the reliability of immune score evaluation, the immuneeconv package was utilized. Analysis and visualization were conducted using the R software ggClusterNet package. At the same time, the STAR-counts data and corresponding clinical information of 82 cases of esophageal squamous cell carcinoma were downloaded from the TCGA database. The genes contained in the corresponding pathway were collected and analyzed using the GSVA package of R software, and the parameter method = ‘ssgsea’ was selected for single-sample gene set enrichment analysis (ssGSEA). Finally, Spearman correlation analysis was used to study the correlation between gene expression and pathway scores. All the aforementioned analyses and R packages were performed using R software version v4.1.3 (R Foundation for Statistical Computing, 2022). A *P* value < 0.05 was considered statistically significant.

### Statistical analysis

Data were presented as the mean ± standard deviation (SD). Chi-squared test and the Mann–Whitney U test was used to analyse the differences between the two groups and multiple groups. Patient survival was analysed using Kaplan–Meier survival analysis and the log-rank test. The Spearman correlation method was used to calculate the correlation between NRF2, GCLM and GPX4 and lymph node metastasis. In cases where the variance is uneven or does not follow a normal distribution, the Kruskal–Wallis test is used. GraphPad Prism 9 software was utilized for data analysis and graphical representation. A *P* value < 0.05 was considered statistically significant.

## Results

### Expression of ferroptosis-related genes in patients with ESCC

**Fig. 1:**
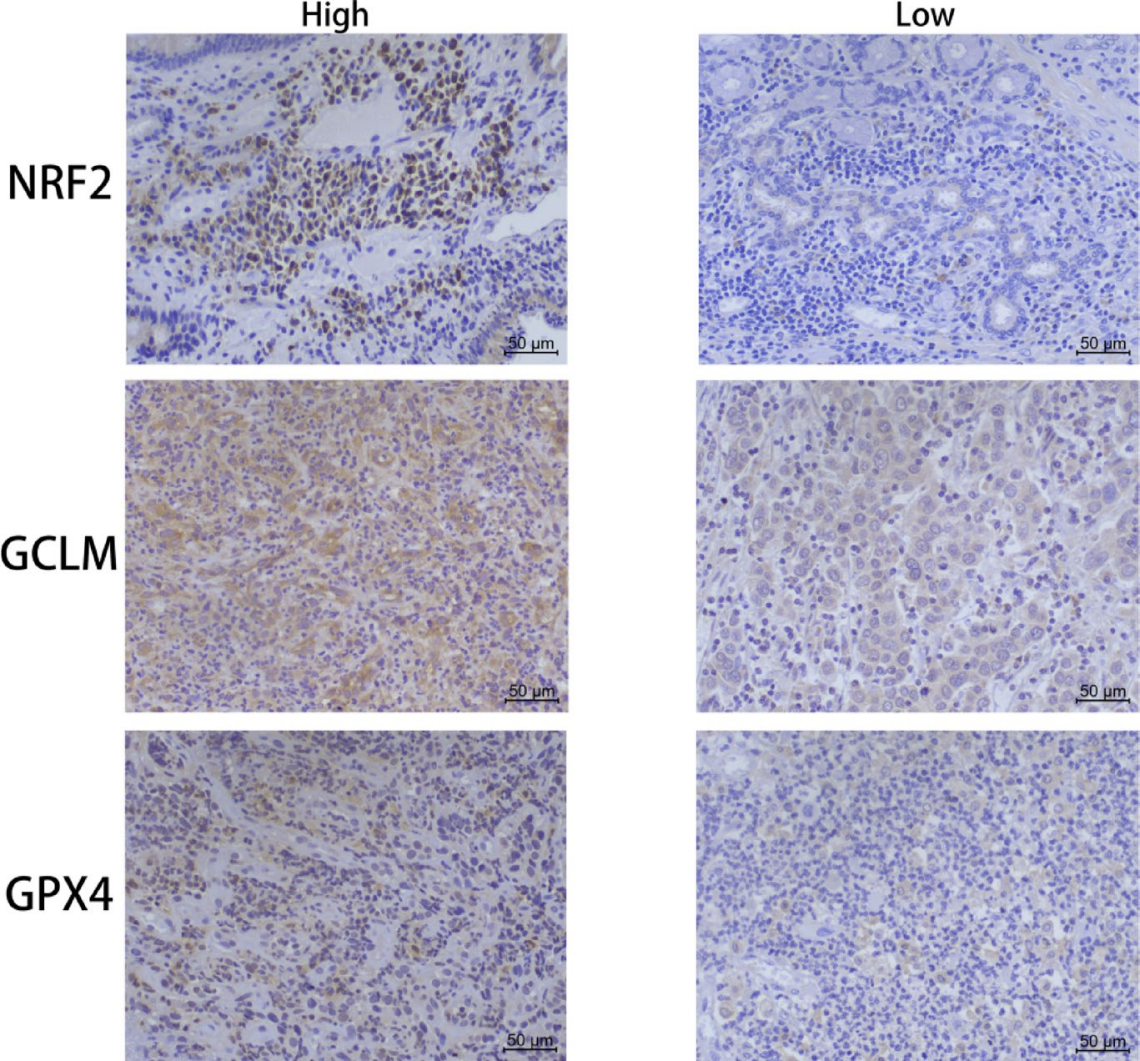
IHC staining of NRF2, GCLM, and GPX4 in ESCC tissue and adjacent normal tissue.

In this study, immunohistochemistry was used to detect the expression of NRF2 nuclear (representing NRF2 activation), GCLM and GPX4 expression, and the correlation with TNM stage was analyzed (Fig. 1). In patients with T3-T4 stage, the expression levels of NRF2, GCLM and GPX4 were increased, which were *p* = 0.010, *p* < 0.001 and *p* = 0.007 (all *p* < 0.05), and the patients with high expression of NRF2, GCLM and GPX4 tended to have high lymph node metastasis rates of *p* = 0.036, *p* < 0.001 and *p* = 0.001 (all *p* < 0.05), and there was a significant correlation between distant metastasis and the expression of all three, which were *p* = 0.015, *p* < 0.001 and *p* = 0.001 (Table 1, all *p* < 0.05), the above results showed that the higher the TNM stage, the higher the expression levels of NRF2, GCLM and GPX4, all of which were statistically significant (all *p* < 0.05), suggesting that the expression of the above genes was related to tumor progression and metastasis of ESCC.

**Table 1:** The expressions of NRF2, GCLM and GPX4 were correlated with TNM stage in 61 patients.

Variable	T		N		M	
	T1-2	T3-4	N0	N1-3	M0	M1
NRF2						
Low expression	15 (65.2)	12 (31.6)	16 (59.3)	11 (32.4)	18 (60)	9 (29)
High expression	8 (34.8)	26 (68.4)	11 (40.7)	23 (67.6)	12 (40)	22 (71)
* P* value	0.01		0.036		0.015	
GCLM						
Low expression	18 (78.3)	12 (31.6)	21 (77.8)	9 (26.5)	21 (70)	9 (29)
High expression	5 (21.7)	26 (68.4)	6 (22.2)	25 (73.5)	9 (30)	22 (71)
*P* value	< 0.001		< 0.001		0.001	
GPX4						
Low expression	16 (69.6)	13 (34.2)	19 (70.4)	10 (29.4)	21 (70)	8 (25.8)
High expression	7 (30.4)	25 (65.8)	8 (29.6)	24 (70.6)	9 (30)	23 (74.2)
*P* value	0.007		0.001		0.001	

### Clinical significance of ferroptosis-related genes in ESCC

**Fig. 2:**
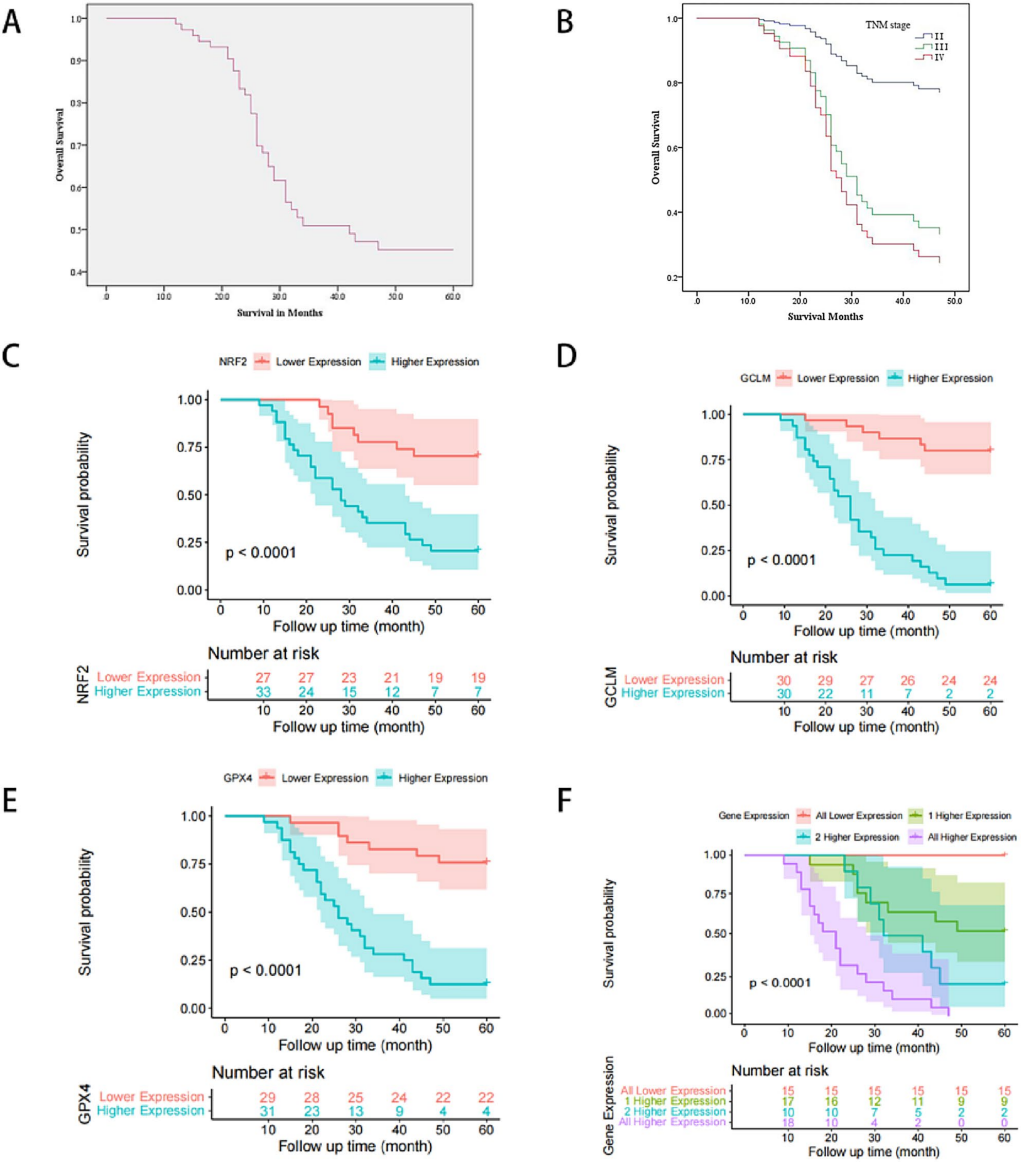
(**A**) 3-year and 5-year OS survival curves in 61 patients with ESCC; (**B**) Survival curves of 61 patients with ESCC at different TNM stages; OS survival curves for nuclear NRF2 (**C**), GCLM (**D**), GPX4 (**E**) and their combined (**F**) expression.

We further used univariate and multivariate Cox regression analyses to investigate the possible prognosis-related factors in 61 participants (Table 2). Univariate analysis showed that the gene expressions of T, N and M stage, radiotherapy dose, lesion location, lesion length, NRF2, GCLM, and GPX4 were all associated with OS (all *P* < 0.05), but gender, age, smoking history, alcohol history, and radiotherapy mode may not be related to OS, and OS was used as the dependent variable. Multivariate Cox regression analysis showed that the expression levels of T, N and M stage, NRF2, GCLM and GPX4 genes were independent prognostic factors (Fig. 2, all *P* < 0.05).

**Table 2:** Univariate and multivariate analyses of survival prognosis in 61 patients.

Variable	Univariate analysis		Multivariate analysis	
	HR (95%CI)	*P* value	HR (95%CI)	*P* value
Gender				
Male	1.000			
Female	0.61 (0.312–1.193)	0.148		
Age				
< 65	1.000			
≥ 65	1.207 (0.6–2.426)	0.598		
Smoking				
No	1.000			
Yes	1.625 (0.792–3.332)	0.185		
Drinking				
No	1.000			
Yes	1.693 (0.811–3.535)	0.161		
T				
T2	1.000		1.000	
T3-4	8.133 (2.847–23.233)	0.000	3.872 (1.194–12.557)	0.024
N				
N0	1.000		1.000	
N1-3	5.674 (2.438–13.207)	0.000	3.443 (1.288–9.204)	0.014
M				
M0	1.000		1.000	
M1	5.213 (2.411–11.268)	0.000	2.428 (1.022–5.769)	0.045
Radiation dose				
≥ 60 Gy	1.000		1.000	
< 60 Gy	4.388 (1.905–10.104)	0.001	1.118 (0.418–2.994)	0.824
Radiotherapy				
Radiotherapy alone	1.000			
Concurrent chemotherapy	0.93 (0.468–1.847)	0.835		
Location of the lesion				
Upper	1.000		1.000	
Middle	2.683 (0.770–9.344)	0.121	2.104 (0.487–9.090)	0.319
Low	5.296 (1.555–18.034)	0.008	2.722 (0.593–12.488)	0.198
Length				
< 5 cm	1.000		1.000	
≥ 5 cm	2.057 (1.035–4.091)	0.040	0.925 (0.341–2.513)	0.879
NRF2				
Low expression	1.000		1.000	
High expression	4.429 (2.000–9.805)	0.000	2.932 (1.166–7.372)	0.022
GCLM				
Low expression	1.000		1.000	
High expression	10.77 (4.362–26.591)	0.000	2.665 (1.005–7.067)	0.049
GPX4				
Low expression	1.000		1.000	
High expression	7.321 (3.141–17.067)	0.000	3.207 (1.187–8.663)	0.022

### Impact of the drugs on the growth of tumour xenografts from ESCC cells

**Fig. 3:**
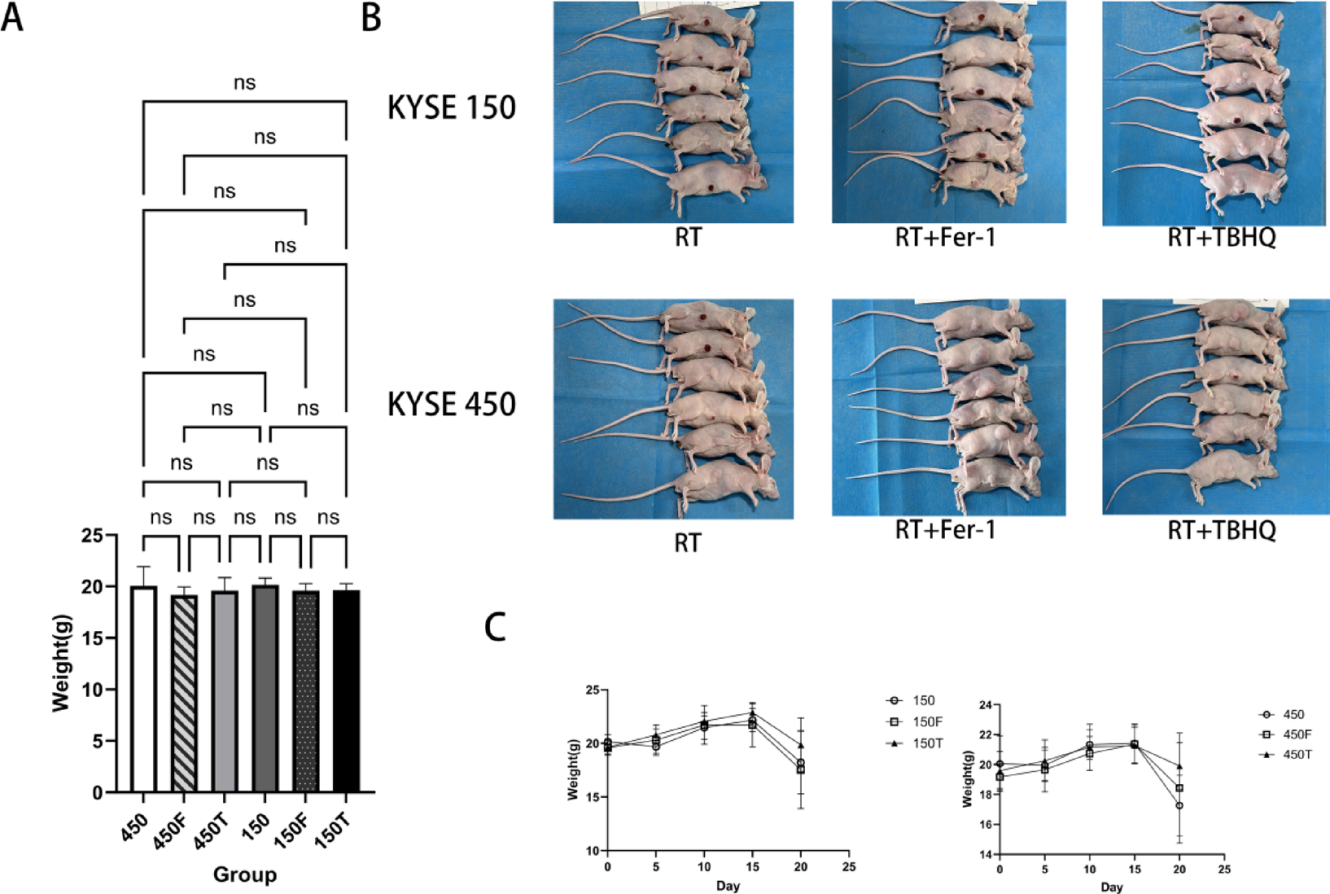
(**A**) Statistical analysis plot of 36 nude mice randomly divided into 6 groups; (**B**) Tumor formation was measured after subcutaneous tumorigenesis; (**C**) Body weight changes in KYSE150 and KYSE450 cell lines after injection with saline, Fer-1, and TBHQ, respectively.

**Fig. 4:**
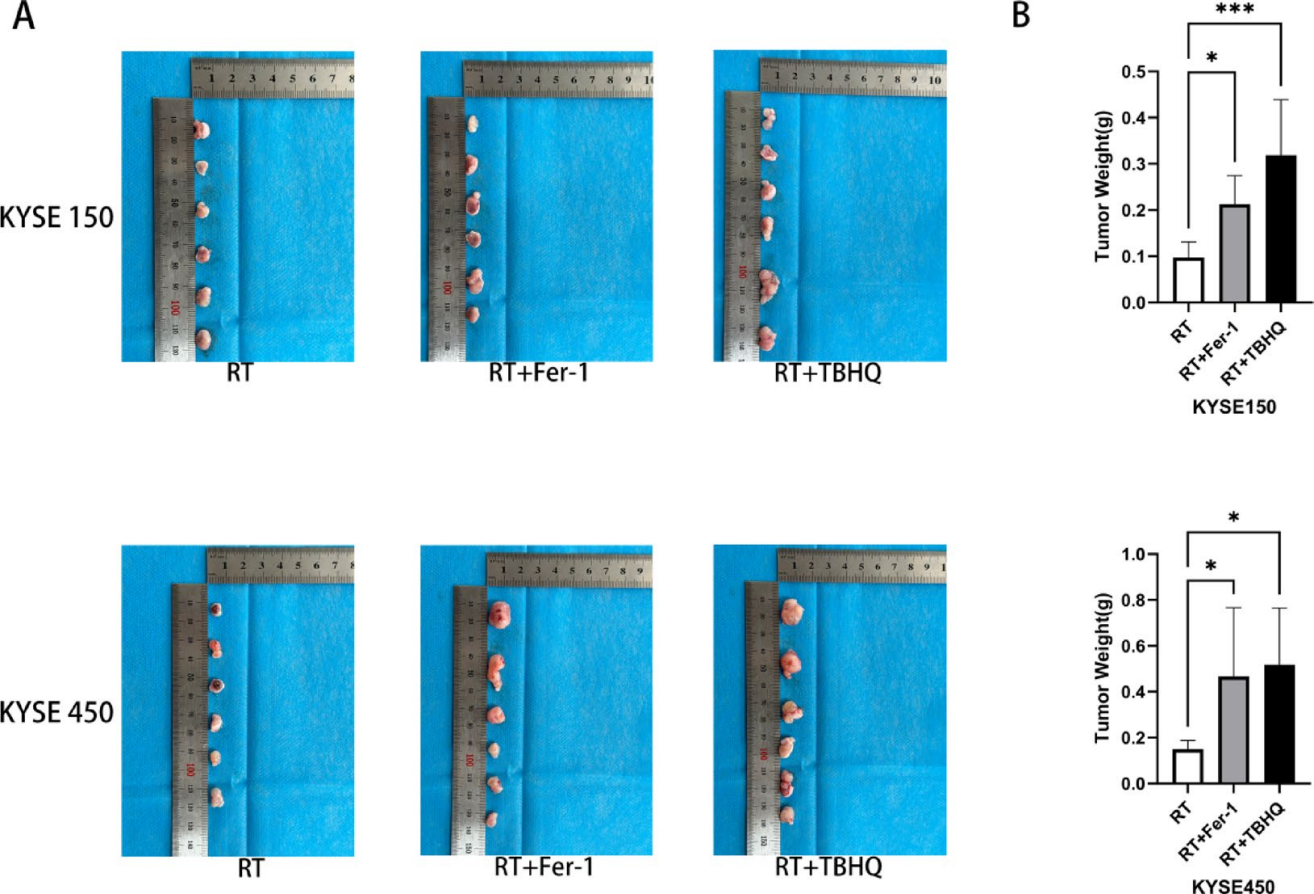
(**A**,**B**) nude mice were injected intradermally with KYSE150 and KYSE450 cell lines to measure tumor size and tumor weight in different experimental groups.

The role of NRF2 expression in tumorigenesis in vivo was investigated by analyzing tumor xenografts generated by intradermal injection of KYSE150 and KYSE450 cell lines in nude mice before and after injection with normal saline, ferroptosis inhibitor Fer-1 and NRF2 agonist TBHQ (Fig. 3). Intraperitoneal injection of Fer-1 and TBHQ resulted in a rapid increase in tumor weight, tumor growth, and tumor size in nude mice of two different esophageal cancer cell lines compared to the RT group (Fig. 4). These results suggest that elevated NRF2 in ESCC cells may not only promote cell proliferation, but also promote tumor growth in vivo.

### Detection of ferroptosis-related markers

**Fig. 5:**
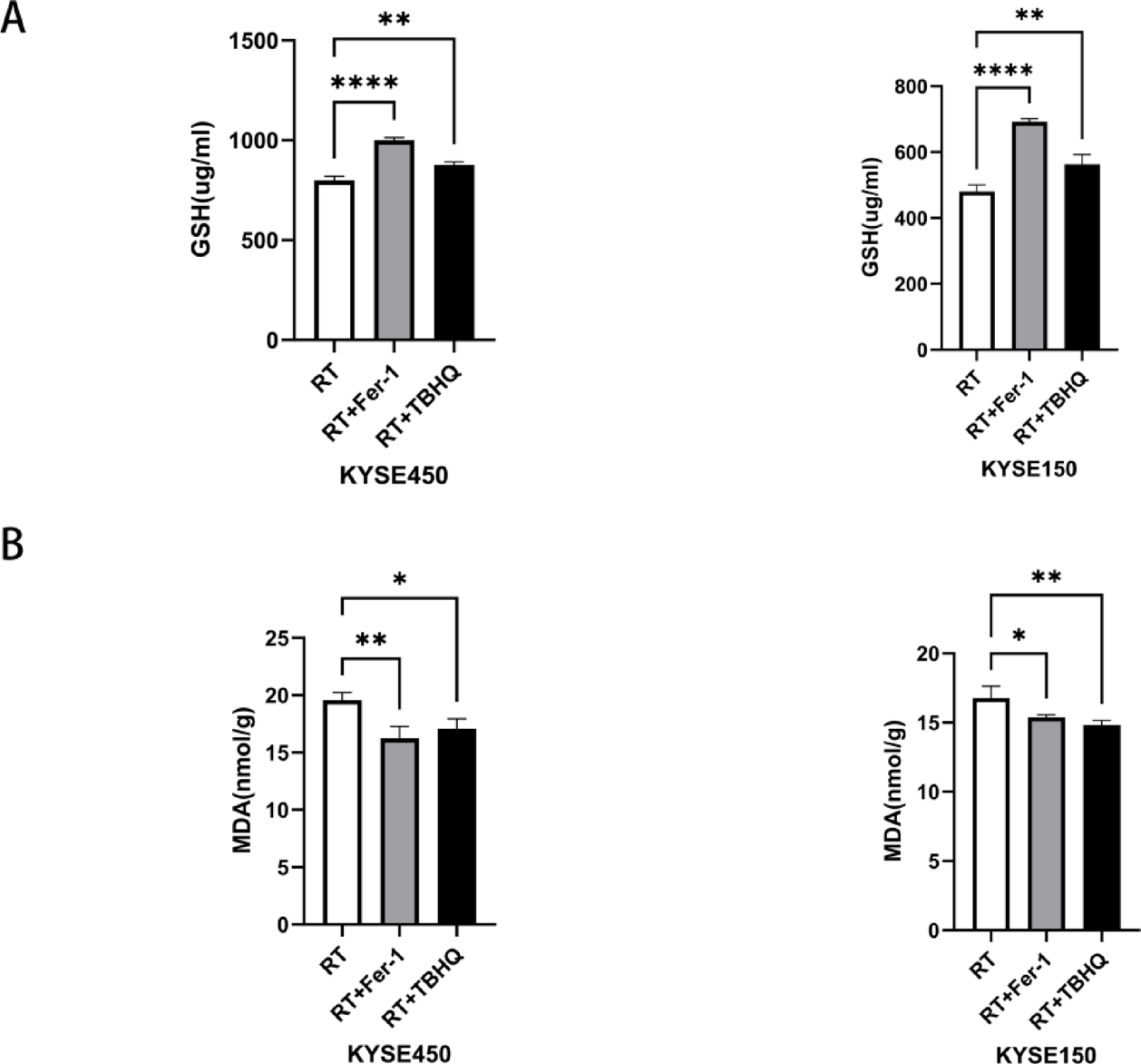
(**A**,**B**) Quantification of GSH and MDA expression levels in KYSE150 and KYSE450 cell lines across three different treatment modalities. *P < 0.05; **P < 0.01; ****P < 0.0001.

We looked at whether the effect on ferroptosis and thus changes in ferroptosis markers could be observed in vivo after interventions with RT, RT + Fer-1 or RT + TBHQ. The results showed that the lowest levels of GSH were found in nude mice inoculated with two different ESCC cell lines and radiotherapy only. However, after the intervention with RT + Fer-1 or RT + TBHQ, the content of GSH was significantly increased. Next, we examined MDA expression, and in three groups of nude mice with different intervention modalities, MDA content was significantly reduced in nude mouse tumor tissues treated with RT + Fer-1 or RT + TBHQ (Fig. 5).

### Changes in the protein expression profile related to ferroptosis after increased NRF2

**Fig. 6:**
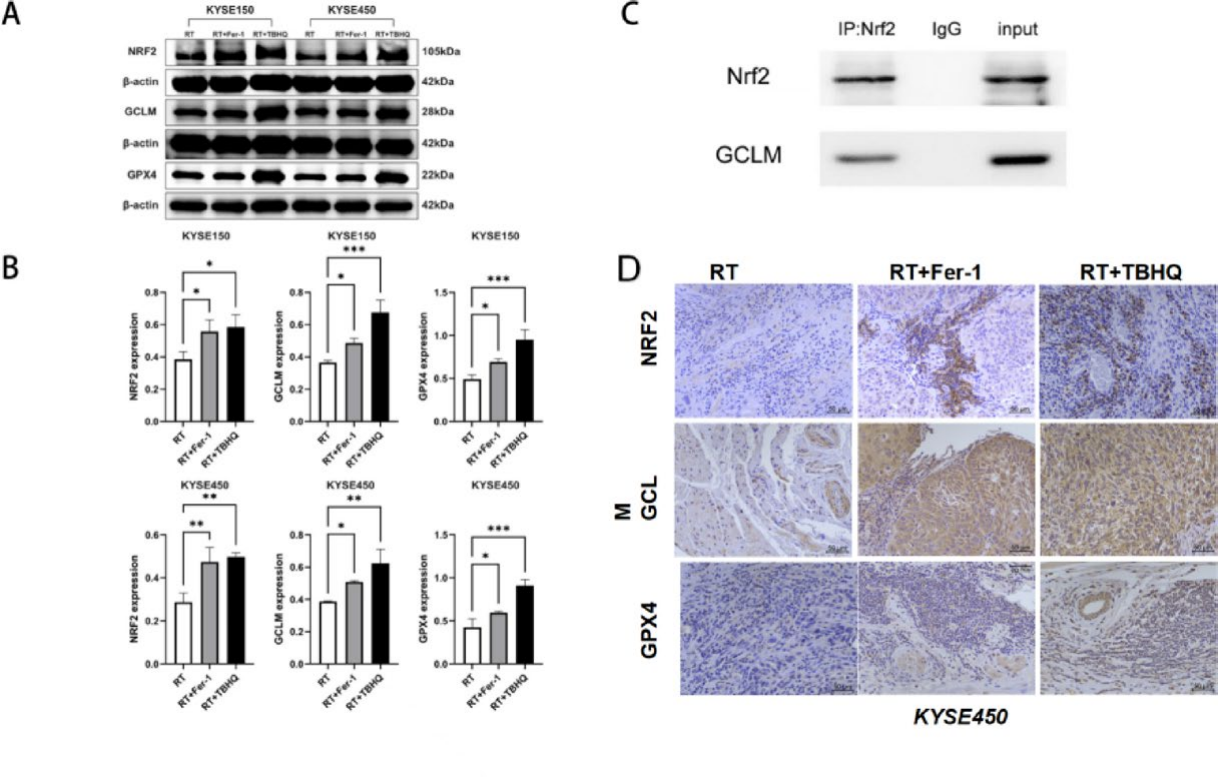
(**A**) Western blot analysis results displaying the expression levels of NRF2, GCLM, and GPX4 in the xenograft model, following three different treatment modalities; (**B**) Statistical analysis of western blot results; (**C**) Co-immunoprecipitation results of NRF2 and GCLM; (**D**) Differential expression of NRF2, GCLM, GPX4 in IHC after separate management of RT combination group from nude mice xenograft model.

Although in our previous experiments, the expression of ferroptosis-related proteins was studied in vitro, we did not know how they were expressed in vivo, so we took an appropriate amount of tumor tissue for western blot analysis. The results showed that the expression levels of NRF2, GCLM and GPX4 were significantly increased after intervention with RT + Fer-1 or RT + TBHQ compared with the RT group in tumor tissues of the two ESCC cell lines, and there were statistical differences in all of them. Simultaneously, the same confirmation was done with experiment of IHC method, which manifests higher expression of genes above compared to RT alone. Based on our experiments and bioinformatics analysis, GCLM was identified as a candidate for NRF2 downstream interactions. To verify the reliability of the conclusions, we performed a co-immunoprecipitation analysis to assess the binding potential of the two in nude mice. The tumor tissue of nude mice was thoroughly triturated, and the required immunoprecipitate before and after the immunoprecipitation step was extracted. Protein enrichment of anti-NRF2 antibodies reveals the interaction of NRF2 with GCLM in vivo under stress (Fig. 6).

### NRF2 downstream target GCLM-related pathway scores

**Fig. 7:**
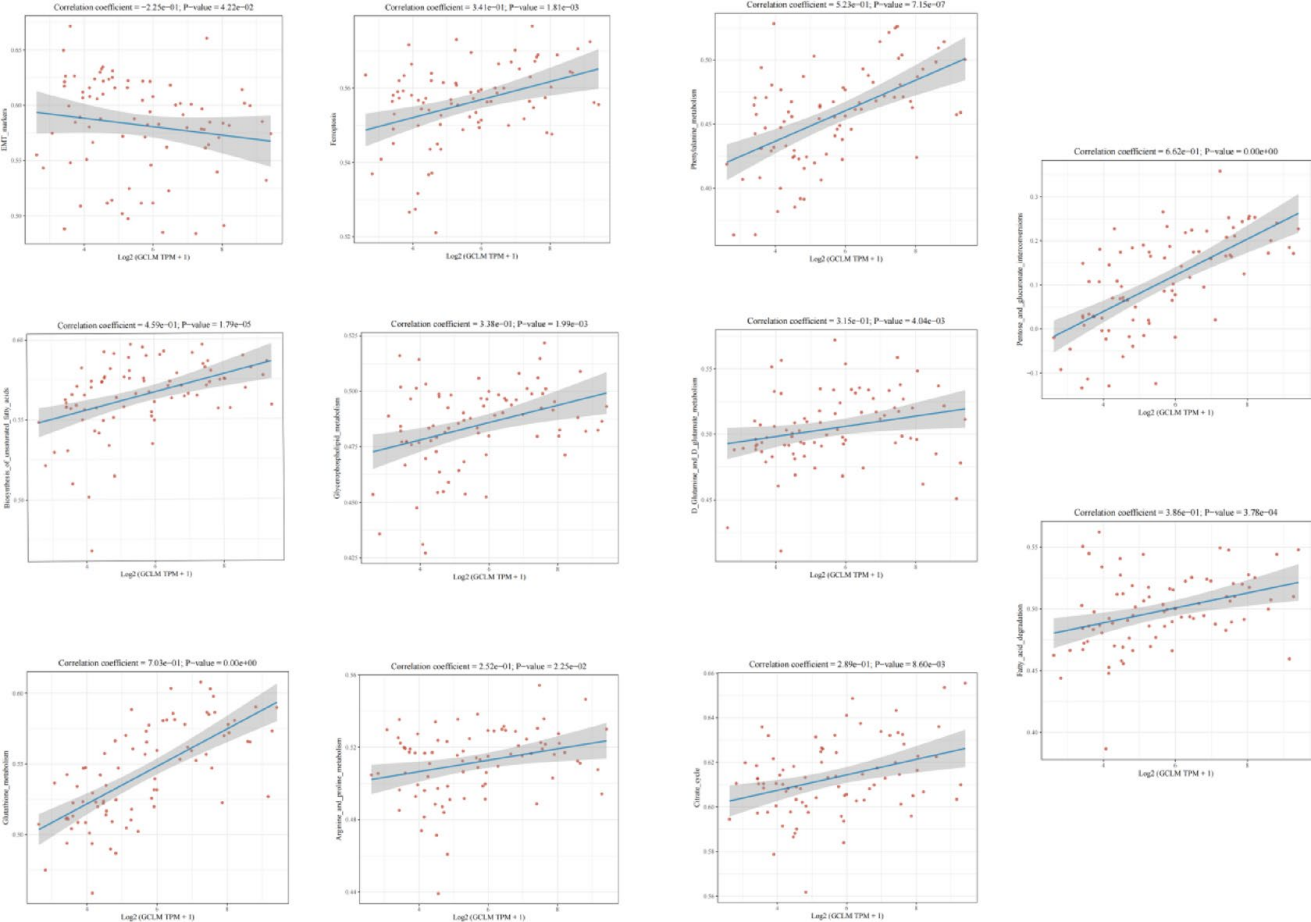
Spearrman correlation analysis plot between pathway score and GCLM expression.

We used the ssGSEA algorithm to calculate the enrichment fraction of GLCM expression in each pathway in ESCC sequentially, so as to establish the link between the sample and the pathway and determine the correlation with the pathway score. According to the Spearman correlation analysis, it can be found that the high expression of GCLM is significantly enriched in ferroptosis and glutathione metabolism, as well as in pathways involved in redox reaction, mitochondrial metabolism and EMT-related genes (Fig. 7).

### Immunocorrelation analysis of GCLM in ESCC

**Fig. 8:**
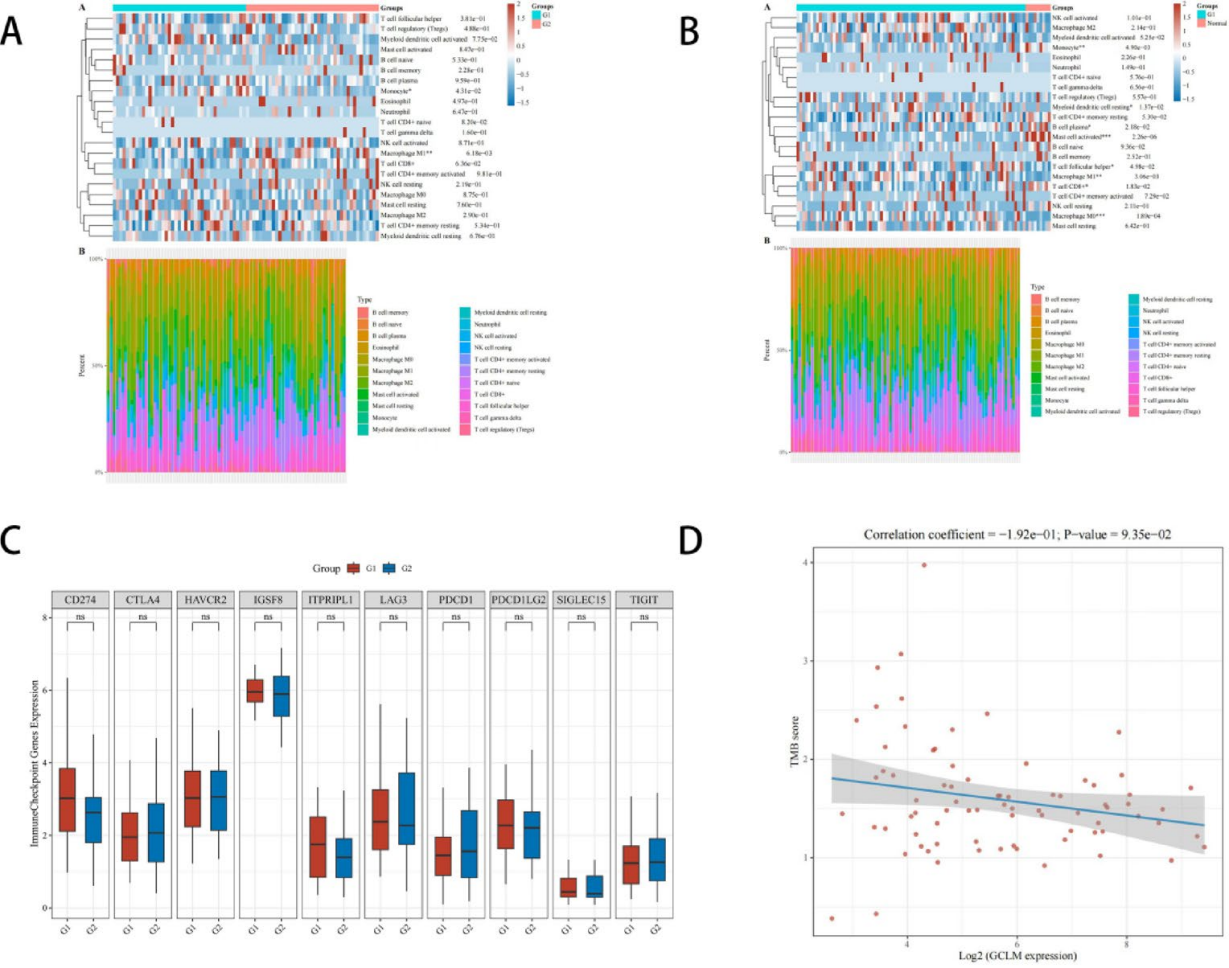
(**A**) Immune score heatmap of high- and low-expressing GCLMs in ESCC. G1: low expression group; G2: High expression group; (**B**) Immune score heatmap of ESCC and GCLM in normal tissues; (**C**) Expression distribution of immune checkpoint genes with high and low GCLM expression in ESCC. G1: low expression group; G2: High expression group; (**D**) Scatterplot and fitting line of Spearman correlation analysis between TMB and GCLM expression.

To verify the regulatory role of GCLM in radiotherapy resistance, and then to determine whether it can be used as an effective molecular target for intervention, we first obtained the distribution of GCLM gene immune scores in ESCC and normal tissues through bioinformatics analysis. The results showed that there were multiple immune cells infiltrating and regulating the tumor immune microenvironment, and then we divided the ESCC patients into two groups according to the expression of GCLM, and the heat map showed that the infiltration abundance of M1 macrophages was higher in the low expression group and more pronounced in the high expression group with monocyte infiltration. Subsequently, we examined the correlation between GCLM expression and immune checkpoints. The results showed that there was no significant difference in the expression distribution of immune checkpoint genes between the high-expression and low-expression sample groups in ESCC. It is well known that tumor mutational burden affects a patient’s responsiveness to immunotherapy, so we then examined the TMB of ESCC patients, and the scatter plot of Spearman’s correlation analysis showed that GCLM was associated with lower TMB in ESCC (Fig. 8).

### Pan-cancer analysis of GCLM in the TCGA database

**Fig. 9:**
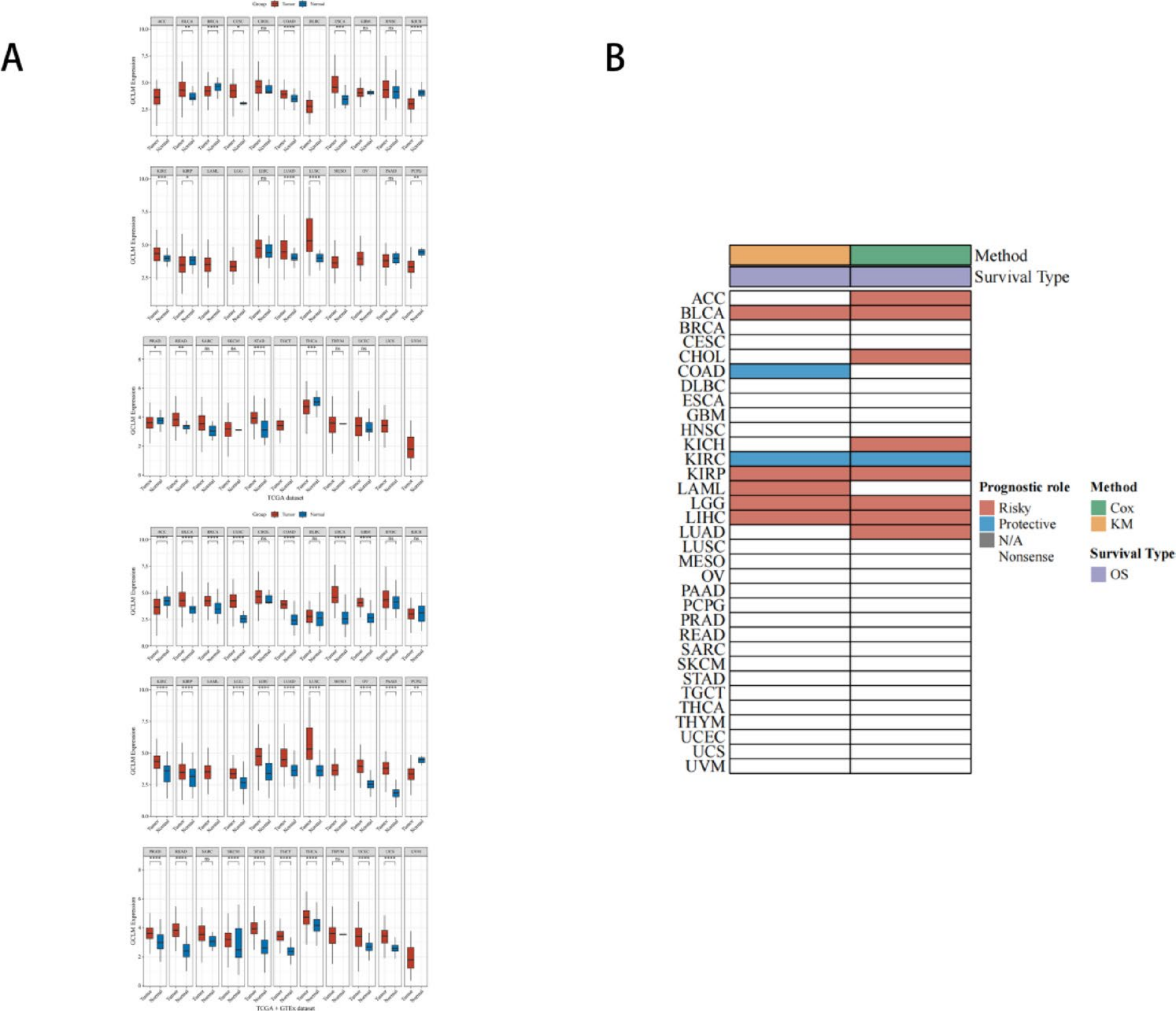
(**A**) Boxplots and dot plots of GCLM gene expression distribution in tumor tissues and normal tissues of 33 cancer types in the TCGA database; (**B**) Kaplan–Meier (KM) survival analysis and pan-cancer prognostic heat map of univariate Cox regression analysis for single-gene GCLM of 33 cancer types in the TCGA database.

Although GCLM has not shown satisfactory results for immune-related bioinformatics analysis, we investigated the role of GCLM in pan-cancer. We analyzed GCLM expression in 33 cancers using TCGA and GTEx databases, respectively, and boxplots showed that GCLM was highly expressed in nine cancers and low in six cancers. When GTEx data were included, we observed aberrant expression of the GCLM gene in more cancers. The pan-cancer prognostic heat map incorporates the results of Kaplan–Meier (KM) survival analysis and univariate Cox regression analysis. The results showed that higher GCLM expression showed a worse prognosis in nine different cancers; In the two cancers, COAD and KIRC, GCLM existed as a protective factor and showed a better prognosis (Fig. 9).

## Discussion

Ferroptosis is a novel form of iron-dependent cell death characterized by the accumulation of lipid peroxides on cell membranes. This pattern of cell death differs significantly from traditional forms of cell death, such as apoptosis, necrosis, and autophagy, in morphological and biochemical characteristics^24^. Iron plays an important role in tumor biology, and excess iron is closely related to the occurrence and progression of a variety of cancers^25^. Studies have shown that tumor cells often exhibit a high degree of iron dependence, which makes them more susceptible to ferroptosis in some cases^26^. In the tumor microenvironment, an imbalance in iron metabolism may lead to a deficiency of iron ions, thereby inhibiting the occurrence of ferroptosis^27^. Therefore, modulating iron pool levels within tumor cells is considered a potential therapeutic strategy. By targeting iron metabolism, researchers were able to enhance the sensitivity of tumor cells to ferroptosis, thereby improving the efficacy of cancer treatments^28^. For example, certain small molecule compounds, such as erastin, are able to induce ferroptosis, which in turn selectively destroys tumor cells^29^. In addition, there is a strong link between ferroptosis and tumor metastasis and response to treatment. Induction of ferroptosis may be an important strategy to overcome tumor metastasis and drug resistance^30^. In some studies, ferroptosis has been found to be able to modulate tumor-host interactions and play a role in anti-metastatic mechanisms^30^.But ferroptosis may also lead to resistance to radiation therapy in patients with esophageal cancer, which can affect their prognosis. Studies have shown that esophageal cancer stem cells are more susceptible to ferroptosis inducers than ordinary cancer cells, but their regulatory mechanisms are not fully understood^30^. Ferroptosis is a novel iron-dependent form of cell death that involves the accumulation of lipid peroxides, and esophageal cancer stem cells defend against this cell death by activating the Hsp27-GPX4 pathway^30^. Therefore, interventions targeting this protective mechanism may provide new therapeutic strategies for overcoming radiotherapy resistance. In addition, radiotherapy itself may lead to malnutrition and immunosuppression, which further affects treatment efficacy and patient survival^31^. In patients with esophageal cancer, improvement in nutritional status is strongly correlated with the tolerability and prognosis of radiotherapy^29^. Therefore, it is important to comprehensively consider the interaction between ferroptosis, nutritional status, and radiotherapy to find potential molecular targets to improve the treatment efficacy and survival rate of esophageal cancer patients.

NRF2, as the core star gene in the ferroptosis process, is a key transcription factor involved in the cellular response to oxidative stress, and its overactivation leads to esophageal squamous cell carcinoma (ESCC) resistance to radiotherapy. However, the potential signaling pathways of NRF2-mediated radiation resistance remain unclear. Studies have shown that NRF2 affects tumor growth and drug resistance by regulating antioxidant response and cellular metabolism in a variety of cancers^32,33^. In ESCC, high expression of NRF2 is strongly associated with resistance to chemotherapy and radiotherapy, and is associated with poor prognosis in patients^34,35^. In addition, NRF2 may also regulate the survival and proliferation of cancer cells through interactions with other signaling pathways, such as the mTORC1 and AKT pathways^36,37^. In terms of radiotherapy resistance, NRF2 has been found to promote DNA damage repair by activating the ATR-CHK1 signaling pathway, thereby enhancing cancer cell tolerance to radiotherapy^38^. This mechanism suggests that NRF2 not only plays a role in the antioxidant response, but may also play a key role in the DNA damage response^38^. Therefore, in-depth study of the role of NRF2 in ESCC and its signaling regulation mechanism, and the search for potential signaling regulatory pathways, will help to develop targeted therapy strategies against NRF2 to overcome radiotherapy resistance^39,40^.

In our previous experiments, we verified at the cellular level that NRF2 affects GPX4 expression by regulating the expression of GCLM, thereby influencing the resistance of esophageal cancer cells to radiotherapy. In esophageal cancer cells, activation of NRF2 can upregulate the expression of antioxidant enzymes, including glutathione synthetase (GCLM) and glutathione peroxidase 4 (GPX4), which play an important role in the cell’s antioxidant defense^41^. Studies have shown that overexpression of NRF2 correlates with radiotherapy tolerance in esophageal cancer cells, possibly due to its enhanced antioxidant capacity of cells, thereby reducing radiotherapy-induced oxidative damage^42^. During radiotherapy, the level of oxidative stress in esophageal cancer cells is significantly increased, and NRF2 enhances the cell’s resistance to oxidative damage by regulating the expression of GCLM and promoting glutathione synthesis^43^. We further investigated the reliability of targeting the NRF2-mediated ferroptosis signaling pathway at the in vivo and clinical levels. We verified that targeting NRF2 can promote the resistance of esophageal cancer cells to radiotherapy by regulating GCLM by establishing xenograft models derived from two different esophageal cancer cell lines in nude mice, and intervening with normal saline, ferroptosis inhibitors and NRF2 agonists in nude mice under the condition of simultaneous radiotherapy. More importantly, there was no statistically significant difference in the weight of nude mice depending on the intervention, and there was no significant effect on the viability of nude mice. Therefore, the regulation of the NRF2/GCLM/GPX4 pathway may provide a new therapeutic strategy for overcoming radiotherapy resistance in esophageal cancer cells.

The regulatory subunit of glutamate-cysteine ligase (GCLM) is thought to be a pro-tumor gene, and its high expression is strongly associated with poor prognosis in cancer patients. Studies have shown that GCLM exhibits upregulation in a variety of cancer types, especially in aggressive tumors^44^. For example, in breast cancer and glioblastoma, the expression level of GCLM is inversely correlated with patient survival^45^. In addition, overexpression of GCLM may lead to tumor progression by enhancing the antioxidant capacity of cells and promoting the survival and proliferation of tumor cells^46^. In some studies, high expression of GCLM has been recognized as a hallmark of cancer stem cell characterization, which further supports its important role in tumorigenesis and progression^47^. In addition, the expression level of GCLM is closely related to immune cell infiltration in the tumor microenvironment, which further affects tumor progression and patient prognosis^48^. By inhibiting the expression or function of GCLM, it may reduce the antioxidant capacity of tumor cells, thereby enhancing sensitivity to chemotherapy and radiotherapy^48^. Through bioinformatics analysis of GCLM and immunohistochemical results of ESCC patients, we found that GCLM was highly expressed in ESCC and was significantly negatively correlated with the prognosis of patients. However, immunoscopic scores and immune checkpoint analysis were performed on GCLM expression in patients with ESCC, and the results showed that there was no statistical difference between GCLM and multiple immune checkpoints, suggesting that patients with high GCLM expression would not benefit from immunotherapy. We further investigated the reasons for this phenomenon and demonstrated that GCLM expression leads to lower TMB in patients with ESCC, thereby promoting immune escape. Therefore, the high expression of GCLM is not only an important part of tumor biology, but may also provide new targets and strategies for the treatment of cancer. However, more research is needed to clarify the crosstalk between the tumor immune microenvironment mediated by GCLM and ferroptosis, so as to restore the sensitivity of ESCC patients to radiotherapy and prolong the survival of patients.

With a relatively small sample content, the resulting differences in survival data and gene expression abundance may be too significant, and a larger sample size may more balance the relative one-sidedness of the experimental results. Detection of the expression of specific cellular components in the tumor immune microenvironment by flow cytometry after animal experiments may provide better space to explain the mechanism of radiotherapy-mediated targeted ferroptosis in immune regulation.

## Electronic supplementary material

Below is the link to the electronic supplementary material.

Supplementary Material 1

## Data Availability

All data generated or analysed during this study are included in this published article. RNA-seq data and clinical information for the ESCC cohort were downloaded from the Genome Data Sharing (GDC) data portal of the Cancer Genome Atlas (TCGA) database (https://portal.gdc.cancer.gov/).
