# Supplementary material for: The ferroptotic effect of NRF2-GCLM signaling axis derived by radiotherapy of esophageal squamous cell cancer: the vivo study
Source: Sci Rep. 2025 Jul 24;15:26917. doi: 10.1038/s41598-025-10414-2 (PMC12290101; doi:10.1038/s41598-025-10414-2)

NRF2(whole film)


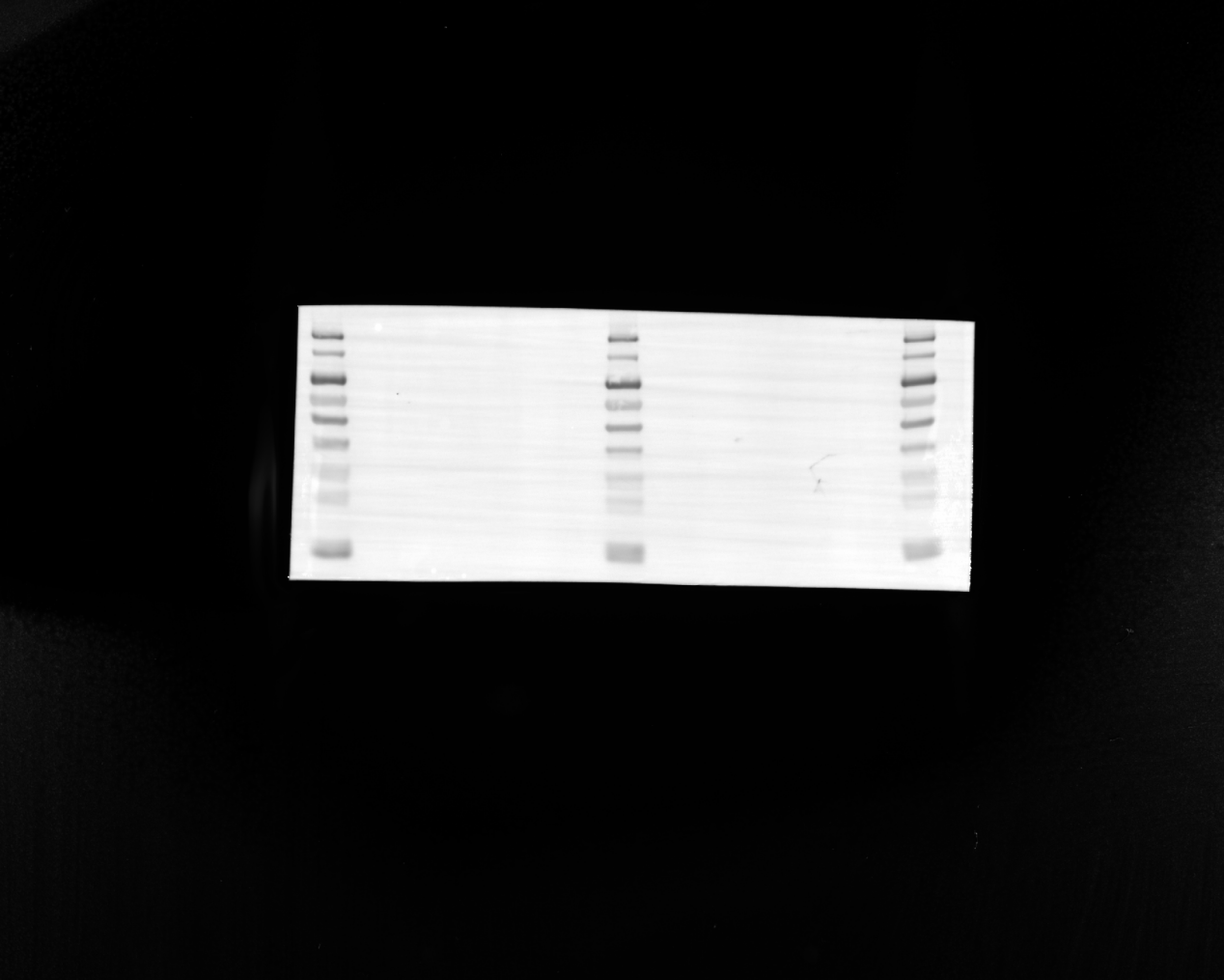

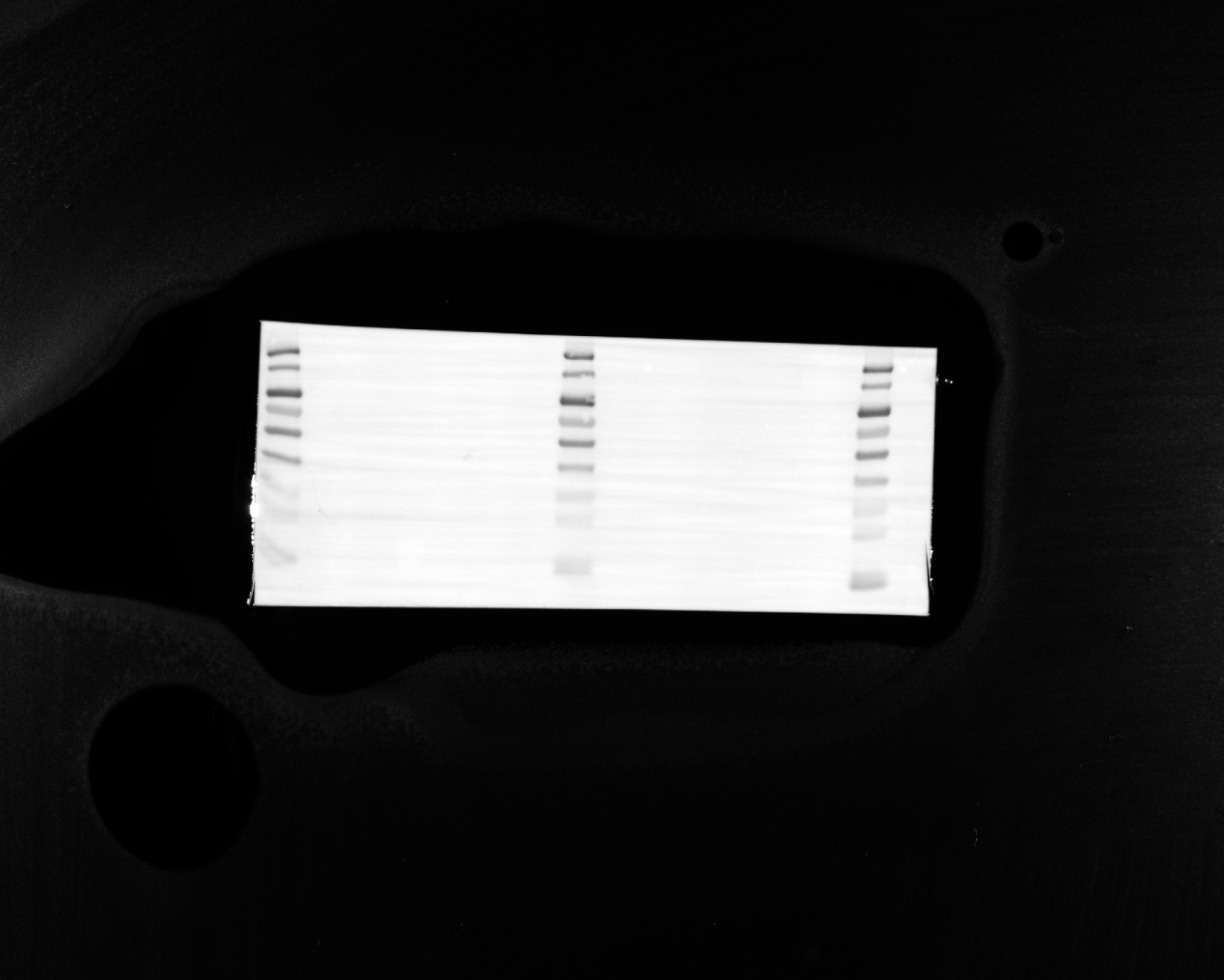

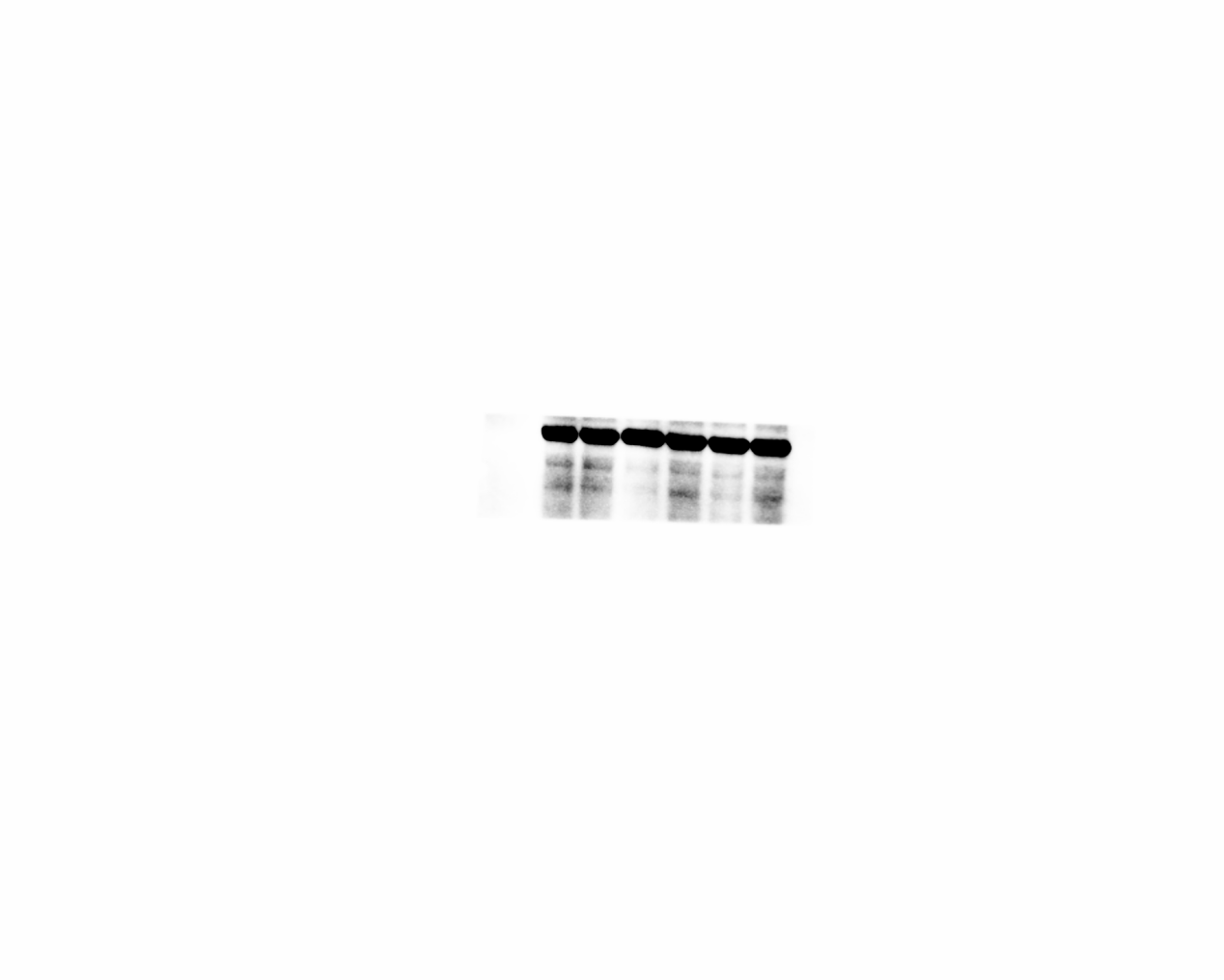


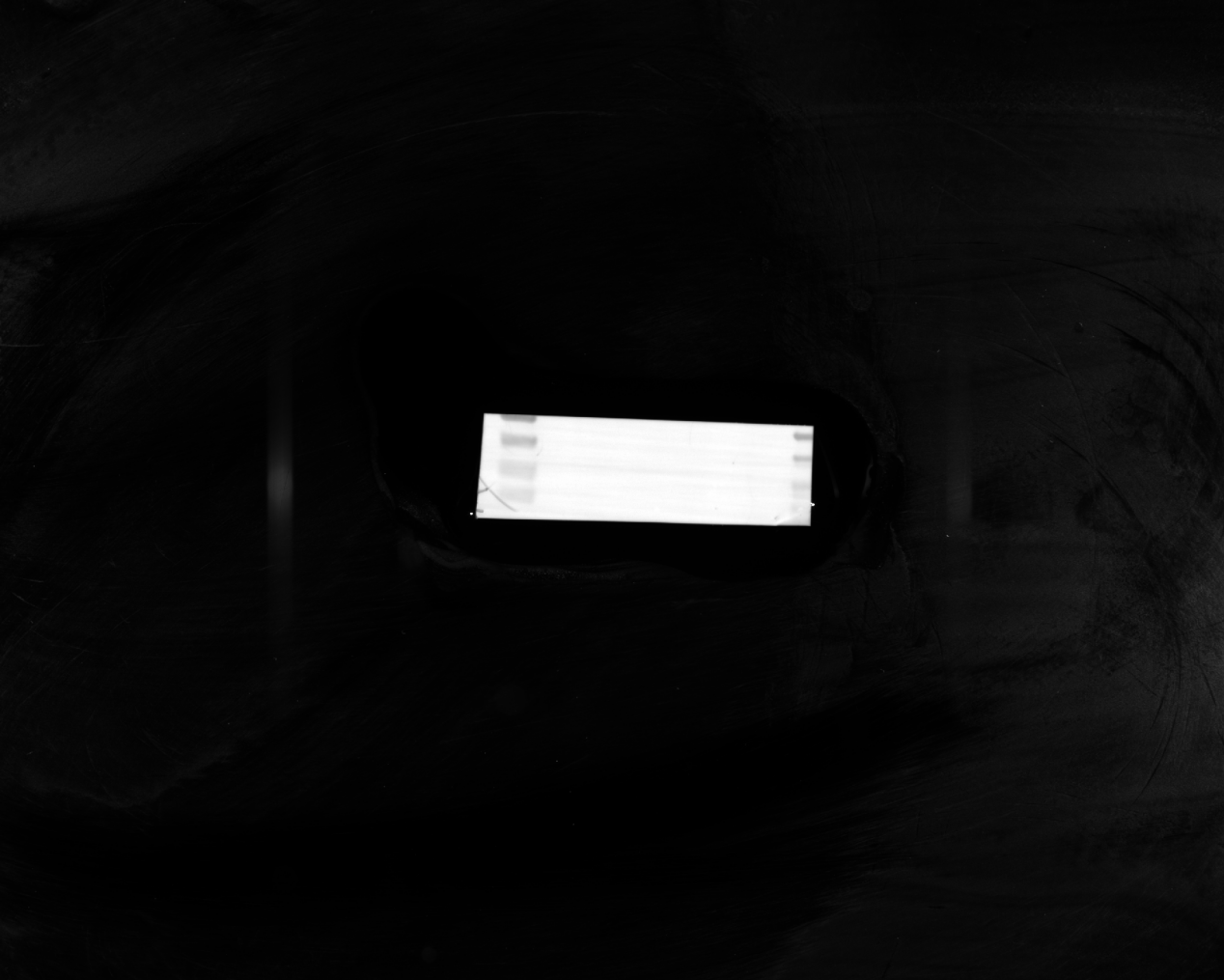


Β-actin


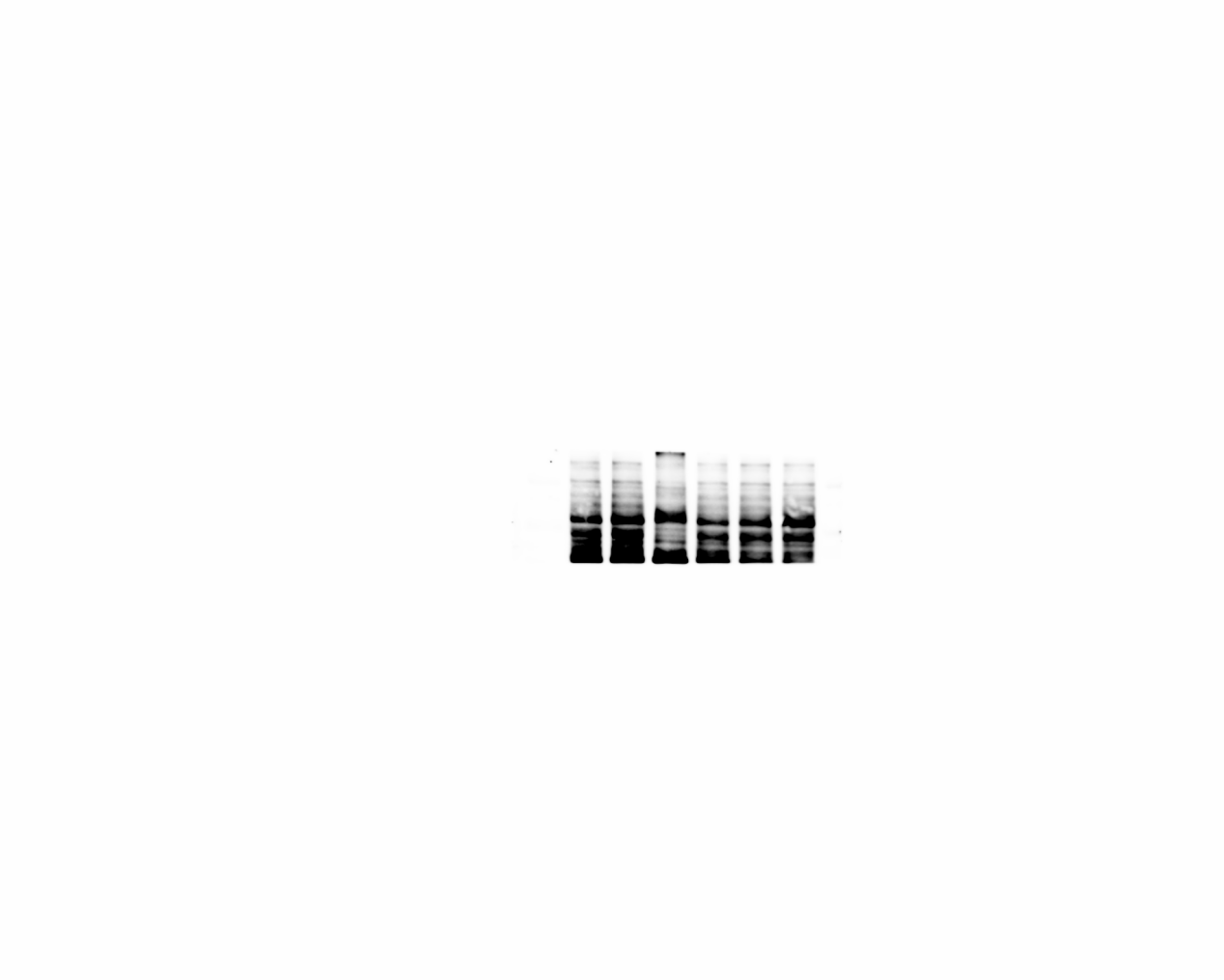

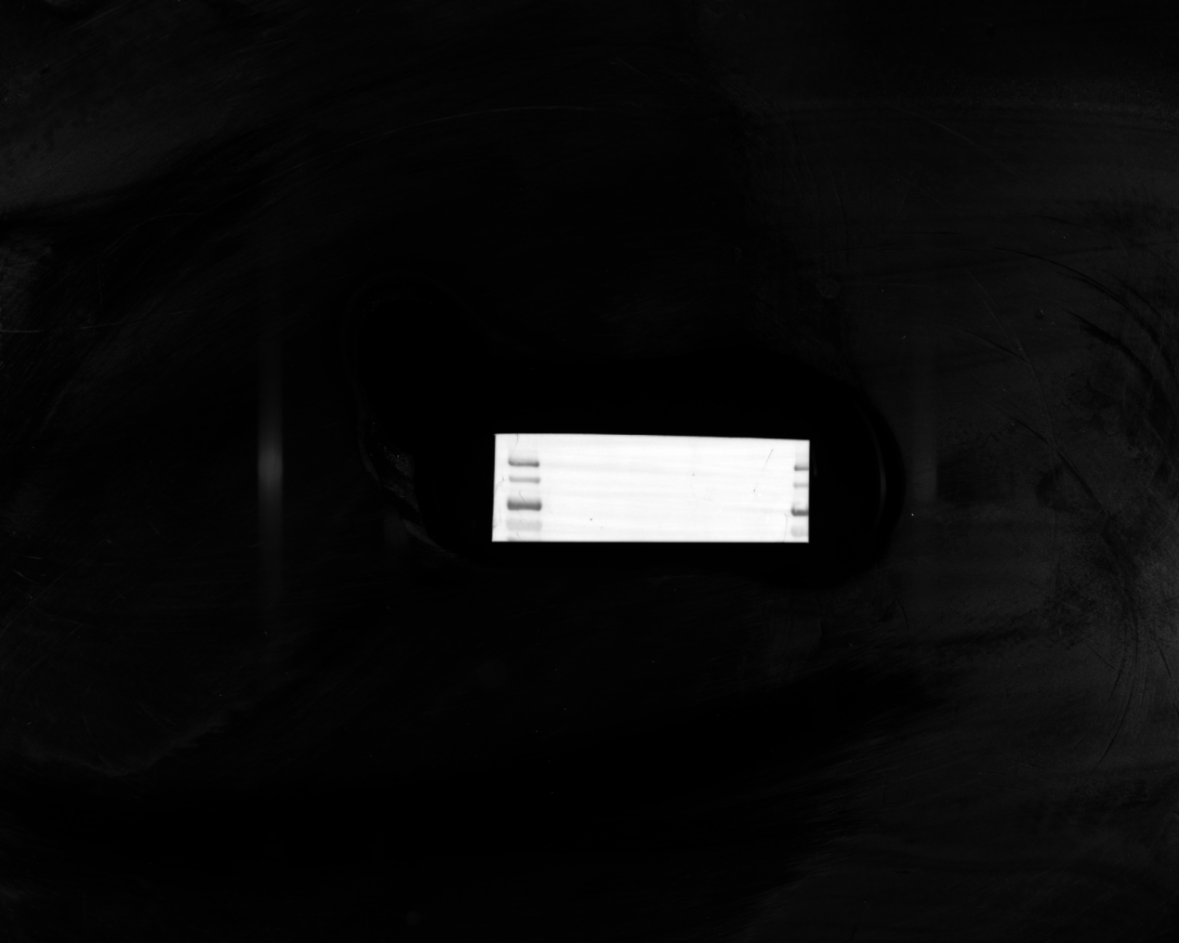


NRF2


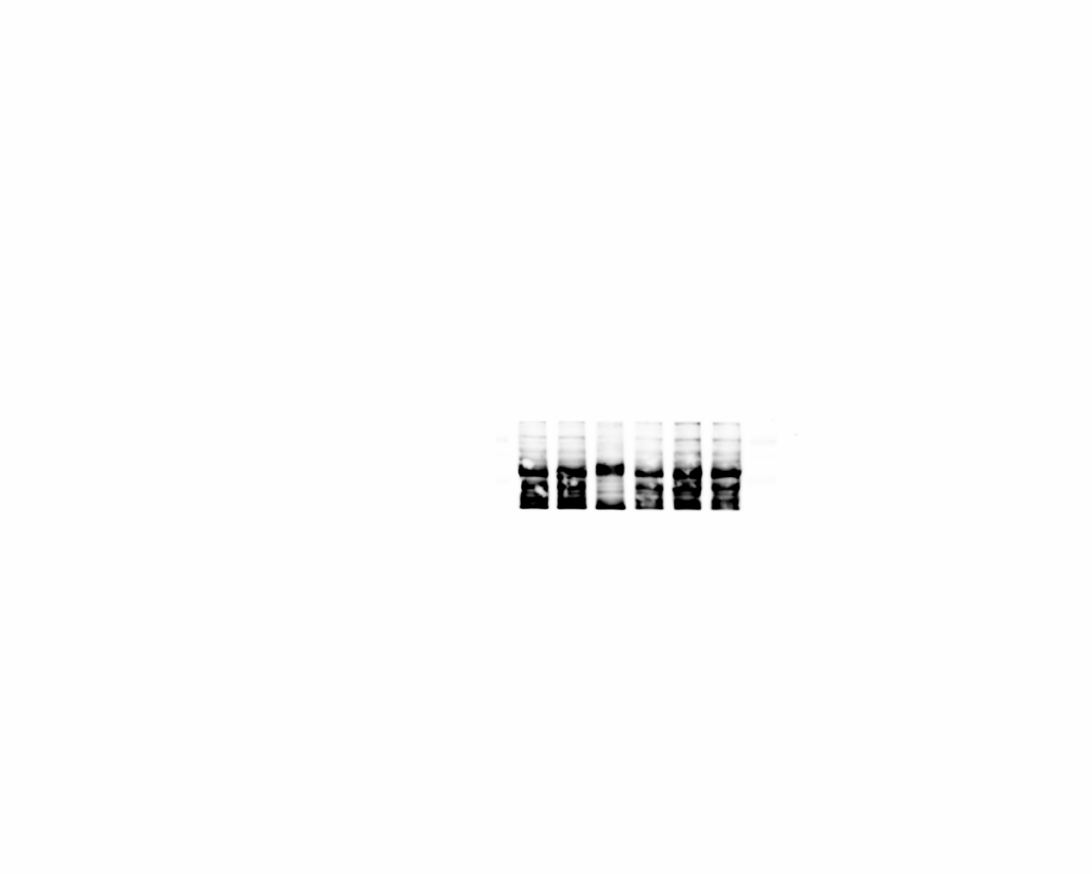

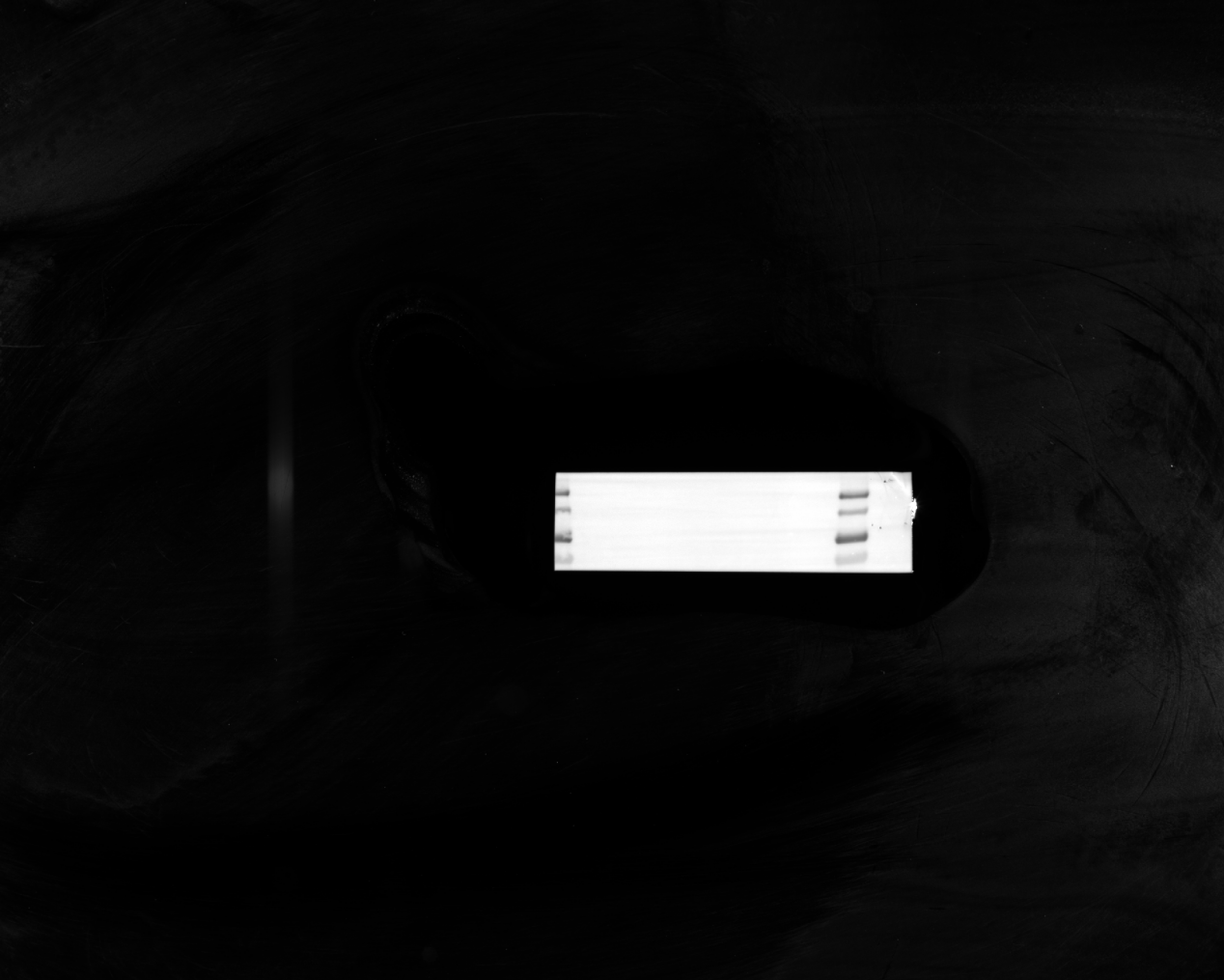


NRF2


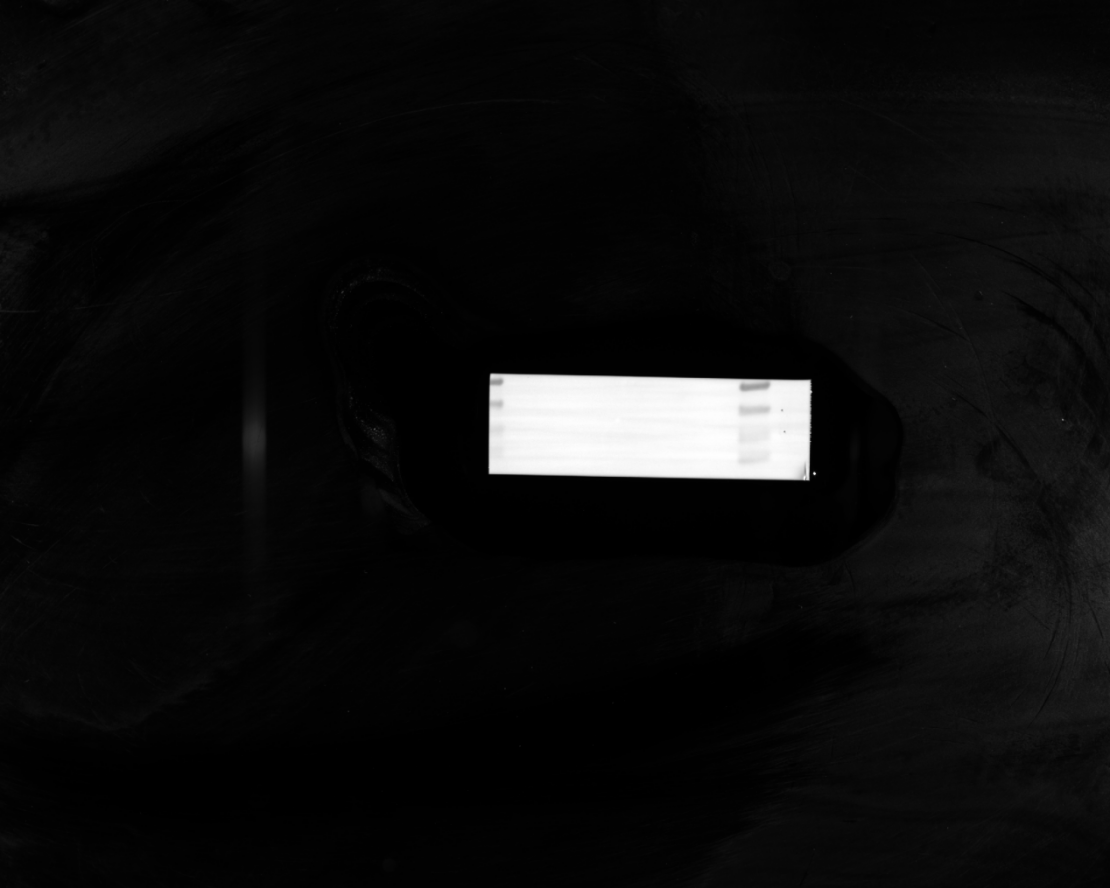

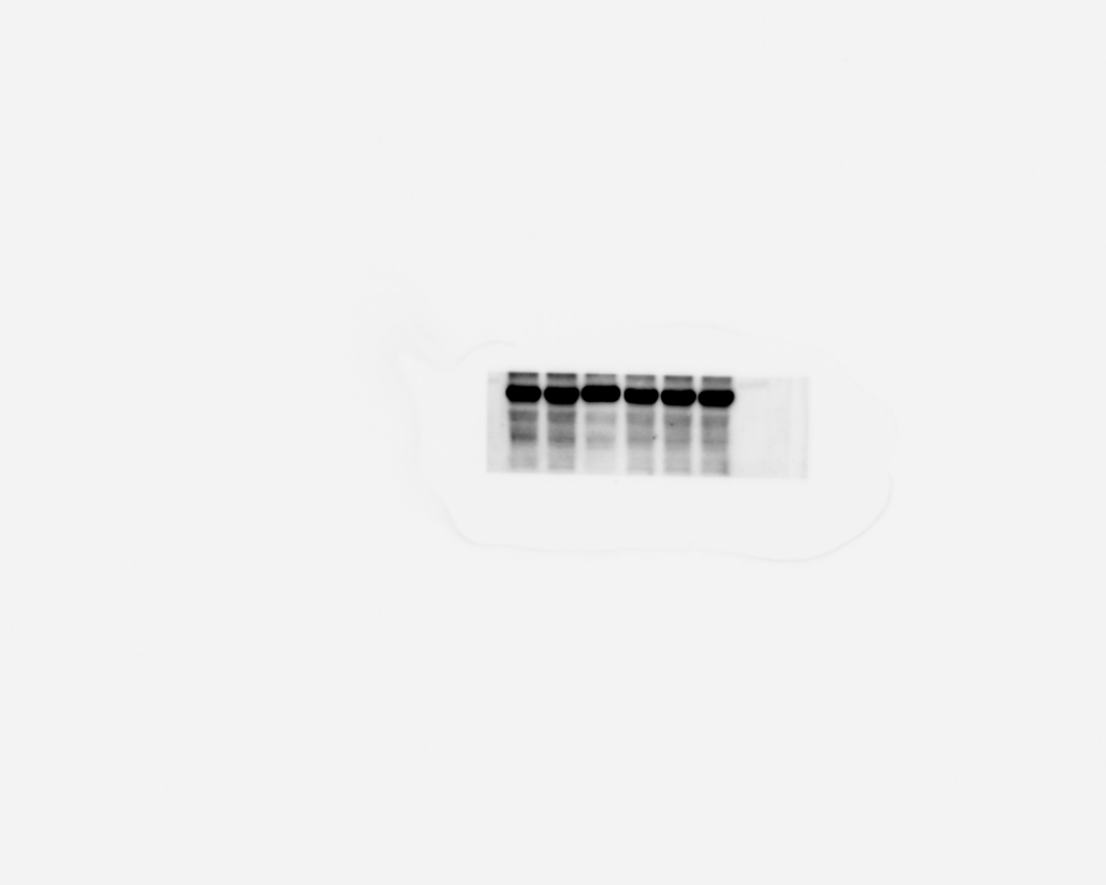


Β-actin


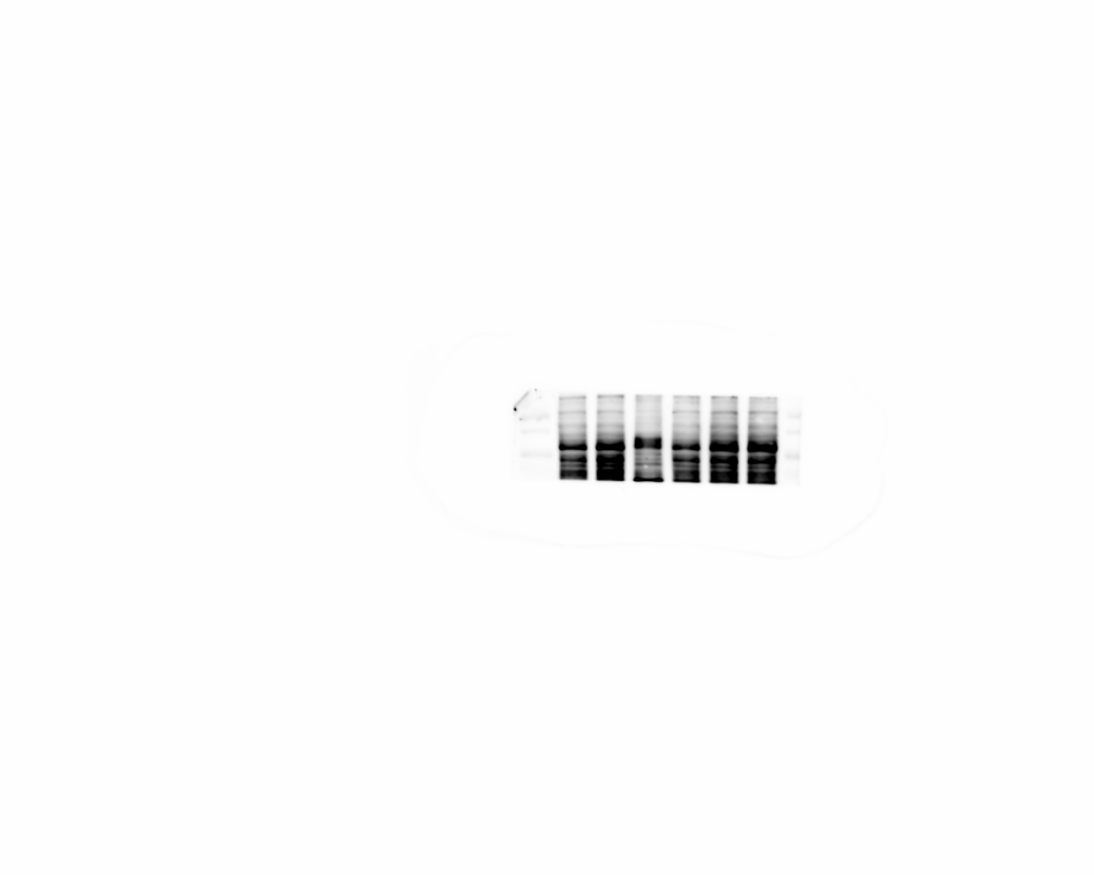

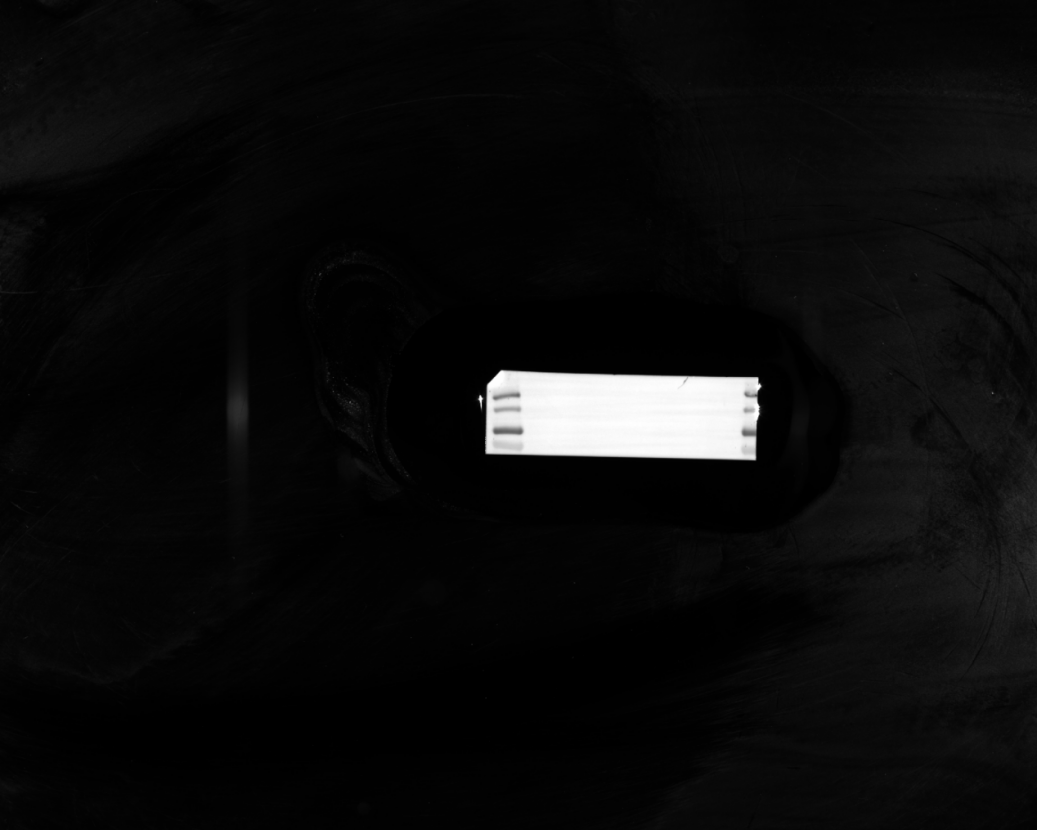


NRF2


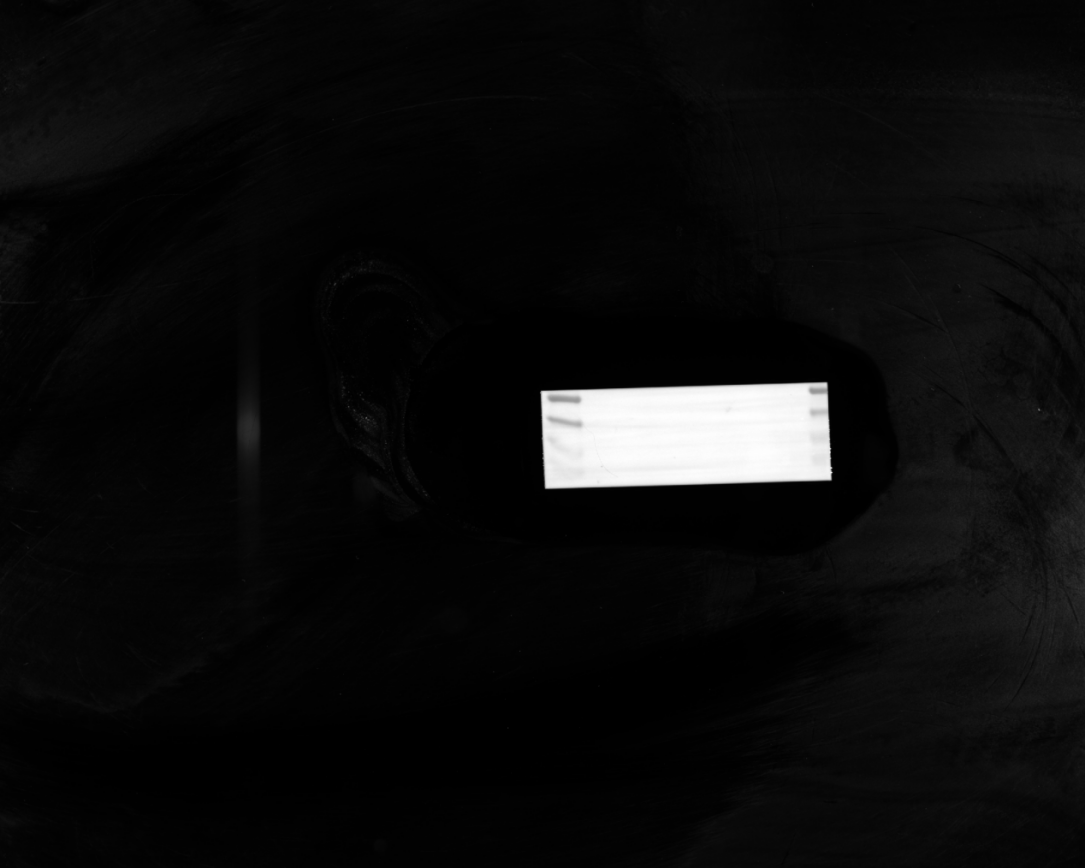

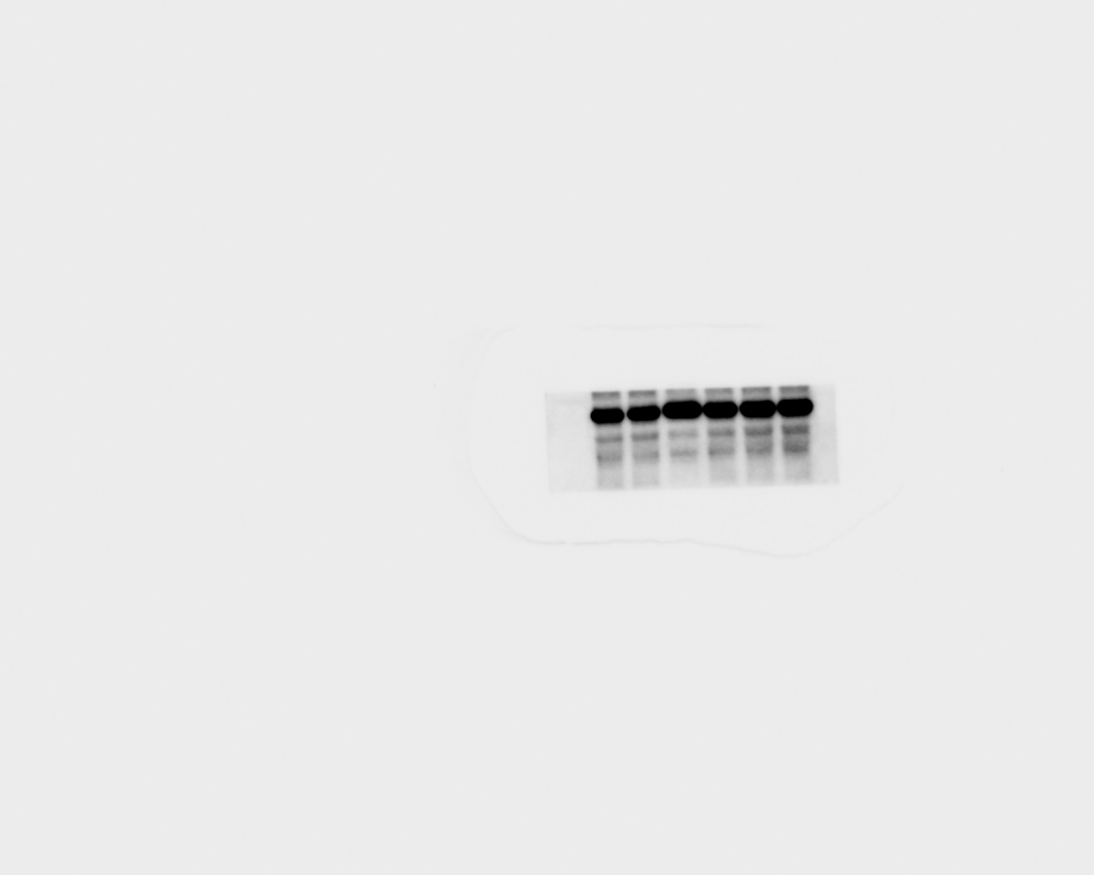


Β-actin

GCLM(whole film)


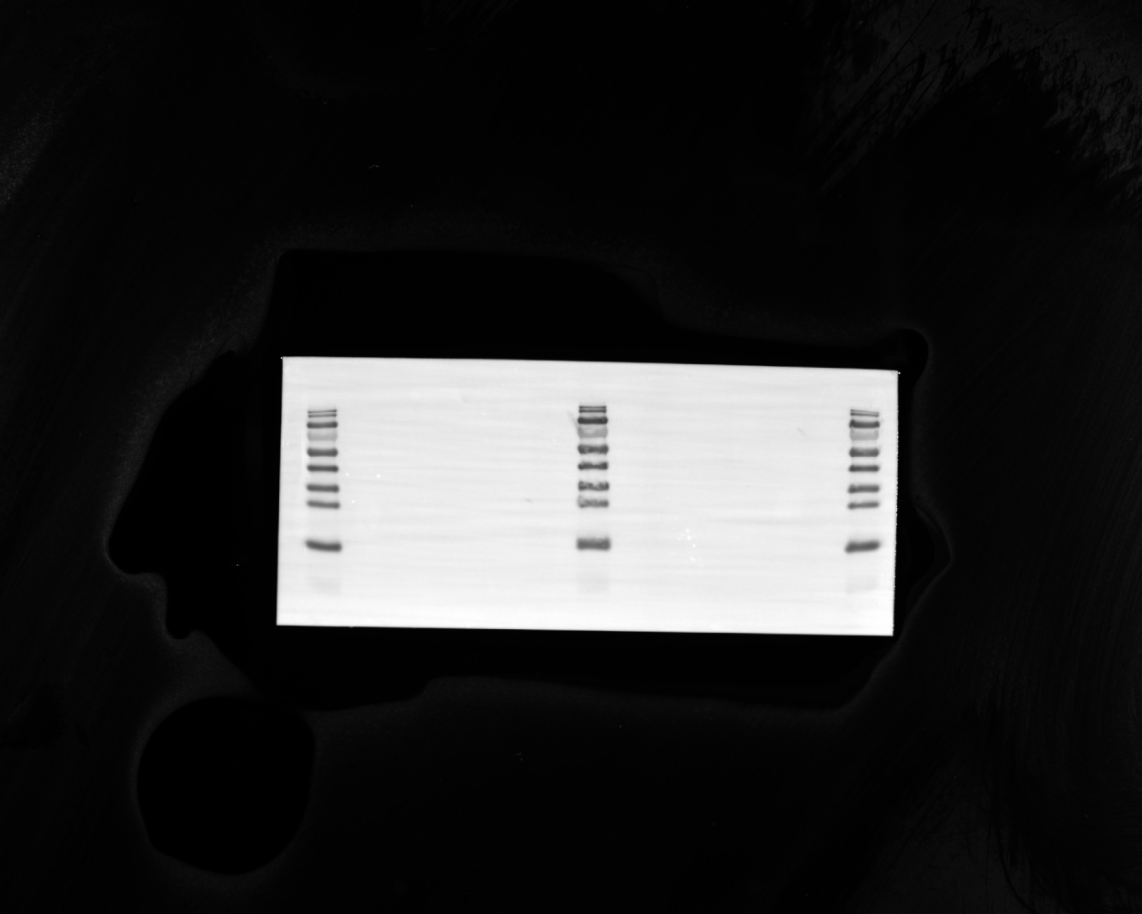

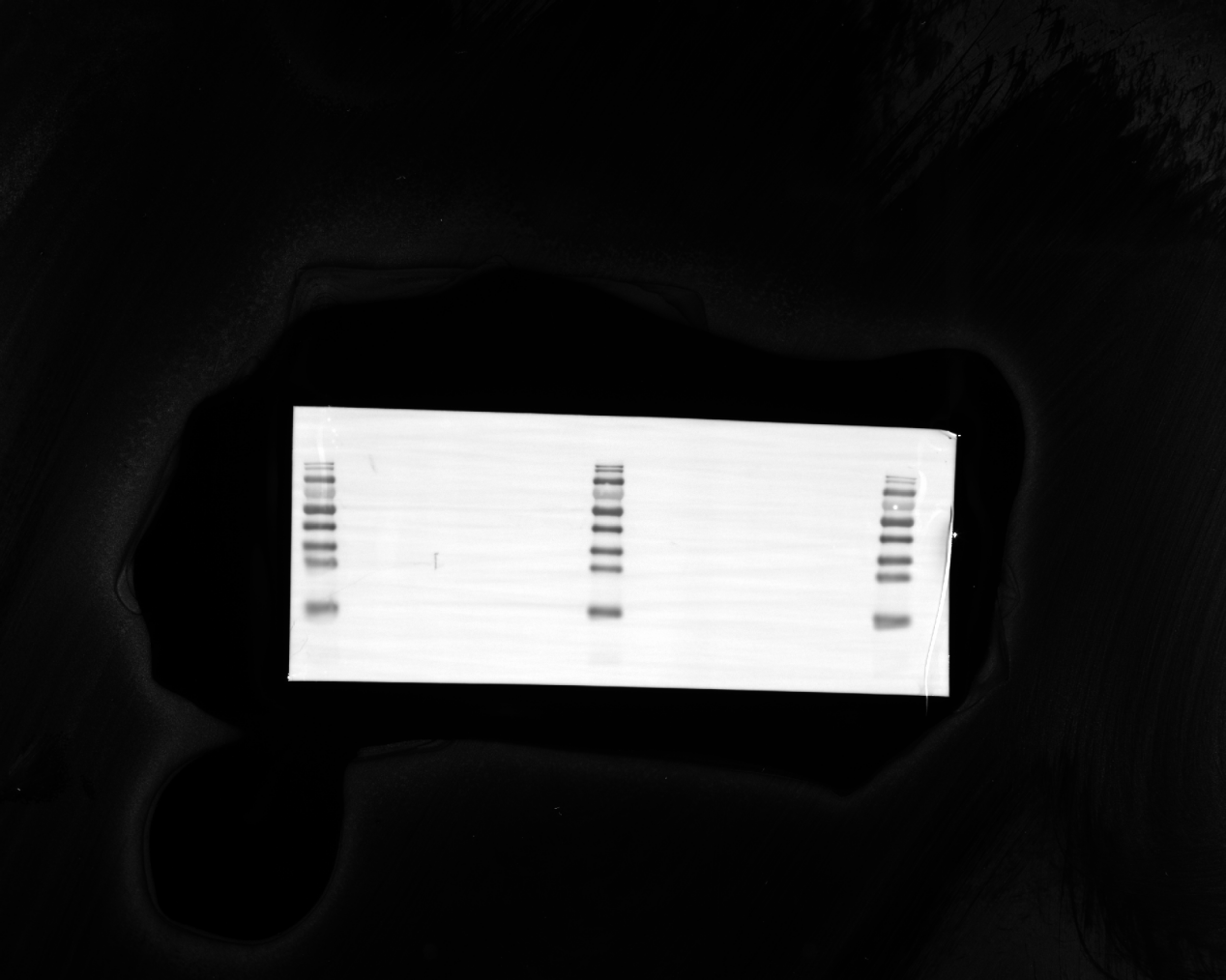


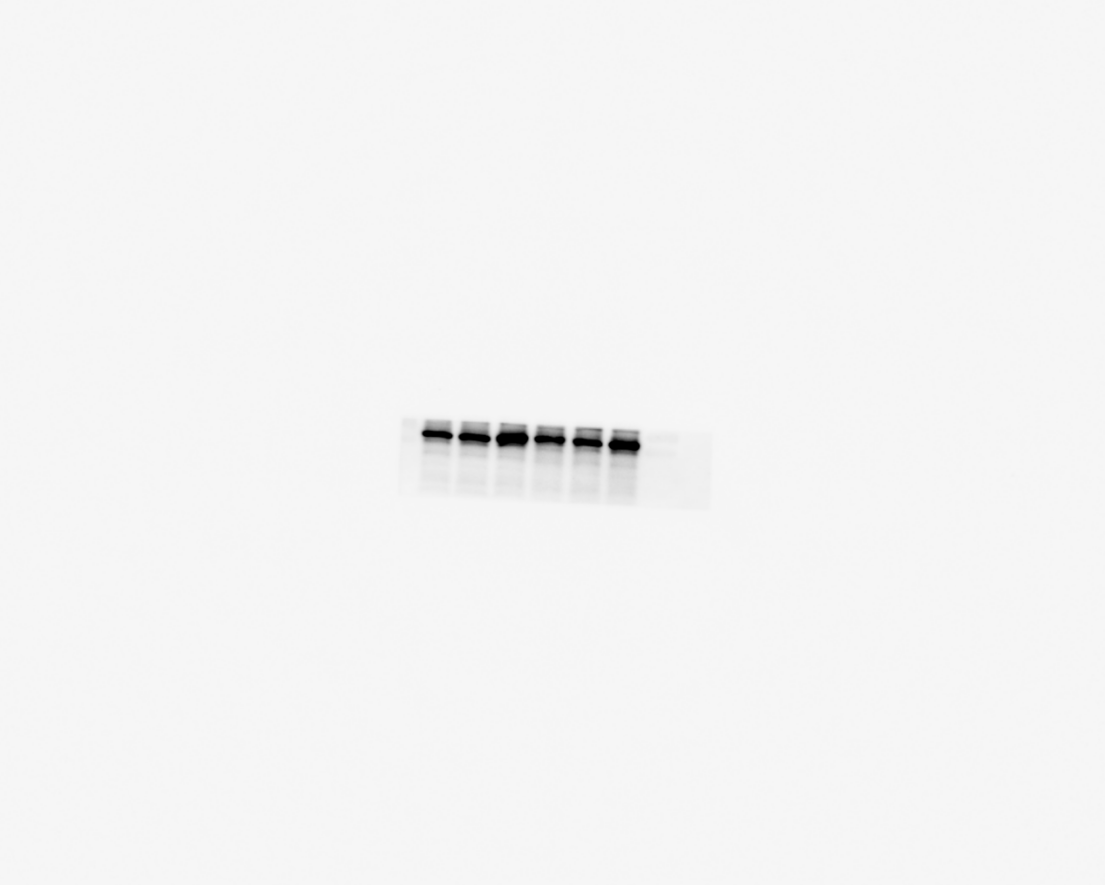

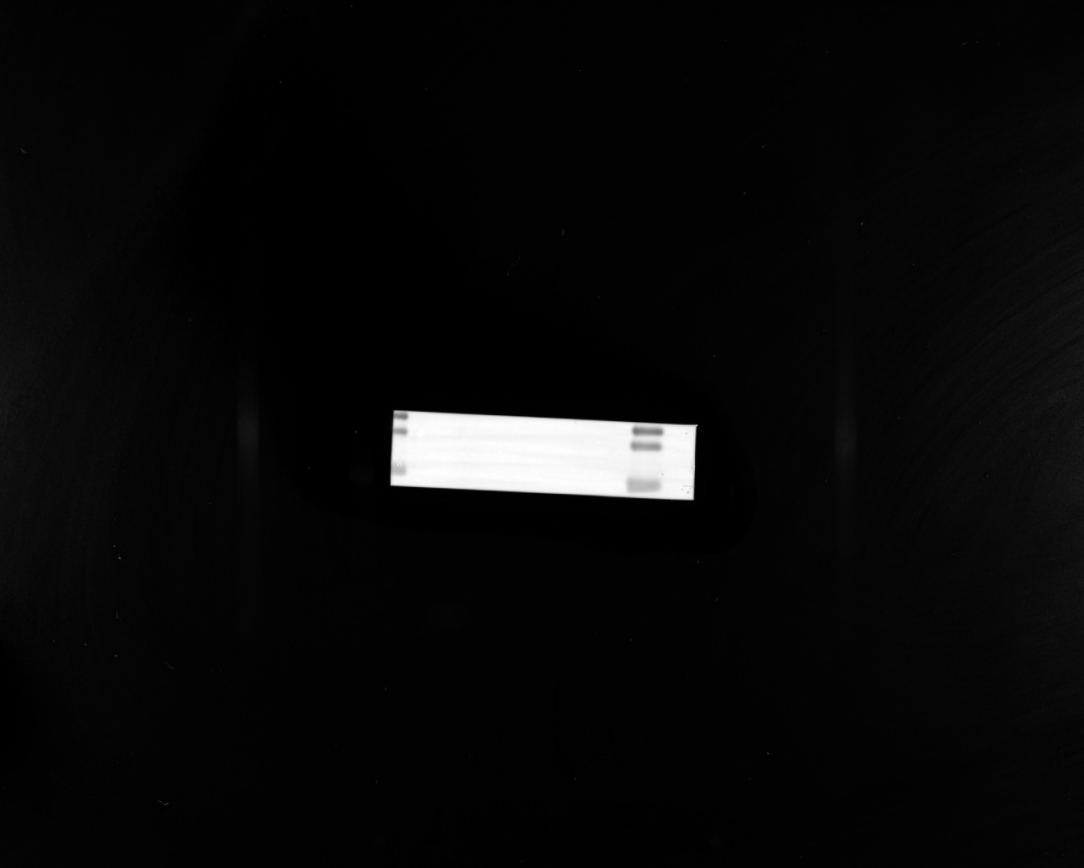


GCLM


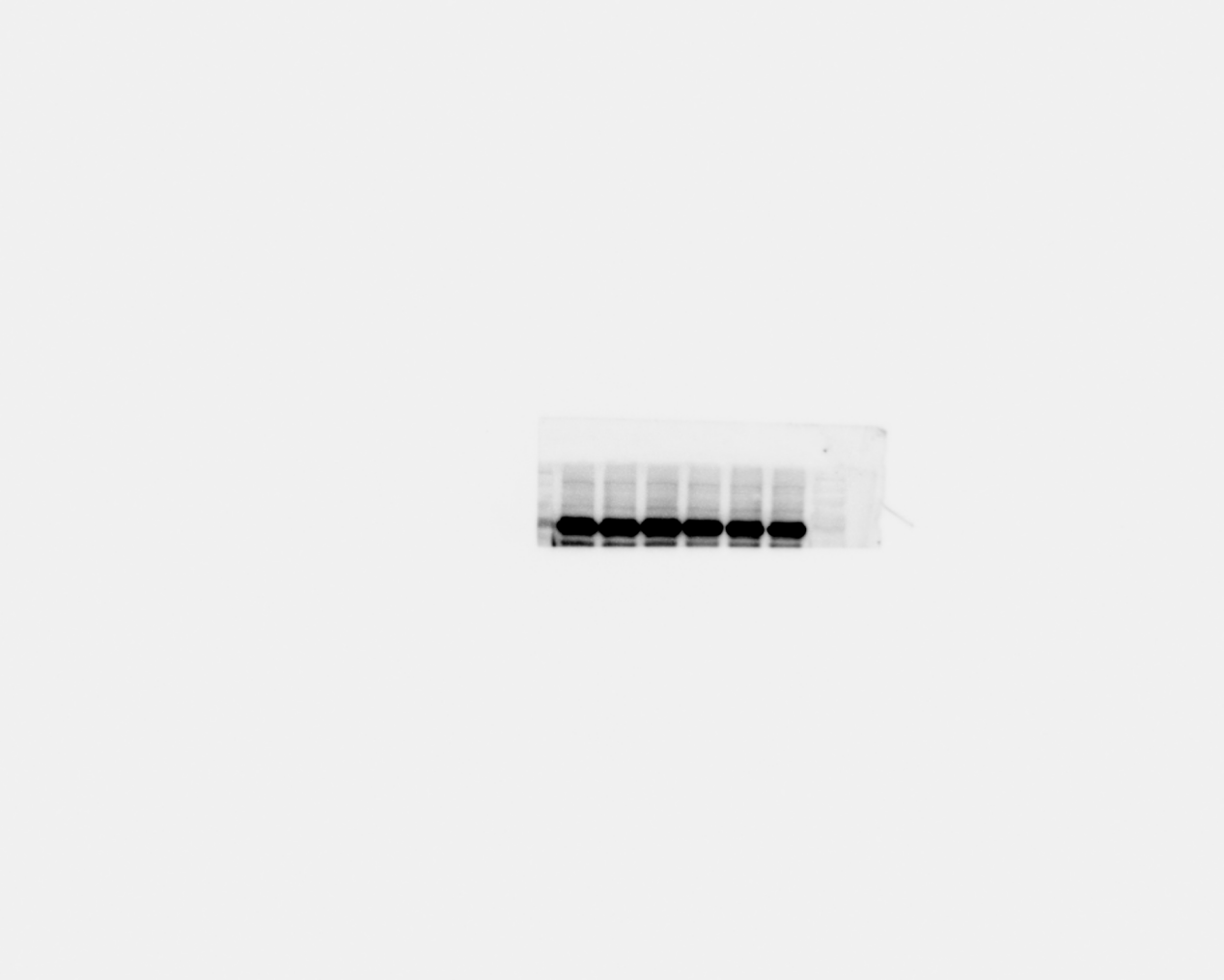

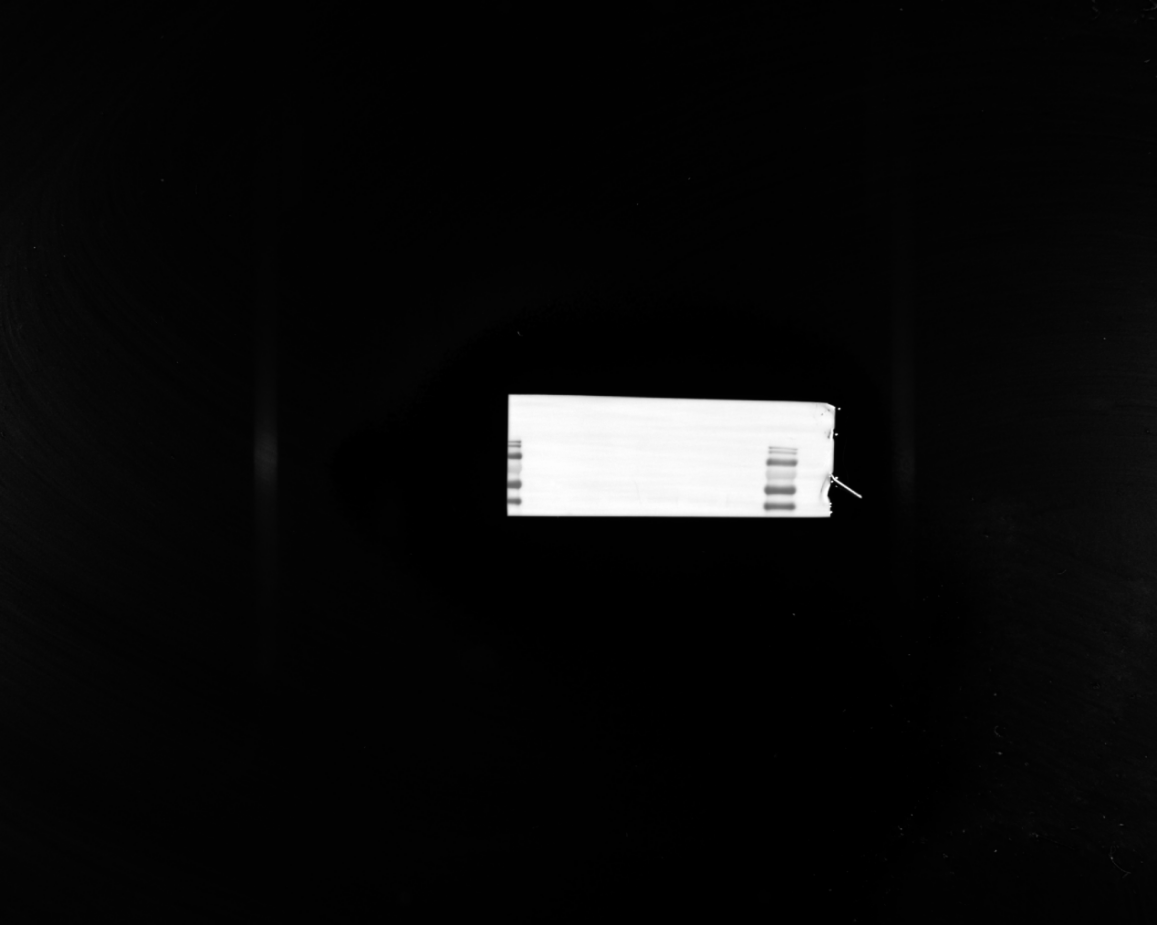


Β-actin


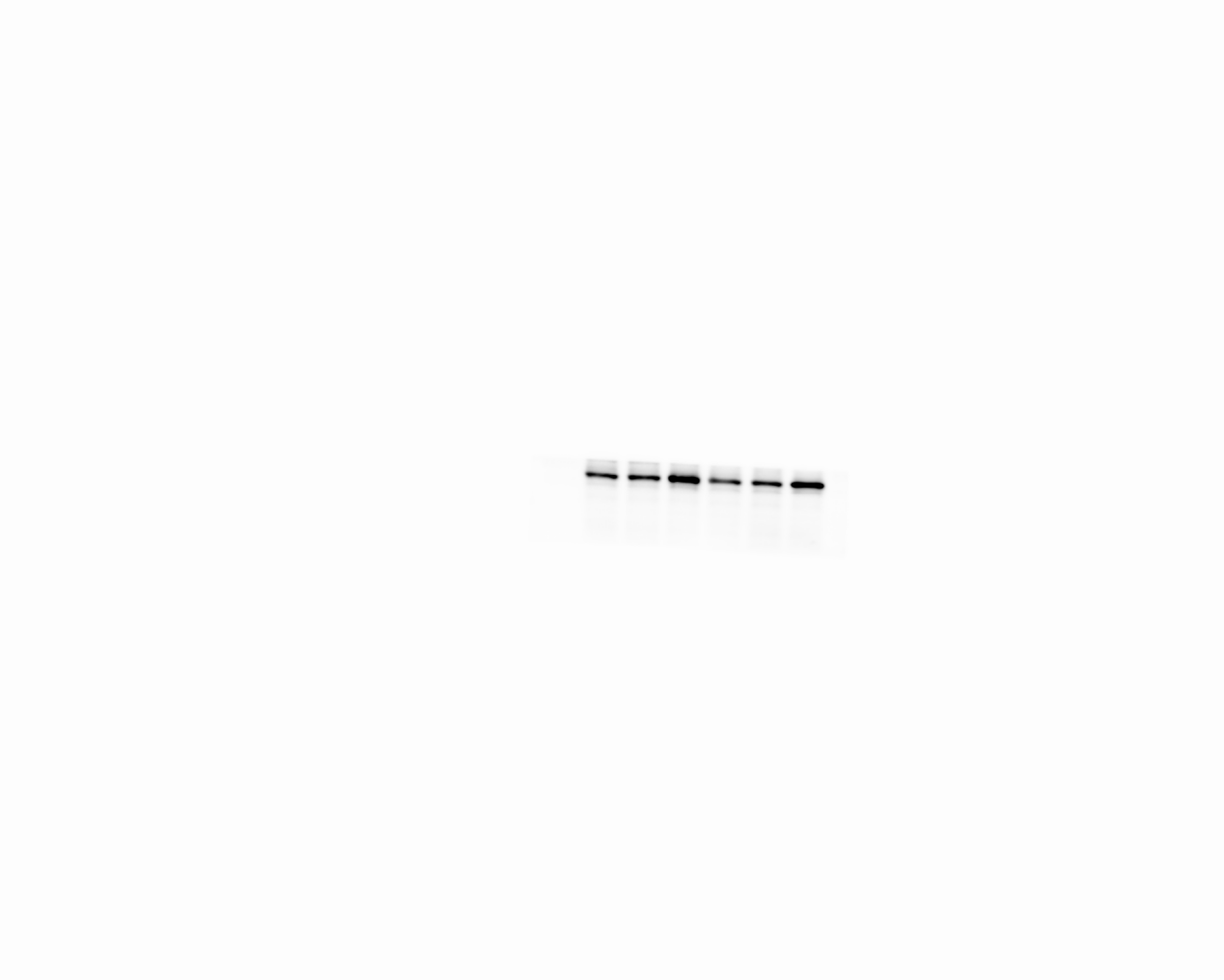

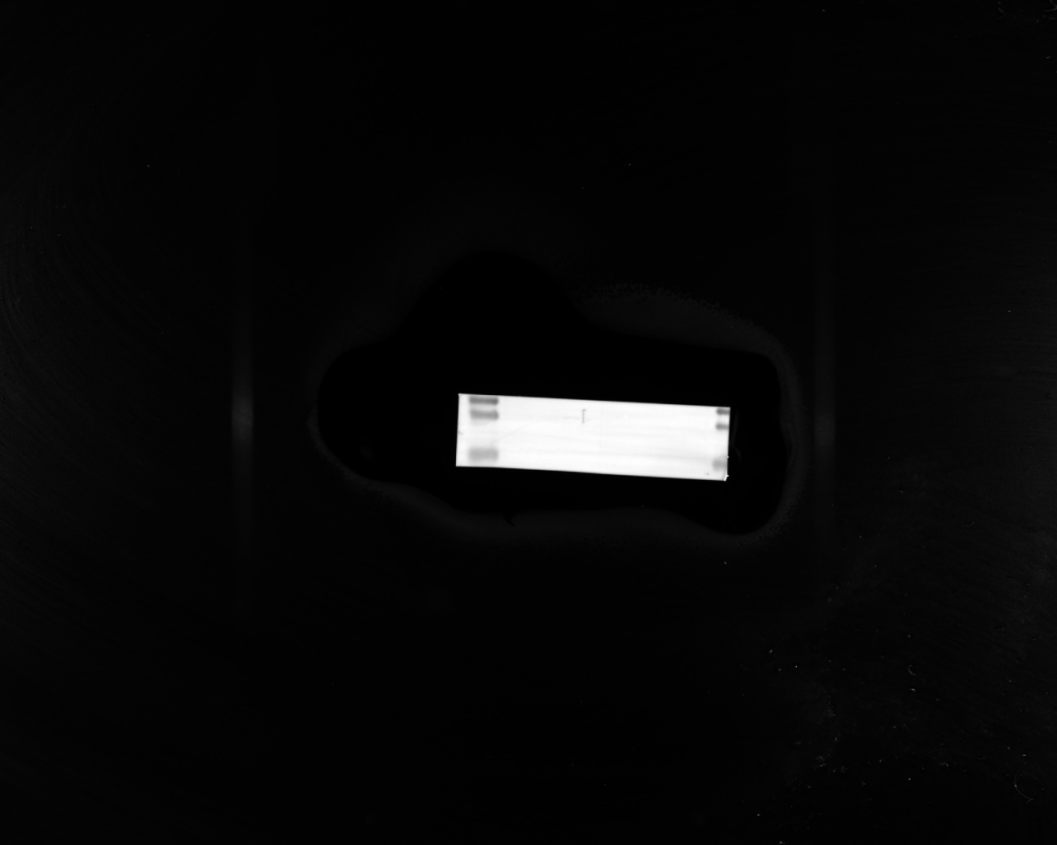


GCLM


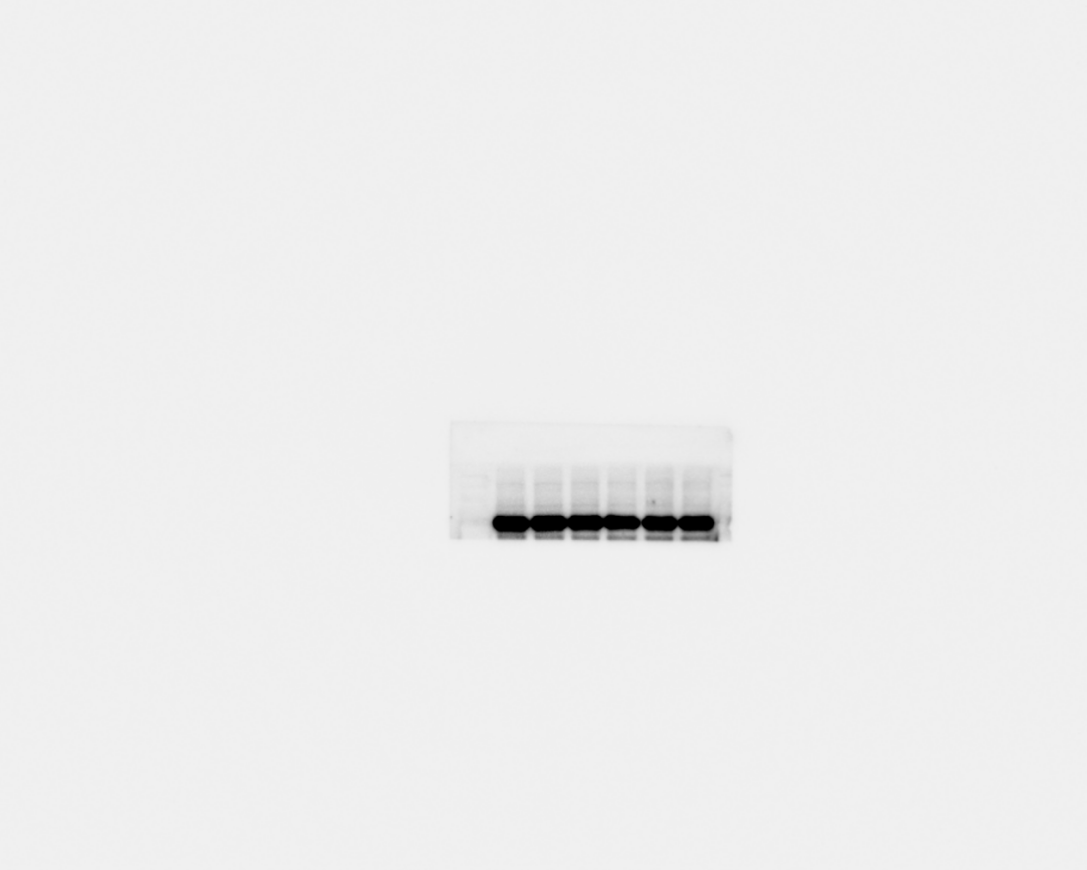

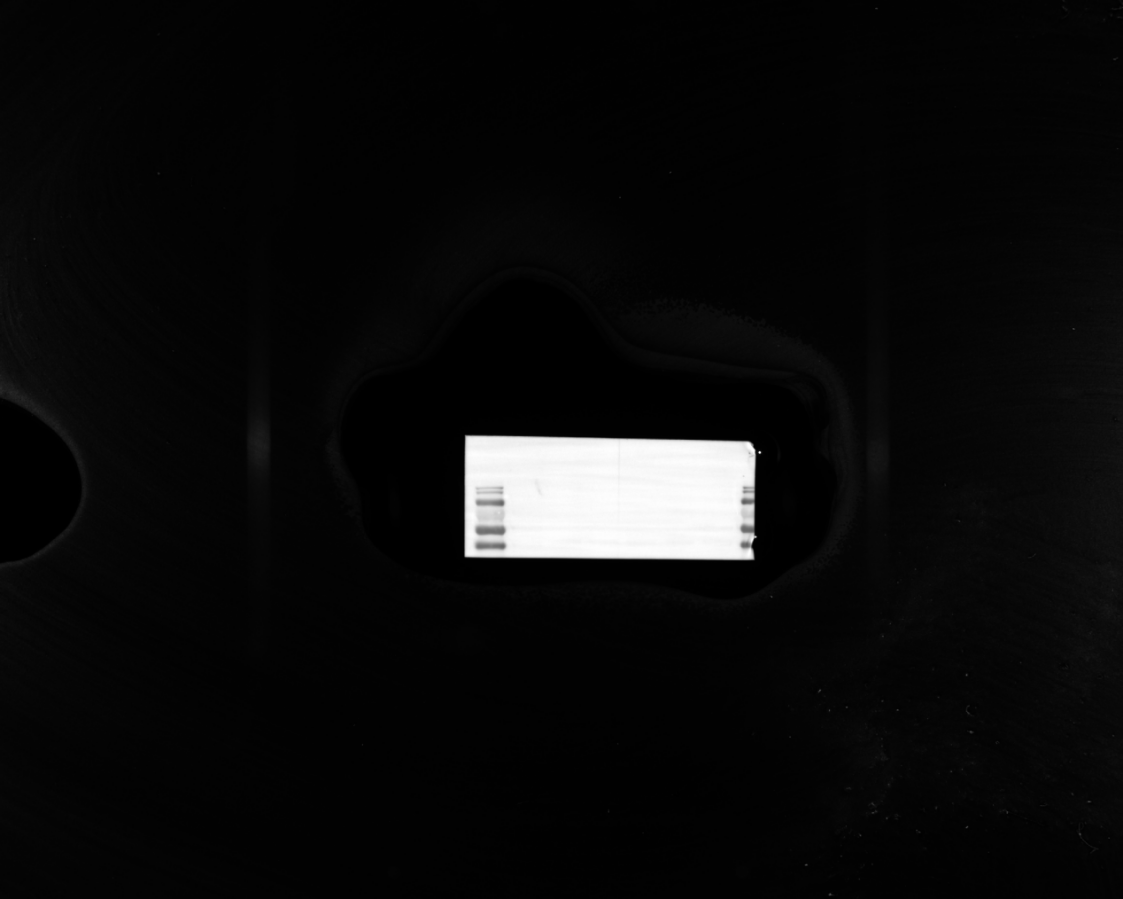


Β-actin


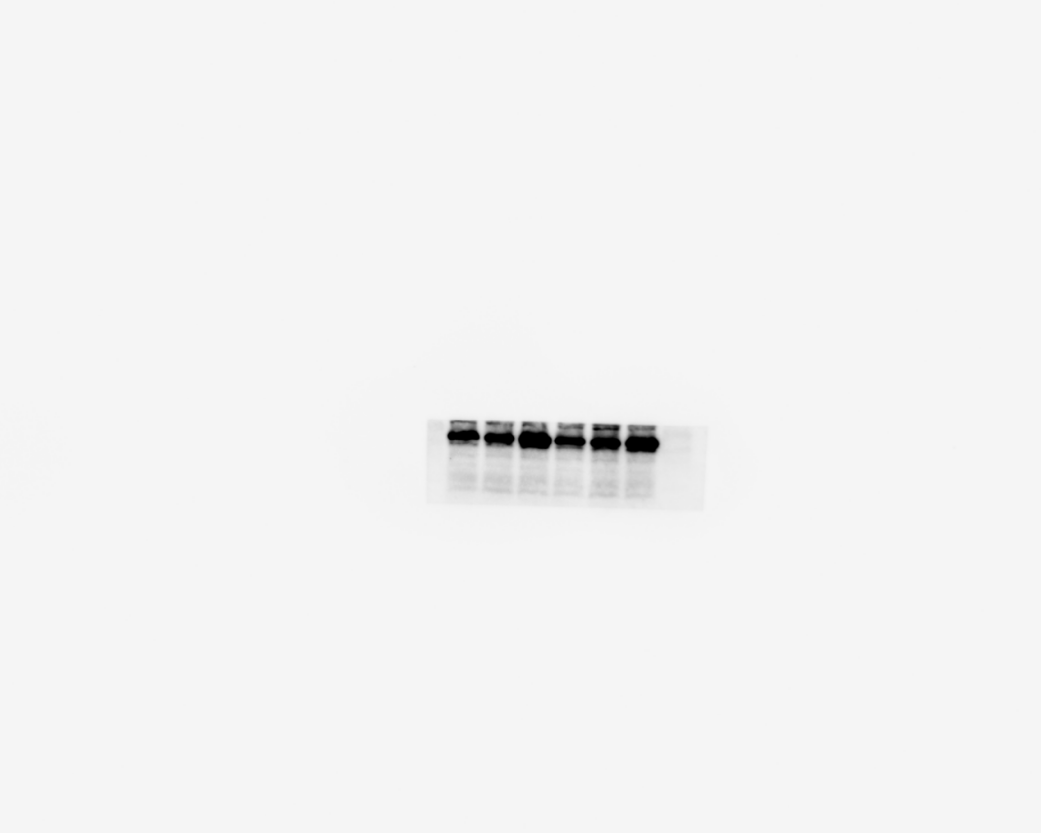

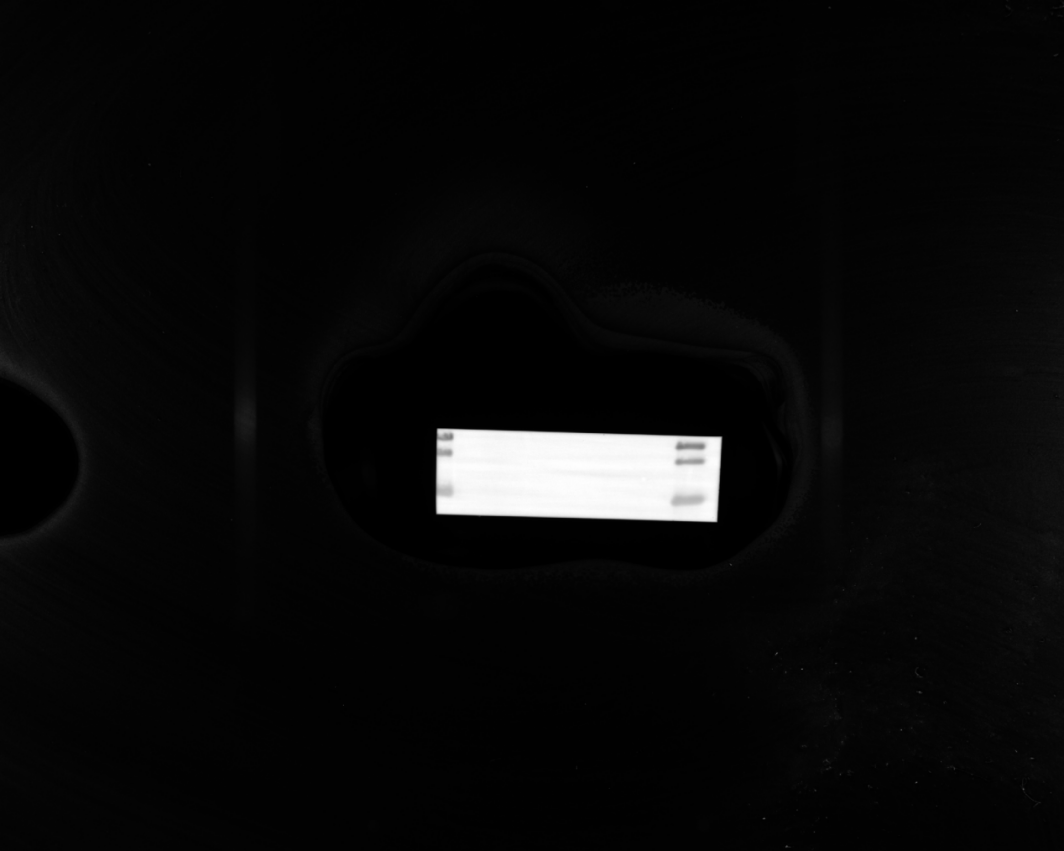


GCLM


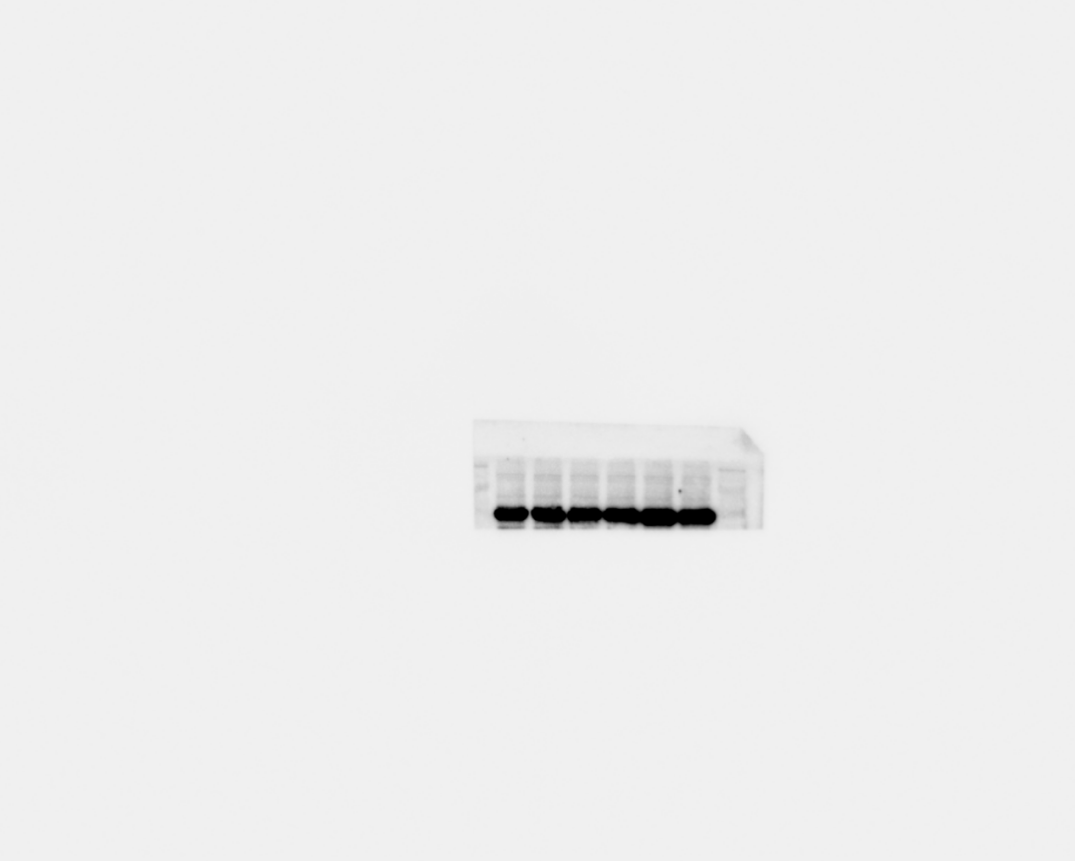

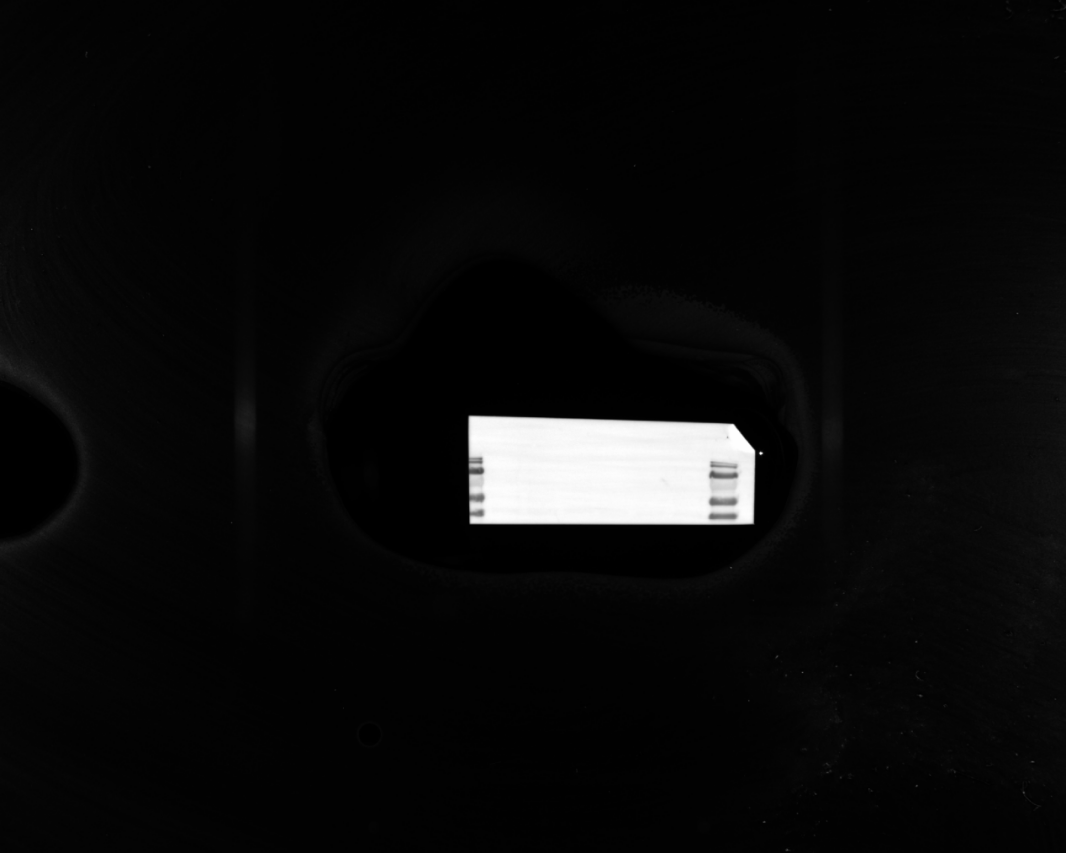


Β-actin


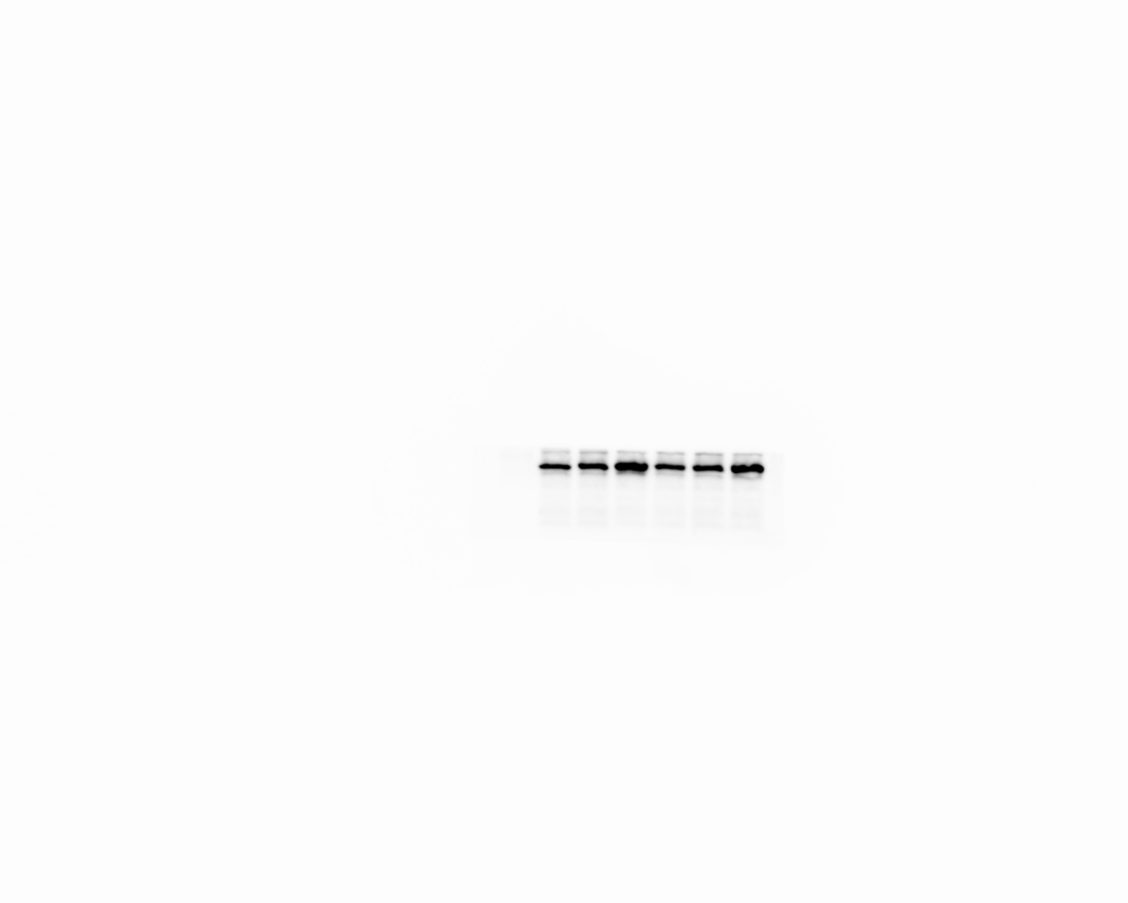

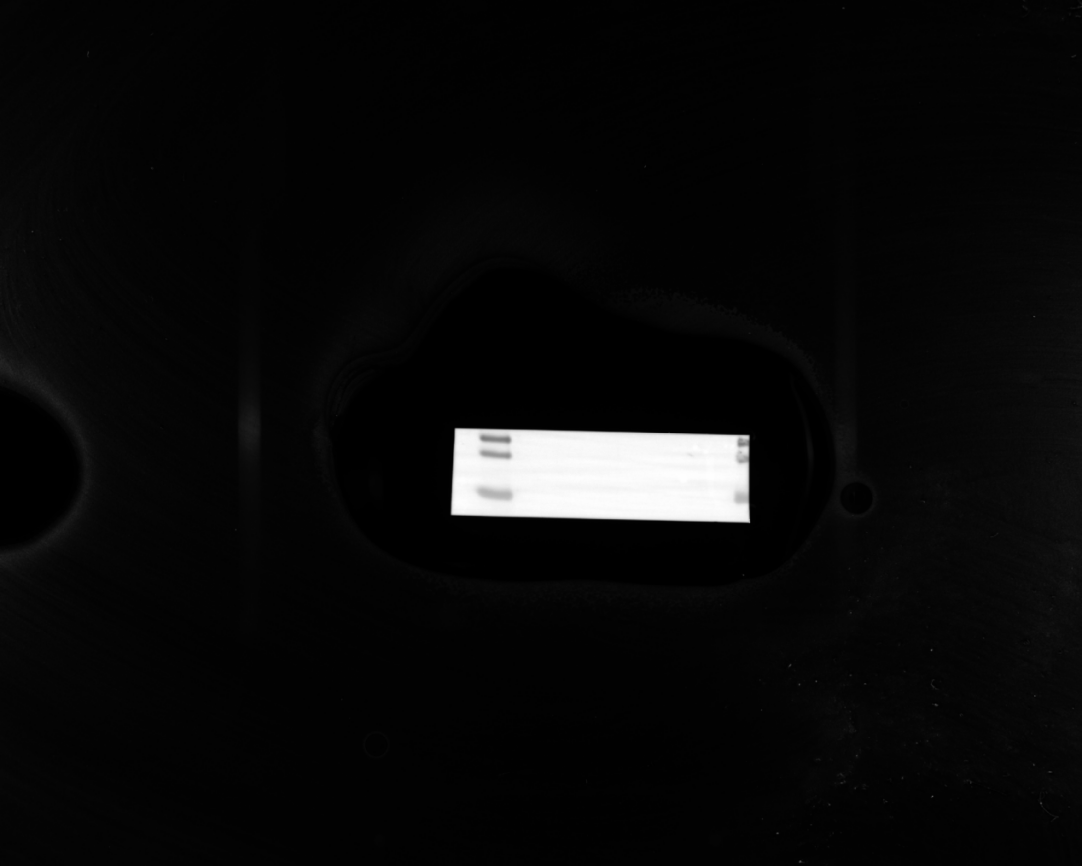


GCLM


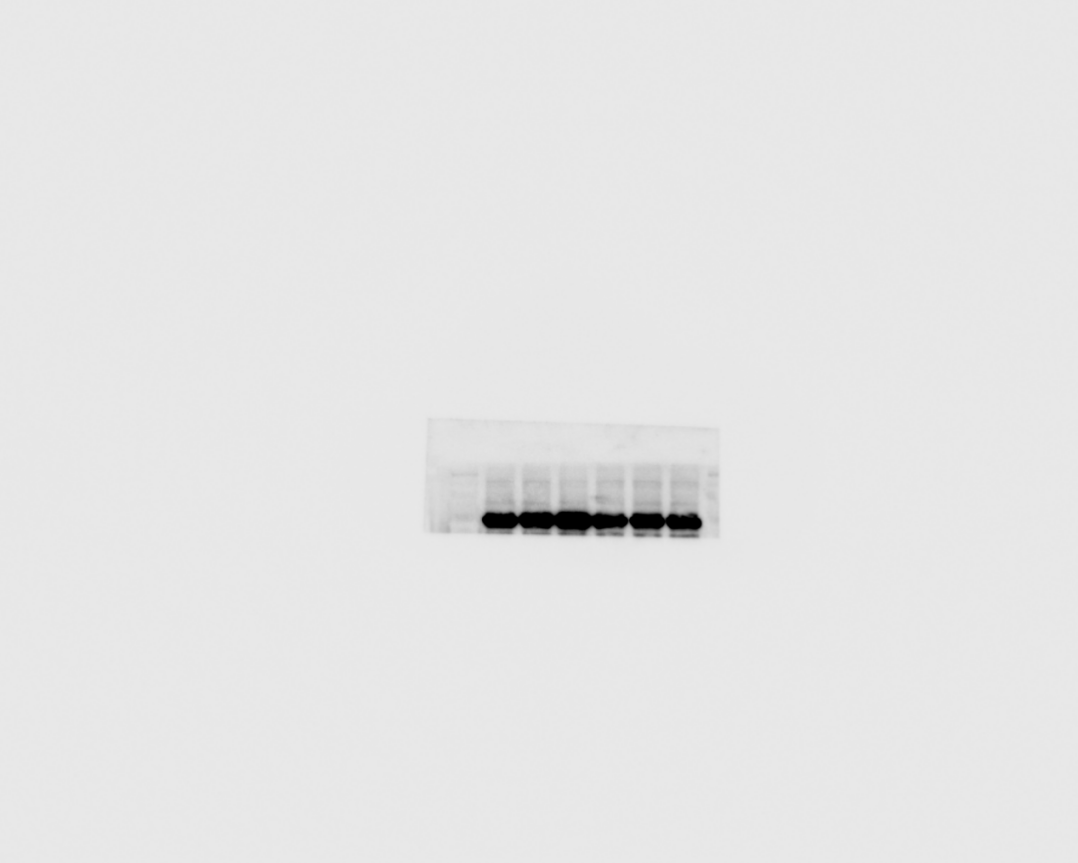

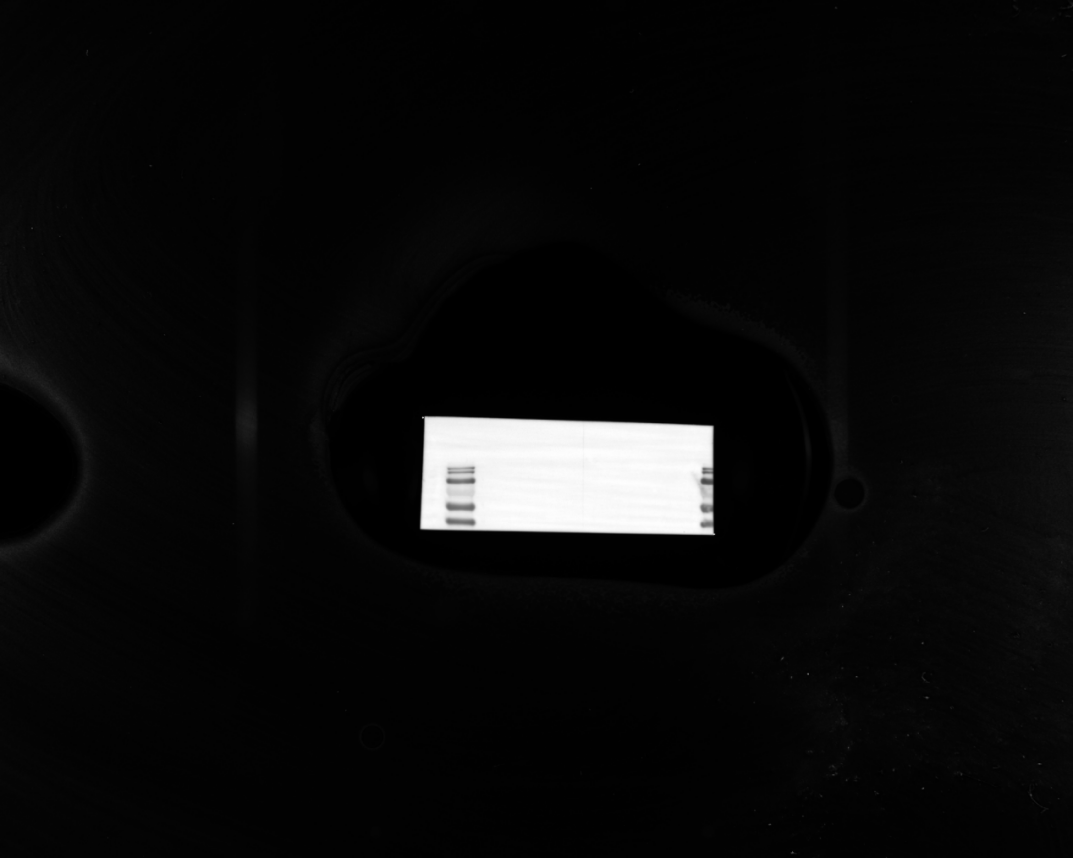


Β-actin

GPX4(whole film)


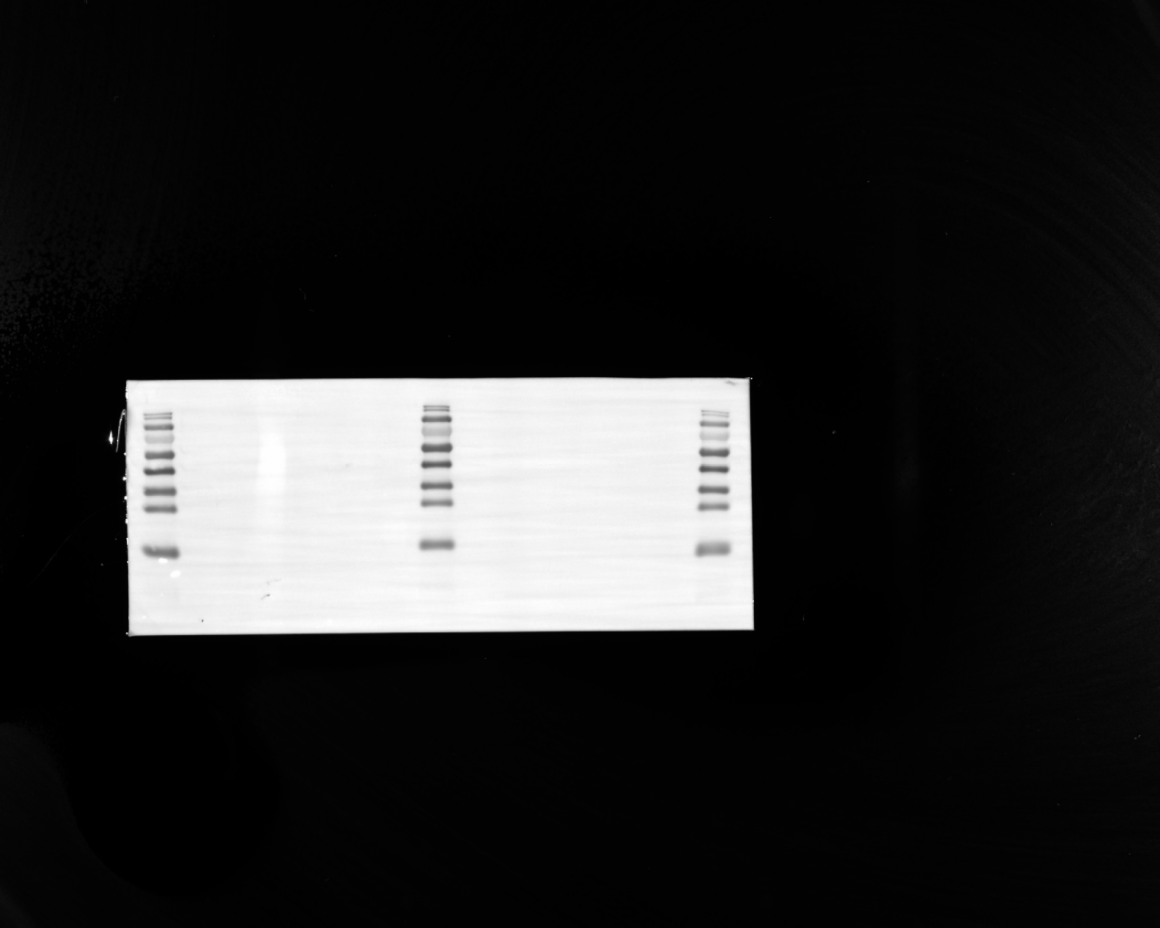

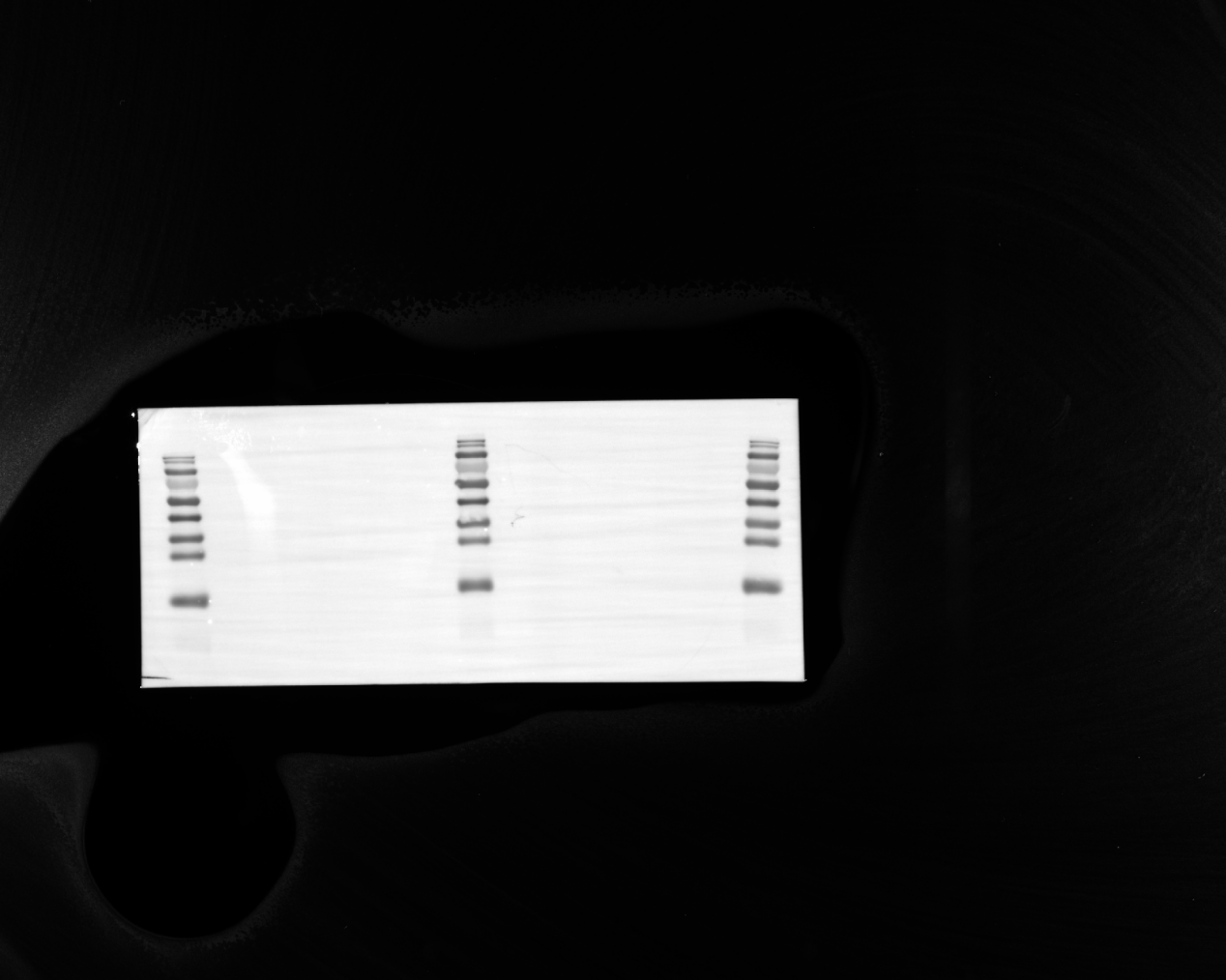

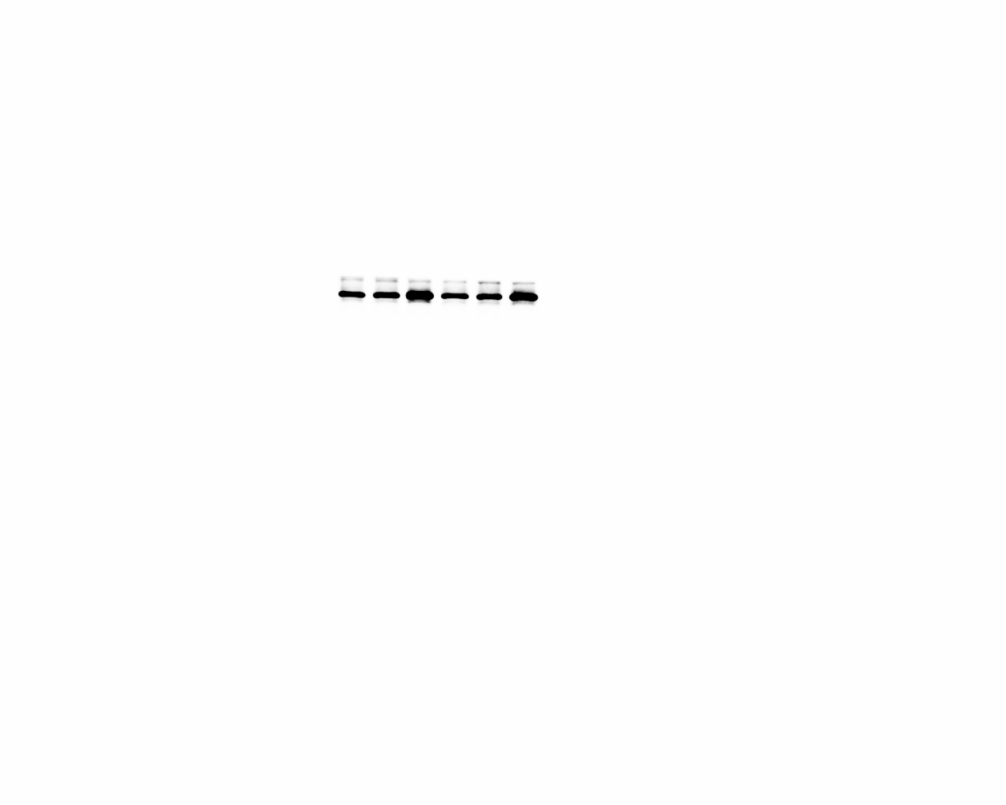

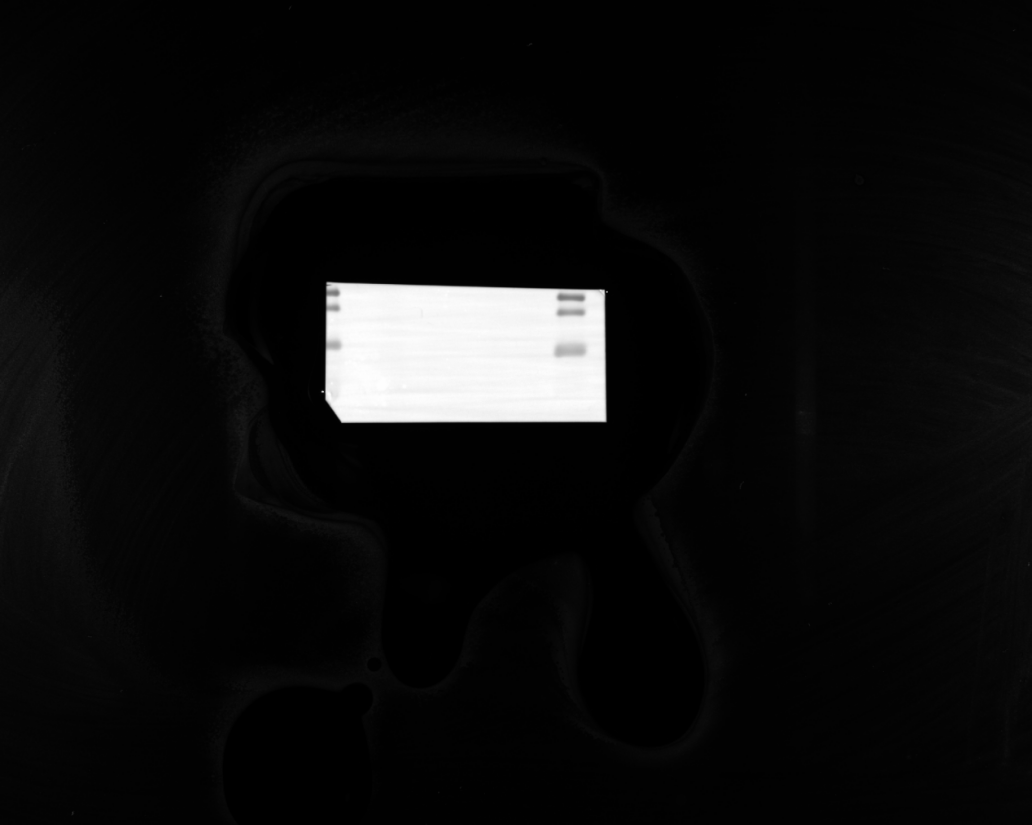


GPX4


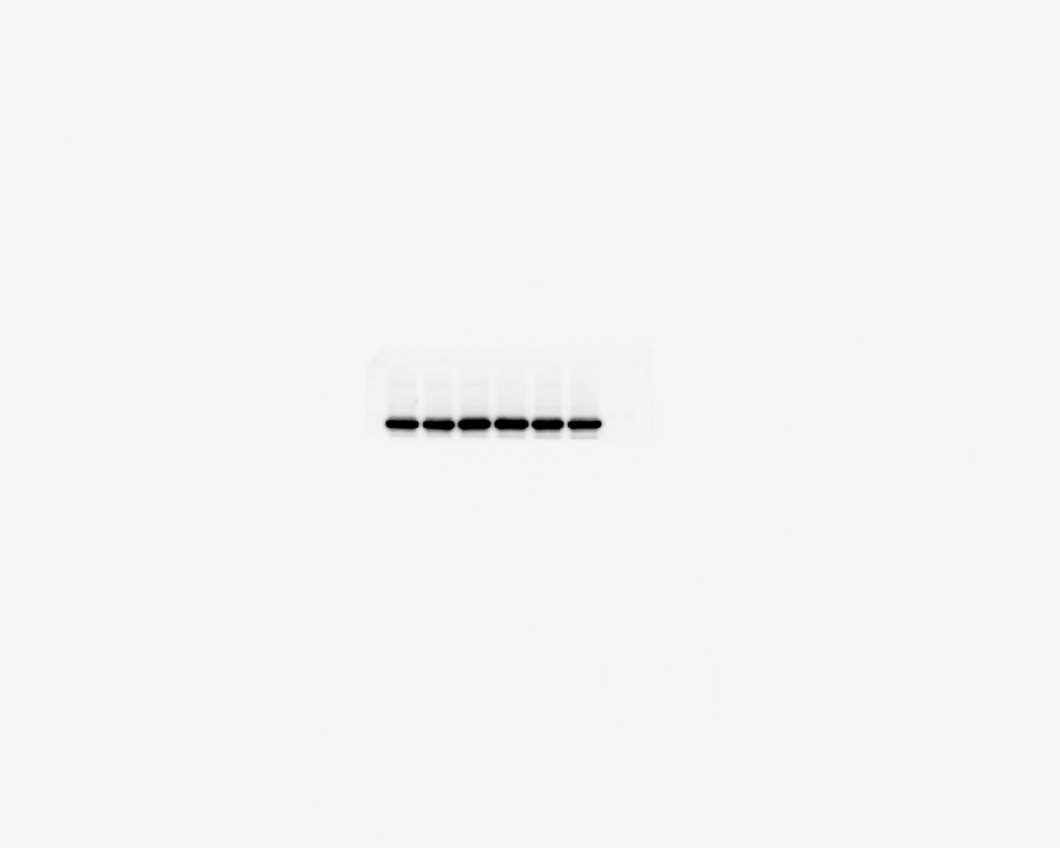

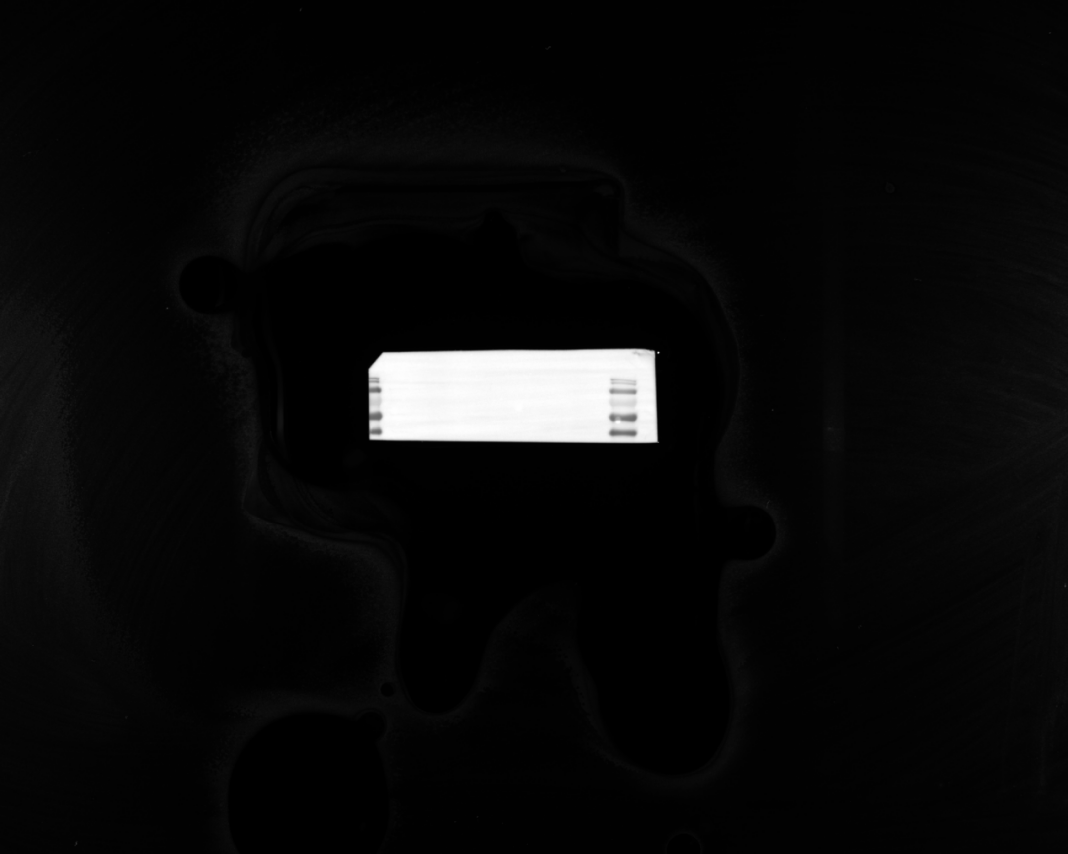


Β-actin


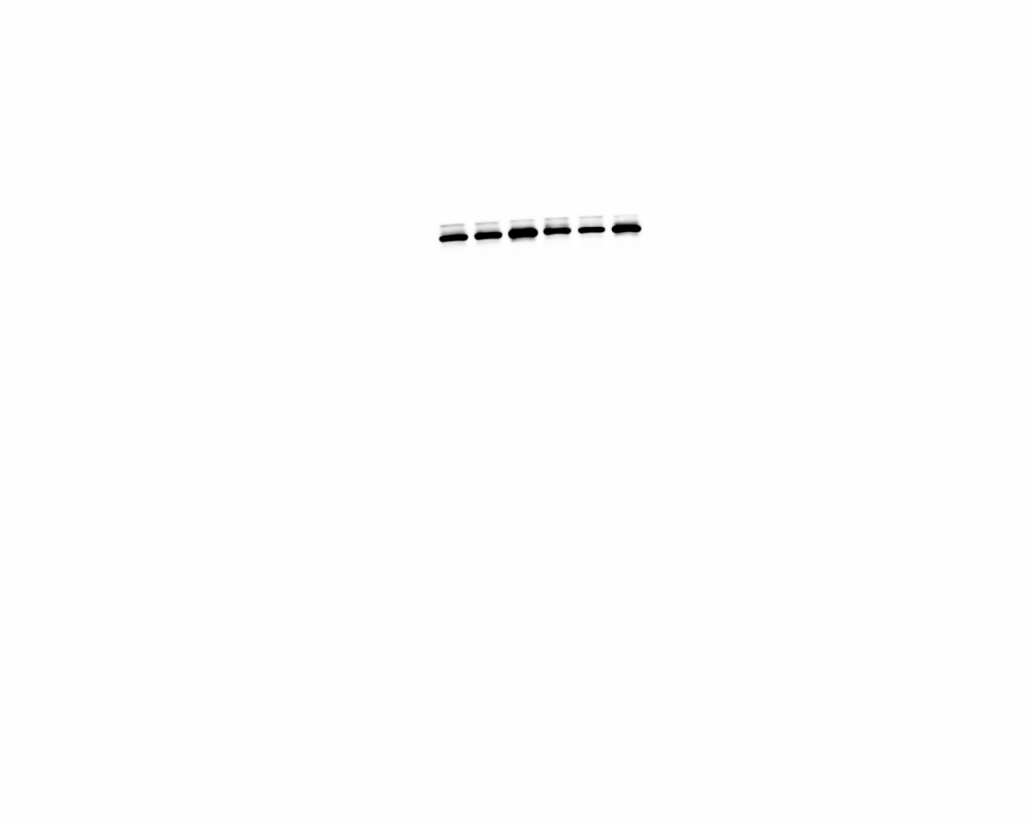

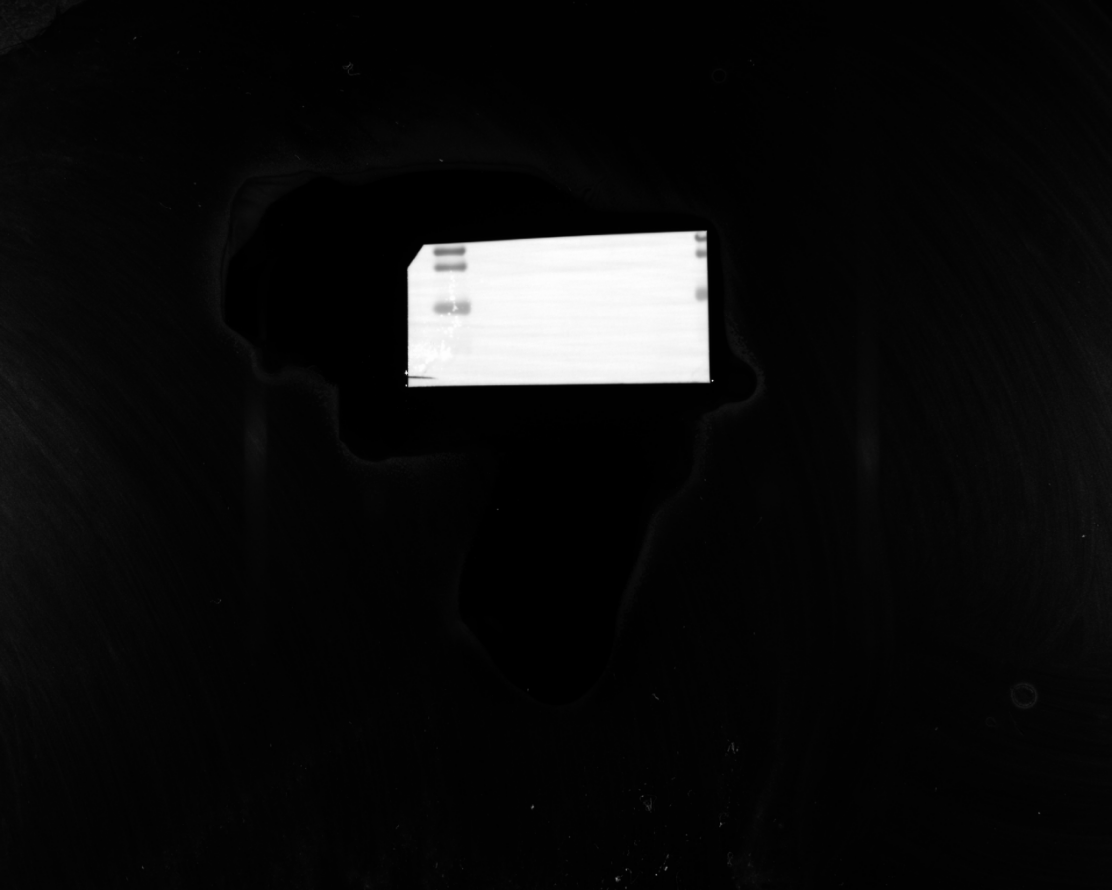


GPX4


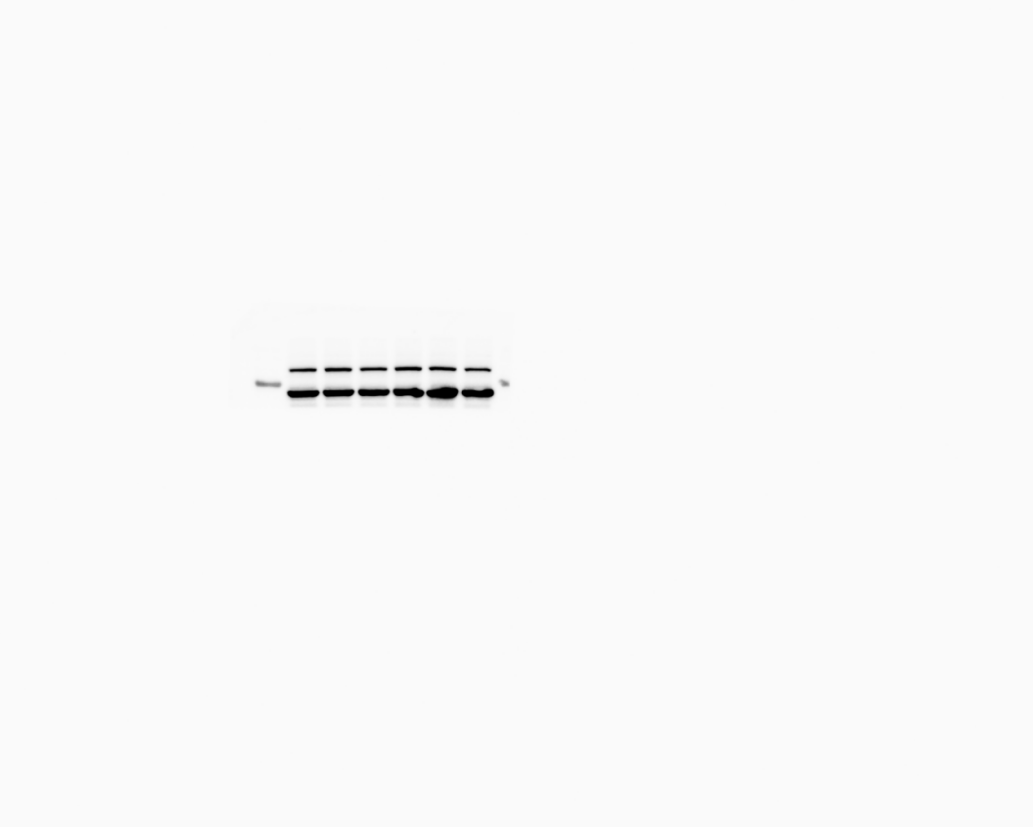

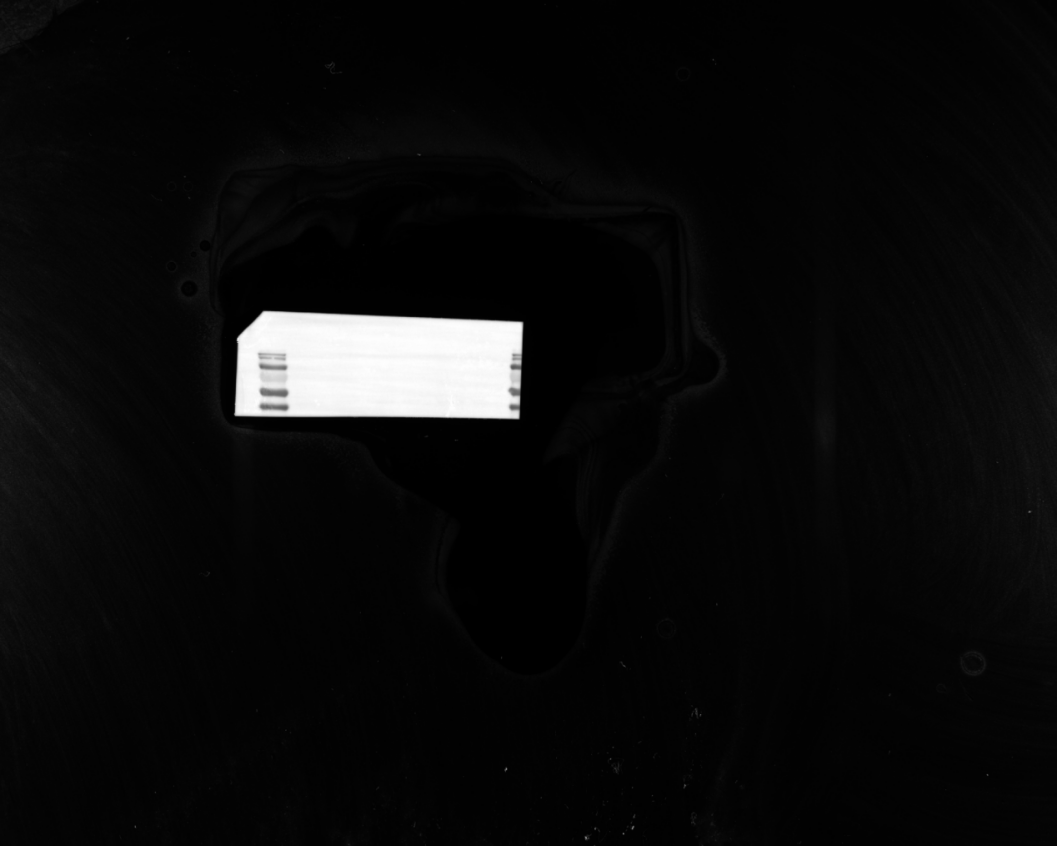


Β-actin


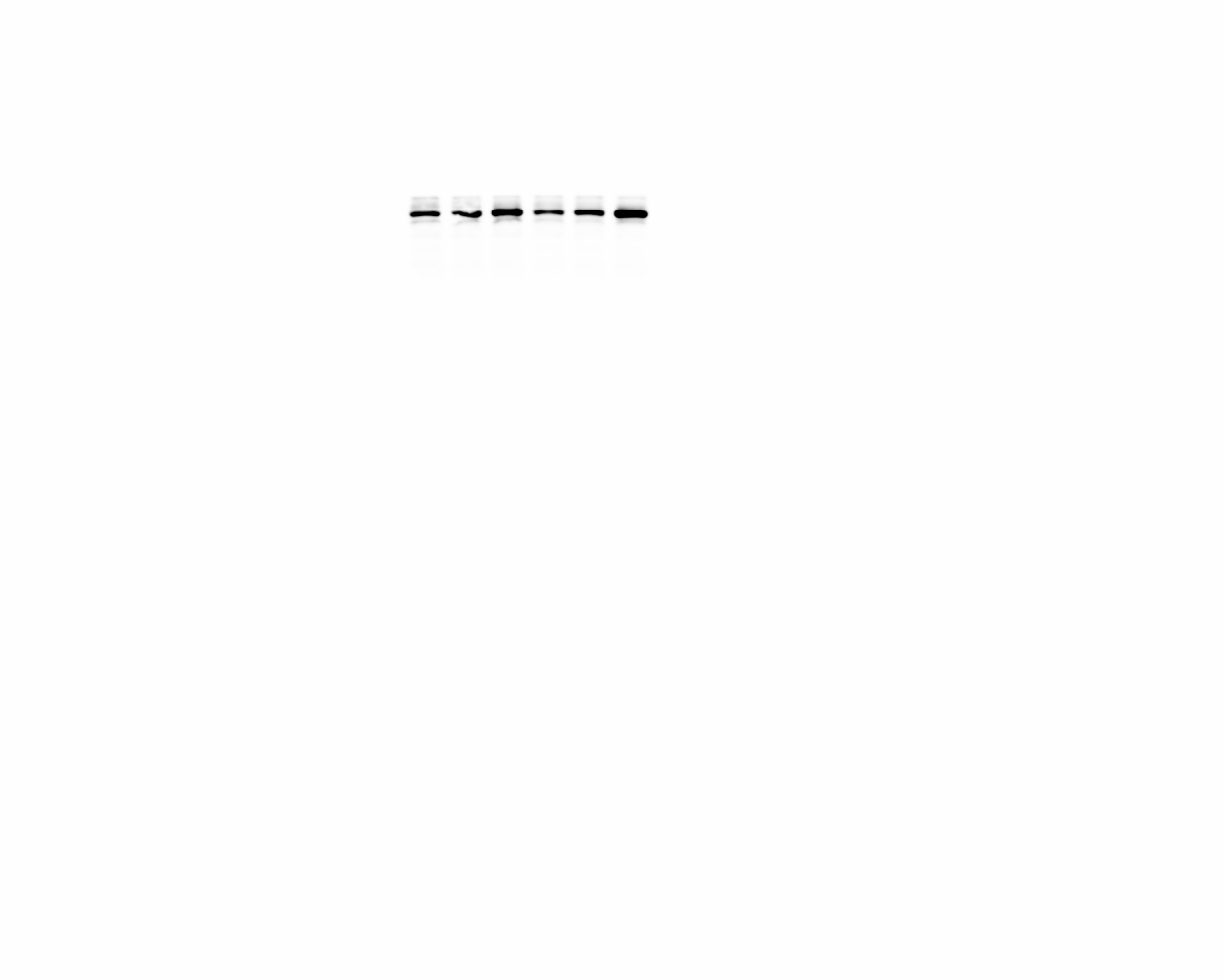

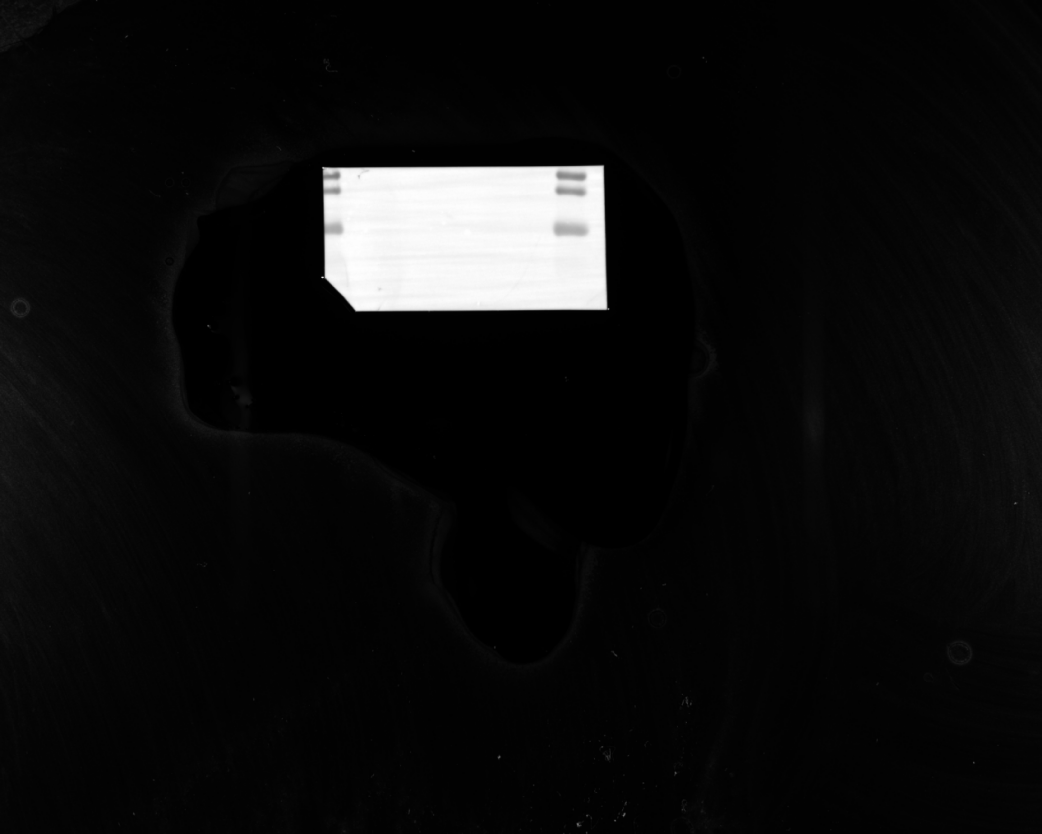


GPX4


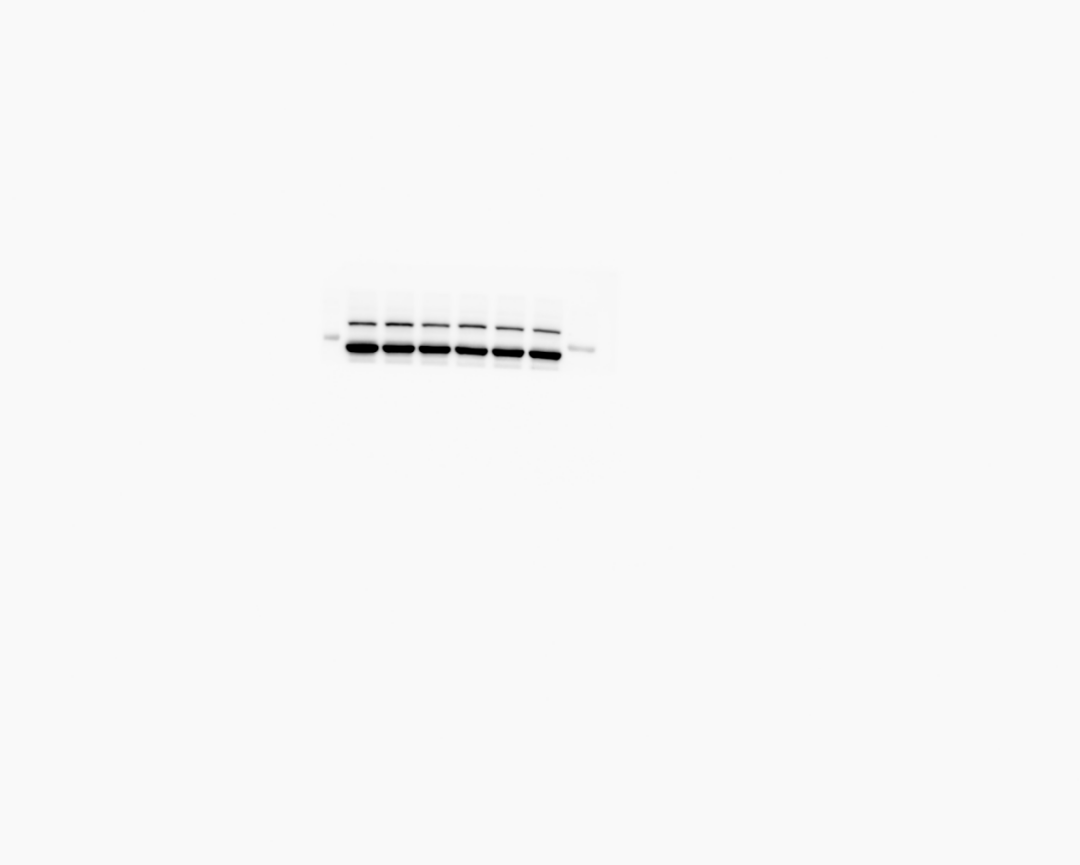

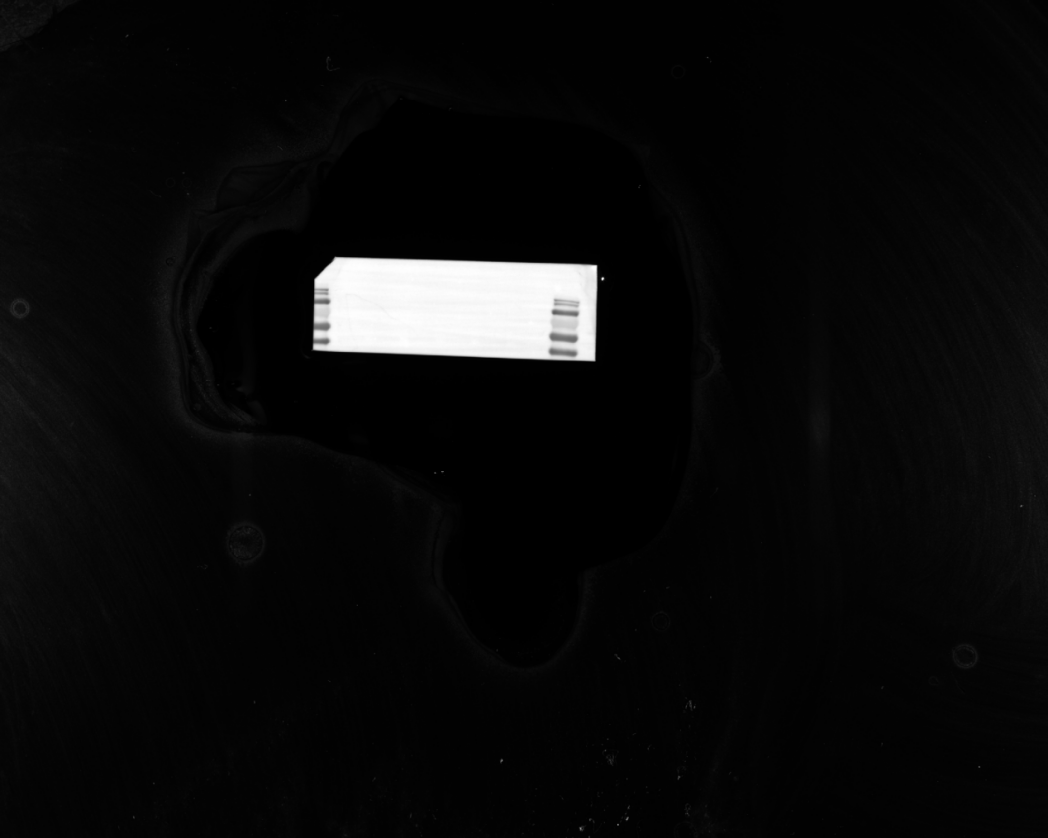


Β-actin


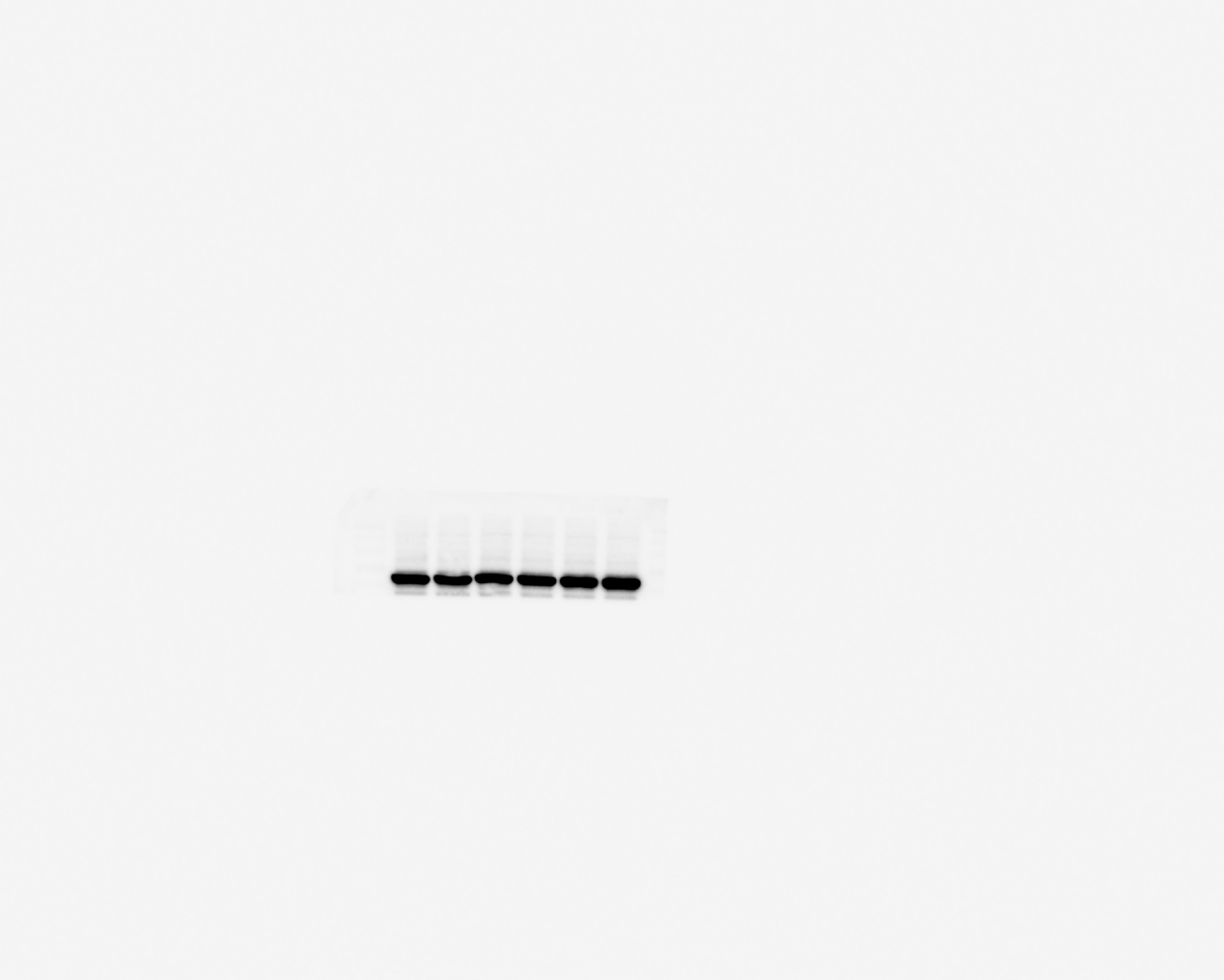


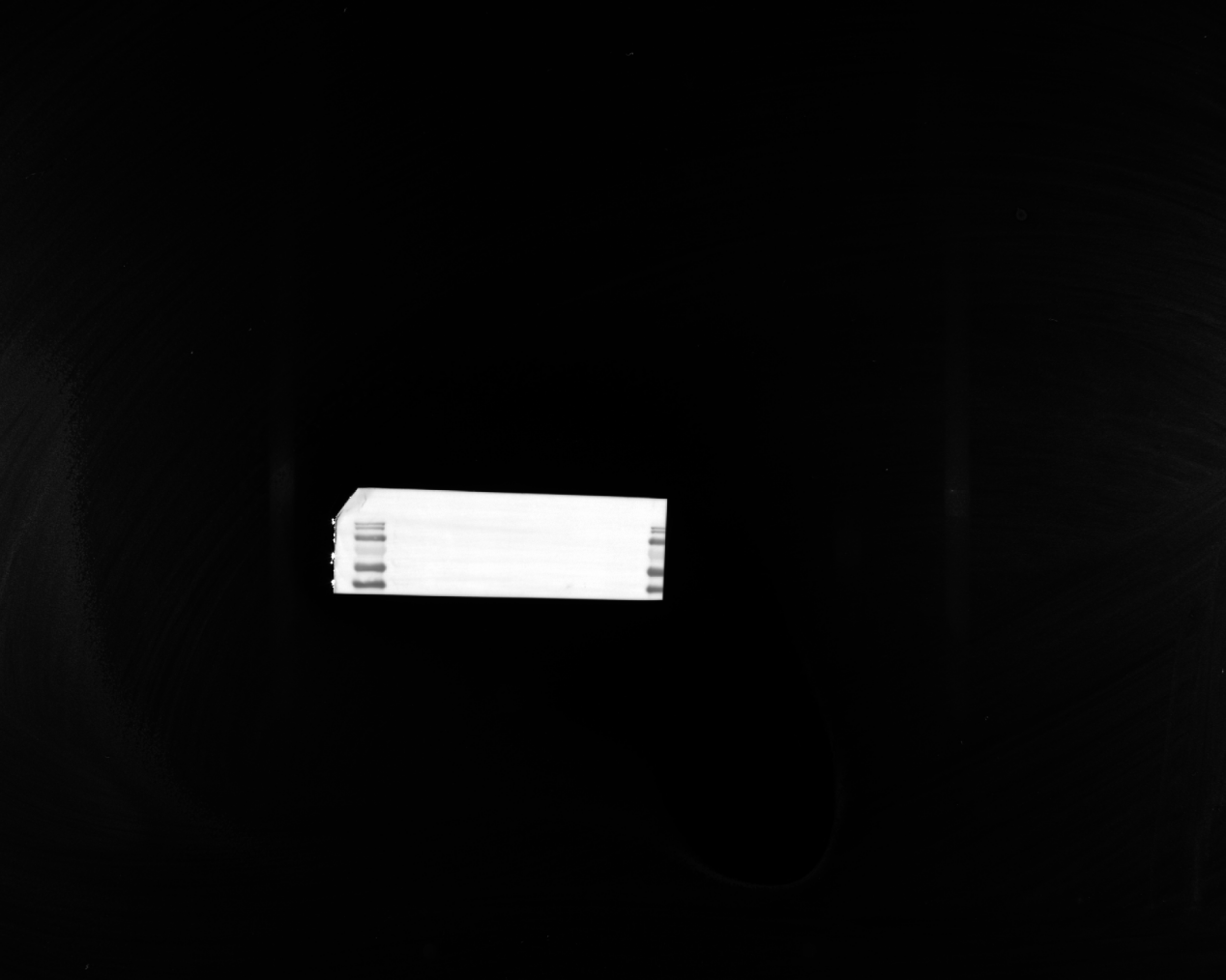


Β-actin


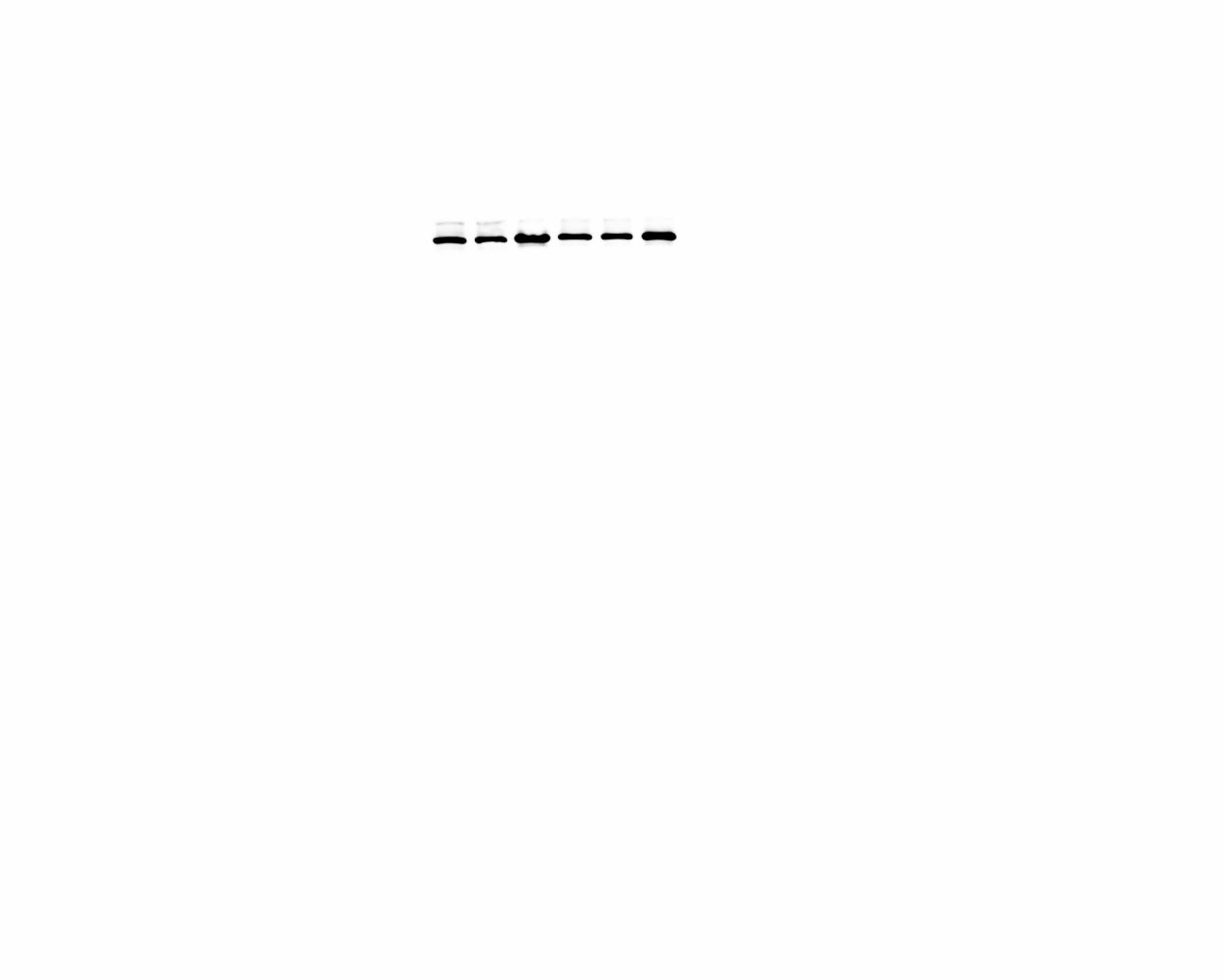


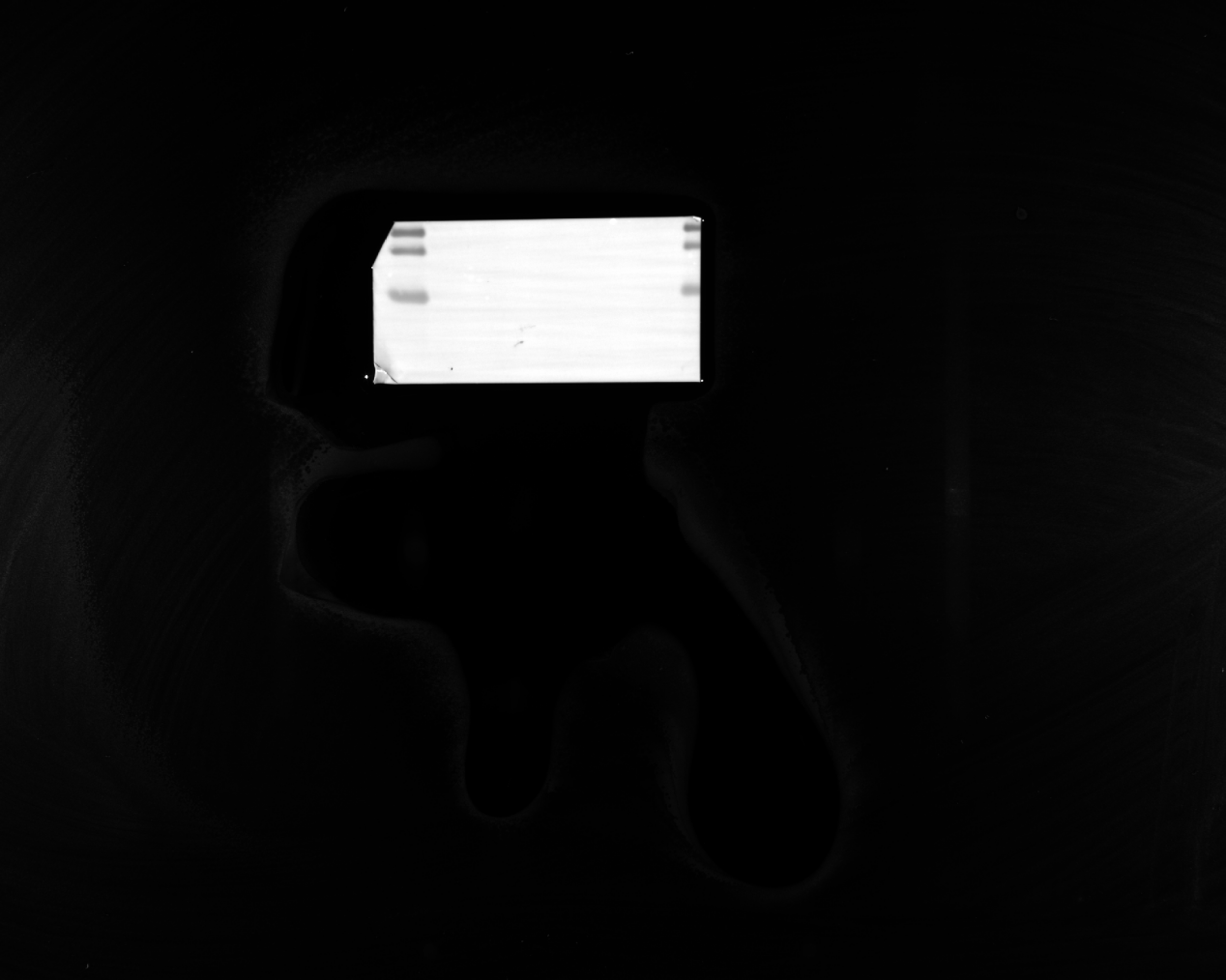


GPX4

CO-IP


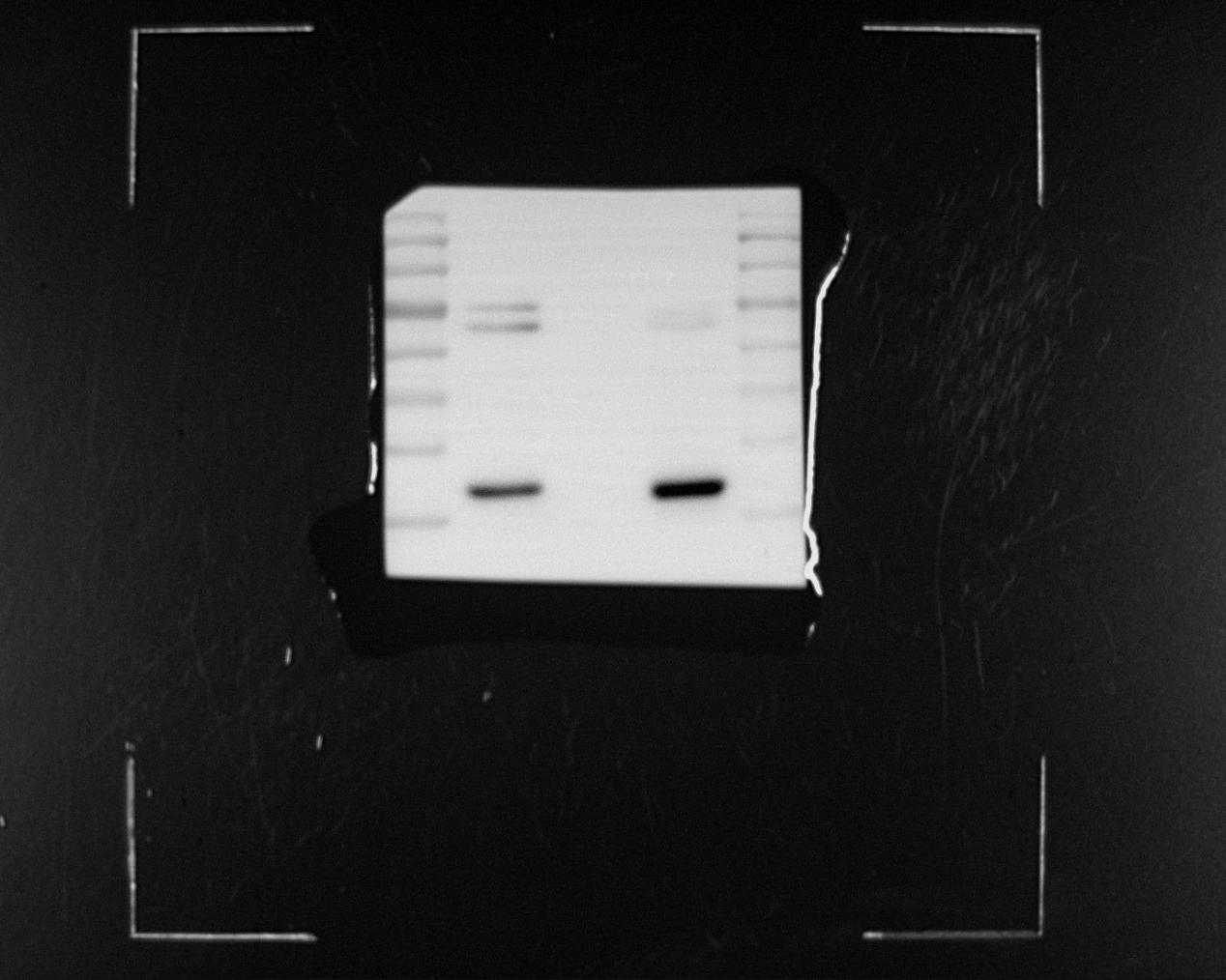

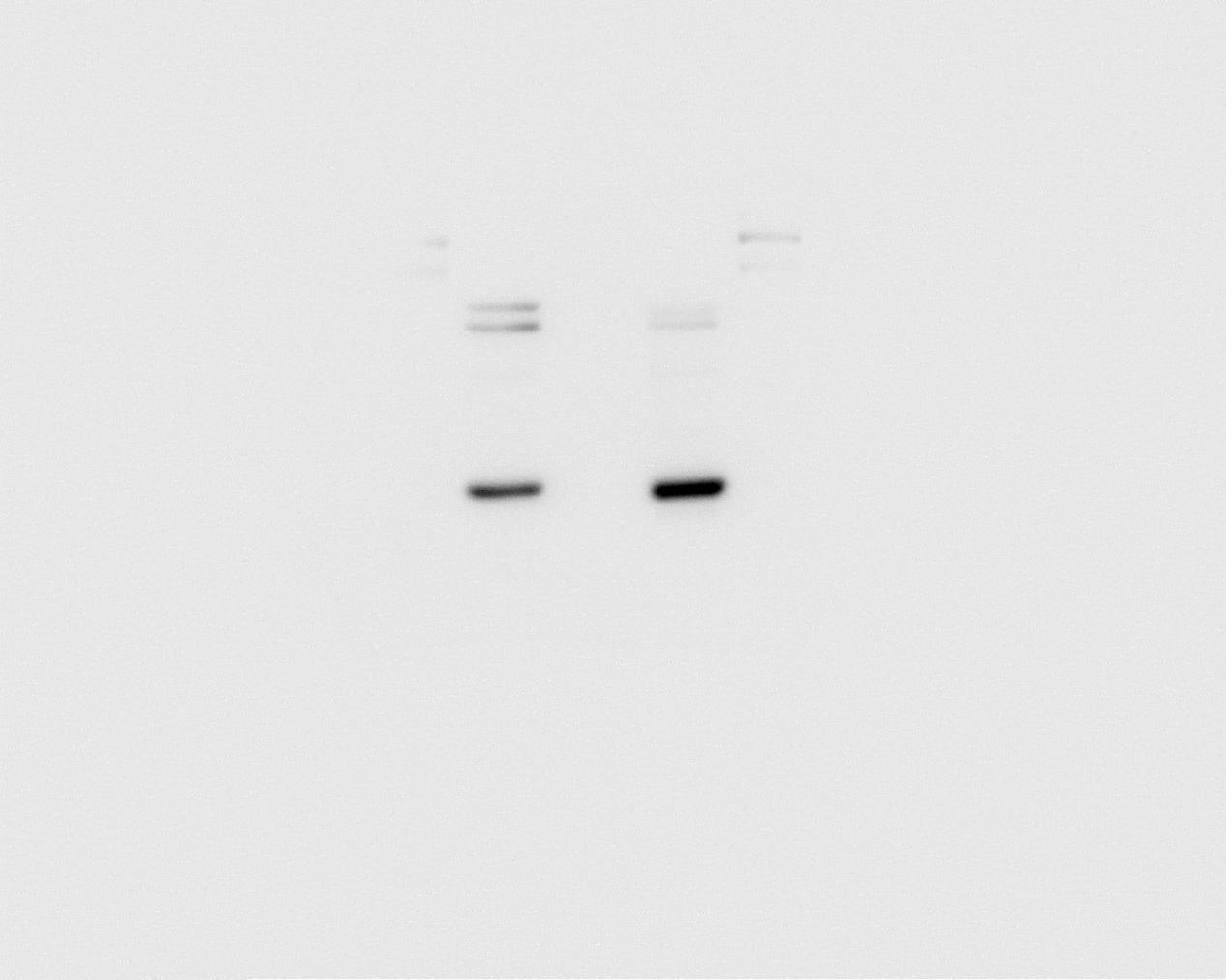

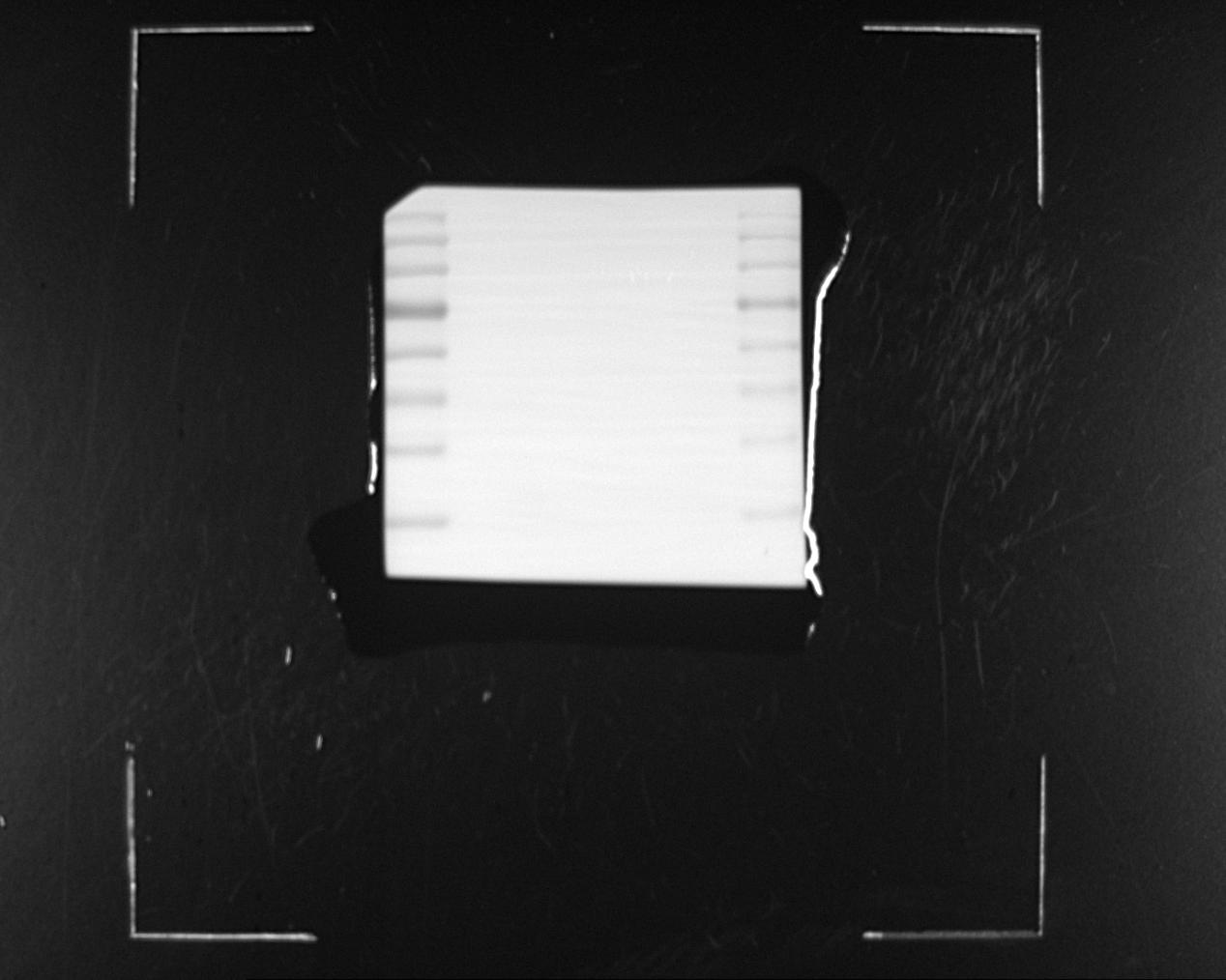

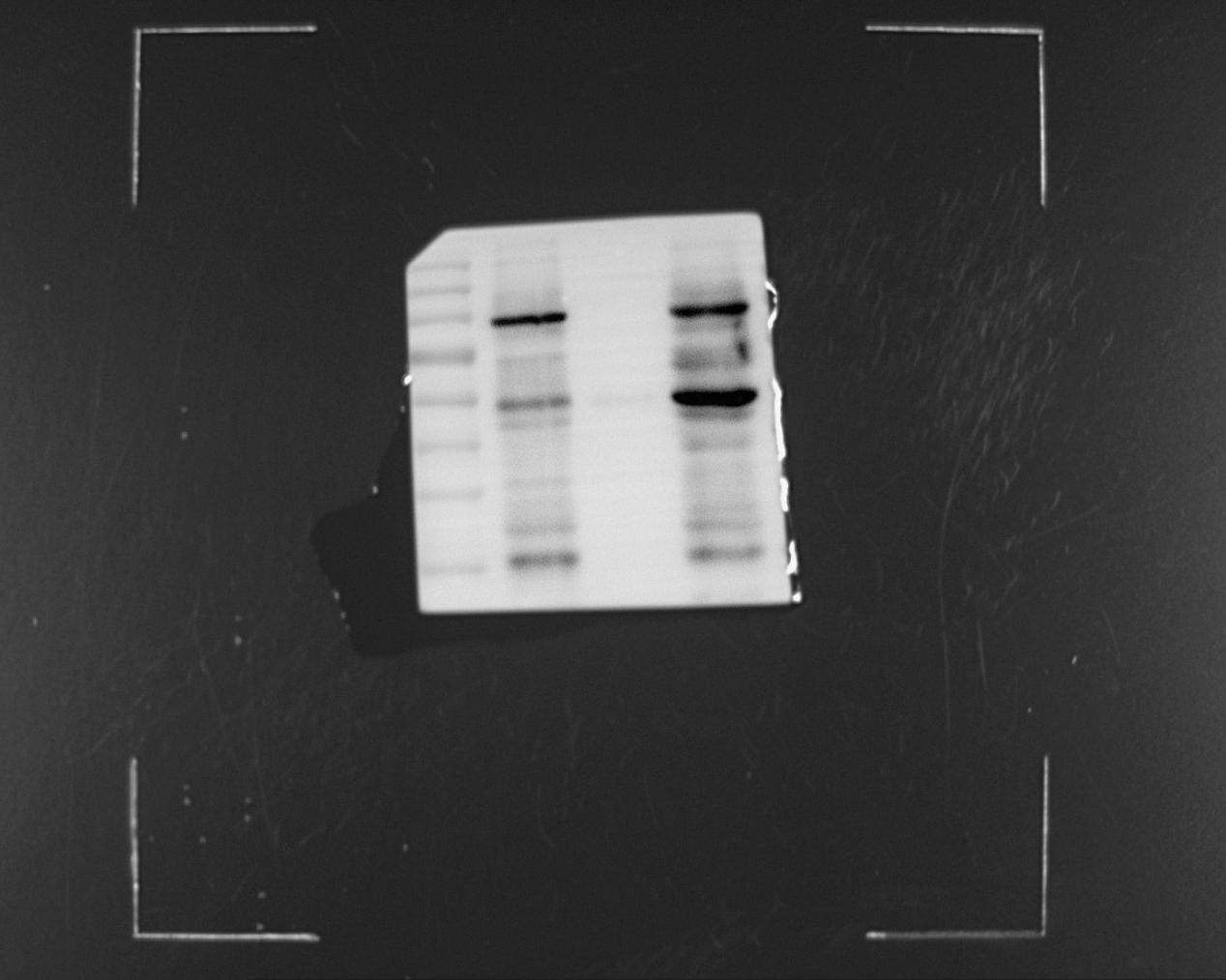

Supplement: Supplementary file 1 — Supplementary Material 1 [file 41598_2025_10414_MOESM1_ESM.docx]
